# Oxidative *N*-Formylation of Secondary Amines Catalyzed by Reusable Bimetallic AuPd–Fe_3_O_4_ Nanoparticles

**DOI:** 10.3390/nano11082101

**Published:** 2021-08-18

**Authors:** Sabyuk Yang, Ahra Cho, Jin Hee Cho, Byeong Moon Kim

**Affiliations:** Department of Chemistry, College of Natural Sciences, Seoul National University, Seoul 08826, Korea; diablopkpk@snu.ac.kr (S.Y.); araelliecho@snu.ac.kr (A.C.); 201722658@snu.ac.kr (J.H.C.)

**Keywords:** formylation, gold nanoparticle, heterogeneous catalysis, methanol, bimetallic catalyst, recyclable catalyst

## Abstract

Bimetallic catalysts are gaining attention due to their characteristics of promoting reactivity and selectivity in catalyzed reactions. Herein, a new catalytic *N*-formylation of secondary amines using AuPd–Fe_3_O_4_ at room temperature is reported. Methanol was utilized as the formyl source and 1.0 atm of O_2_ gas served as an external oxidant. The bimetallic catalyst, consisting of Au and Pd, makes the reaction more efficient than that using each metal separately. In addition, the catalyst can be effectively recycled owing to the Fe_3_O_4_ support.

## 1. Introduction

**Scheme 1:**
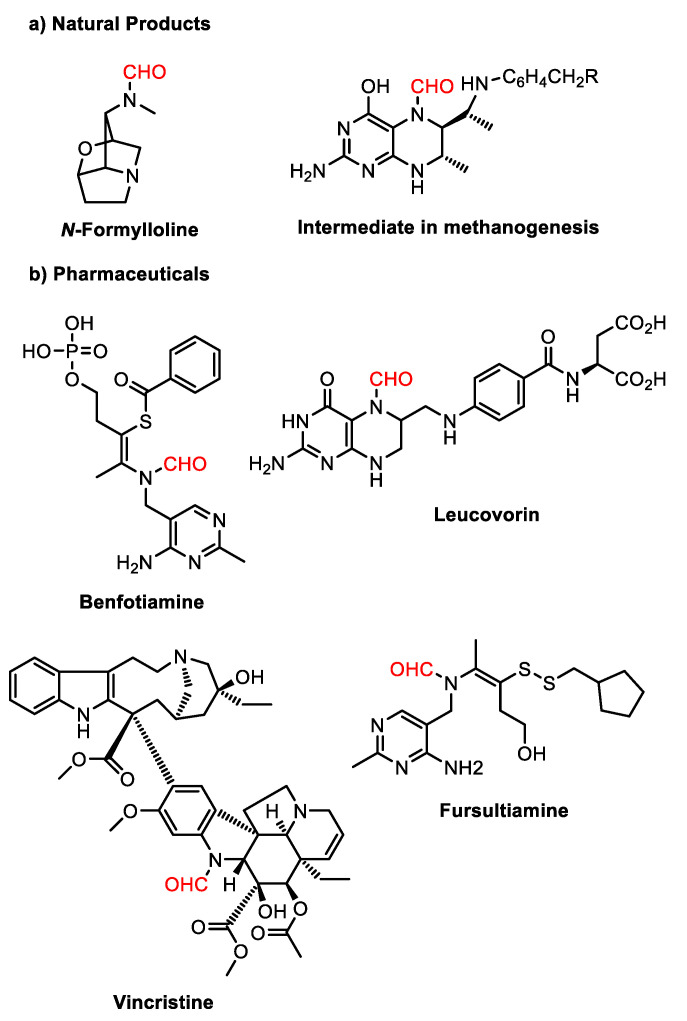
Formamide groups found in natural products and pharmaceuticals: (**a**) natural products; (**b**) pharmaceuticals.

Formamide groups are important in organic chemistry because of their abundance in natural products or utility as a valuable intermediate in synthesis (Scheme 1a), and pharmaceuticals (Scheme 1b) [1]. For example, this moiety is found in *N*-formylloline, an alkaloid produced by grass [2], and acts as an intermediate in biochemical processes, such as methanogenesis [3]. Some examples of formamide-containing drugs are benfotiamine (diabetic neuropathy) [4], leucovorin (toxic effects of methotrexate and pyrimethamine) [5], vincristine (anticancer) [6], and fursultiamine (thiamine deficiency) [7]. Considering the prevalence and importance of the formamide functionality, many researchers have developed efficient methods for attaching a formyl group to an amine [8,9,10]. One of the most direct methods to synthesize formamides is the direct *N*-formylation of amines with a formate ester, organosilicon reagent, formic acid or cyanide [8,9,11,12,13,14]. However, this process generates halide byproducts, and more environmentally friendly methods using green formyl sources have been actively pursued [15,16,17].

Among formyl sources, methanol is an ideal C_1_ source because of its availability and ecofriendly properties compared with other reagents [18,19]. It is one of the most popular C_1_ sources in organic reactions, such as methylation [20,21,22,23,24], formylation [25,26,27], and methoxylation [28,29,30], forming C–C, C–N, and C–O bonds, respectively. Therefore, developing a new catalytic route for the synthesis of formamides with methanol as a sustainable building block would be a valuable addition to the synthetic toolbox.

**Scheme 2:**
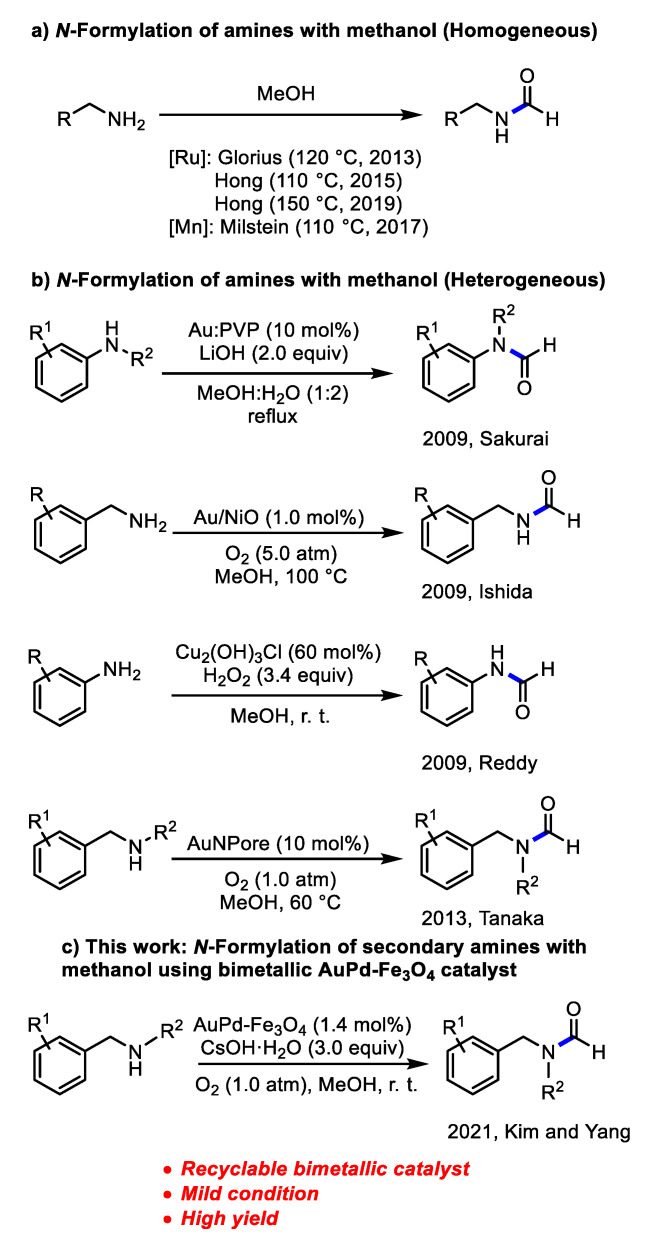
*N*-Formylation of amines using methanol as a C_1_ source: (**a**) homogeneous catalysis; (**b**) heterogeneous catalysis; (**c**) This work.

Currently, there are a handful of catalytic *N*-formylation reactions of amines with methanol in the literature (Scheme 2). Both homogeneous and heterogeneous catalysts have been widely used for this transformation. Homogeneous catalysts are one of the most popular tools, owing to their high reactivities (Scheme 2a) [31,32,33]. For example, Glorius and coworkers reported the *N*-formylation of amines catalyzed by the Ru–NHC catalyst in 2013 [34]. In 2017, the Milstein group achieved the acceptorless dehydrogenative coupling of methanol and amines using a manganese catalyst [35]. Hong and coworkers reported the *N*-formylation of amines with methanol in the presence of ruthenium catalysts in 2015 and 2019 [36,37]. In addition to these representative examples, various transition metal catalysts have been employed in *N*-formylation [38,39,40,41,42,43,44]. However, despite their merits, homogeneous catalysts are generally difficult to recover and show low stability compared to heterogeneous catalysts. Therefore, heterogeneous catalysts have been considered as alternatives because of their recyclability and ease of handling (Scheme 2b) [26,45,46,47]. Au-containing heterogeneous catalysts have often been employed as highly efficient catalysts for the oxidation of alcohols and their coupling reactions with other reactants [48,49,50,51].

For example, in 2009, Sakurai and coworkers reported the *N*-formylation of anilines using Au nanoclusters stabilized by poly(*N*-vinylpyrrolidone) (Au:PVP) [52]. In the same year, Ishida et al. developed the *N*-formylation of benzylamine using supported Au nanoparticles, such as Au/NiO or Au/Al_2_O_3_ [53]. Using hydrogen peroxide as an oxidant, Reddy developed the room-temperature *N*-formylation of amines using a copper catalyst [54]. In 2013, the reaction of aliphatic amines using AuNPore under 1.0 atm of O_2_ gas was developed by Tanaka et al. [55]. However, most of these reactions require high temperatures or high O_2_ gas pressures. Therefore, the development of a simple experimental procedure under relatively mild conditions is still required.

Based on our continuous efforts toward the development of metal nanoparticle–Fe_3_O_4_ catalysts, such as Pd–Fe_3_O_4_ [56], we developed a series of heterogeneous transition metal nanocatalysts for efficient organic transformations [57,58,59,60,61,62]. In particular, bimetallic combinations of various transition metals on an Fe_3_O_4_ support have been very useful catalysts for efficient, selective organic transformations [63,64,65,66,67]. Because Au nanoparticle catalysts have been widely used in a variety of oxidative reactions, we prepared bimetallic AuPd–Fe_3_O_4_ nanoparticle catalysts for selective redox reactions and developed the efficient reductive amination of nitroarenes and aldehydes [65] and oxidative conversion of 5-hydroxymethylfurfural to furan-2,5-dimethylcarboxylate [66].

Herein, we report a new and efficient protocol for secondary amine *N*-formylation through methanol oxidation, using AuPd–Fe_3_O_4_ as a reusable catalyst (Scheme 2c). Methanol serves not only as the formylating agent, but also as the solvent, and the reaction proceeds at room temperature under 1.0 atm of O_2_ gas as an external oxidant.

## 2. Materials and Methods

All commercially available chemicals were used as received without further purification. HAuCl_4_·3H_2_O (+49% Au basis) and PdCl_2_ (99% purity) were purchased from Alfa Aesar (Ward Hill, MA, USA). *N*-methyl-1-phenylmethanamine and polyvinylpyrrolidone (PVP) were purchased from Sigma-Aldrich (St. Louis, MO, USA). Cesium hydroxide monohydrate was purchased from Acros Organics (Pittsburgh, PA, USA). Fe_3_O_4_ NPs were purchased from DK nano technology (Beijing, China).

## 3. Results

### 3.1. Catalyst Characterization

**Figure 1:**
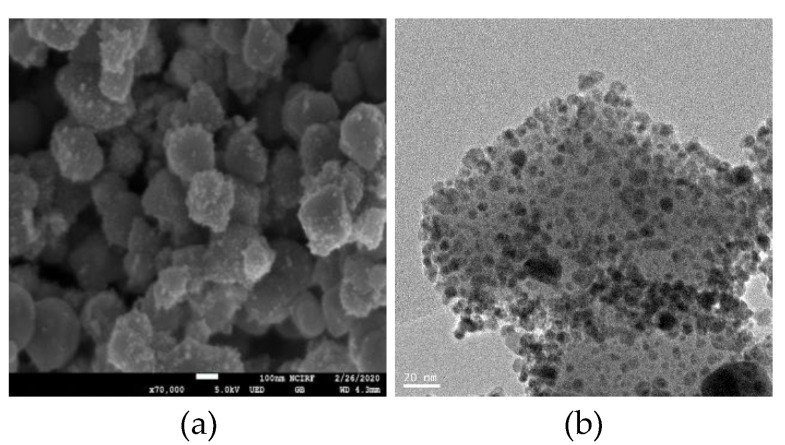
(**a**) SEM and (**b**) HR-TEM image of AuPd–Fe_3_O_4_ catalyst.

**Figure 2:**
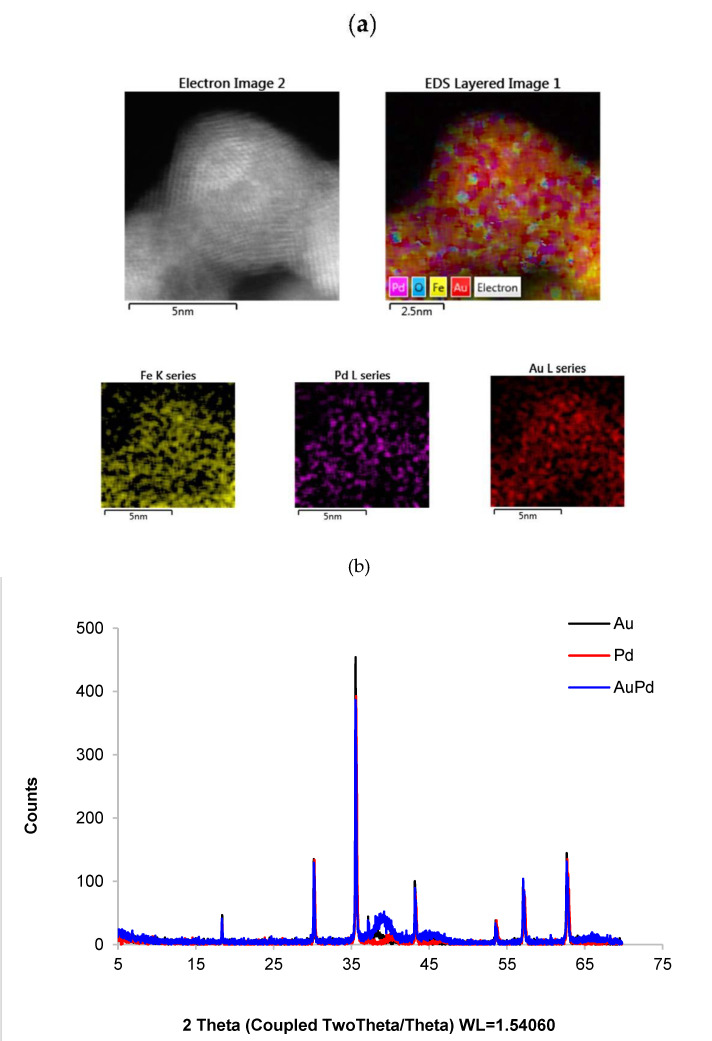
(**a**) STEM-EDS image and (**b**) XRD data of AuPd–Fe_3_O_4_ catalyst.

The bimetallic AuPd–Fe_3_O_4_ catalyst was synthesized following a procedure previously developed in our laboratories [65]. Adding palladium(II) chloride (PdCl_2_) in ethylene glycol, gold(III) chloride trihydrate (HAuCl_4_·3H_2_O) in water, and aqueous sodium borohydride solution dropwise sequentially to a Fe_3_O_4_ solution in water and stirring under 60 °C for 5 h afforded AuPd–Fe_3_O_4_ nanoparticles. These were characterized by scanning electron microscopy (SEM, Figure S1) by JSM-7800F Prime (JEOL Ltd., Tokyo, Japan), energy-dispersive X-ray spectroscopy (EDS, Figures S2 and S3) by JSM-7800F Prime (JEOL Ltd., Tokyo, Japan), high-resolution transmission electron microscopy (HR-TEM, Figures S4 and S6) by JEM-3010 (JEOL Ltd., Tokyo, Japan), scanning transmission electron microscopy (STEM, Figure S5) by JEM-ARM200F (JEOL Ltd., Tokyo, Japan), and X-ray photoelectron spectroscopy (XPS, Figure S7) by AXIS SUPRA (Kratos Analytical Ltd., Manchester, UK). The binding energy peaks of Pd 3d in Pd–Fe_3_O_4_ were positioned at 339.8 and 334.6 eV, corresponding to Pd(0) species. Au 4f peaks appeared at 86.9 and 83.3 eV for Au–Fe_3_O_4_, indicating the presence of Au(0) species. For AuPd–Fe_3_O_4_, the Pd 3d peaks appeared at 339.6 and 334.3 eV and the Au 4f peaks appeared at 86.8 and 83.1 eV, slightly lower than those of the corresponding monometallic nanoparticles. This phenomenon appears to stem from the interaction of Pd and Au, changing the electronic structure because of the formation of an AuPd alloy [68,69,70,71]. The lowered binding energy of Au(0) is expected to result from the electron transfer from Pd to Au. However, the reason why the Pd(0) peak shifted to a slightly lower value is not fully understood. SEM, HR-TEM, and STEM images show that Au and Pd were successfully deposited as an alloy on the iron oxide support (Figure 1 and Figure 2a and Figure S5), with the Au and Pd atoms randomly distributed. AuPd–Fe_3_O_4_ X-ray diffraction (XRD) data obtained by D8 Advance (Bruker, Billerica, MA, USA) compared with those of both Au–Fe_3_O_4_ and Pd–Fe_3_O_4_ revealed that the AuPd–Fe_3_O_4_ is made up with Au−Pd bimetallic alloy dispersed on the Fe_3_O_4_ surface (Figure 2b and Figure S9). Inductively coupled plasma-atomic emission spectroscopy (ICP-AES) data obtained by OPTIMA 8300 (Perkin-Elmer, Waltham, MA, USA) revealed that the AuPd–Fe_3_O_4_ nanoparticles (NPs) consist of 8.92 wt% Au and 5.19 wt% Pd at a molar ratio of 1.00:1.08 (Figure S8).

### 3.2. Reaction Optimization

Following successful preparation of AuPd–Fe_3_O_4_, we sought the optimum reaction conditions for the *N*-formylation of amines under oxidative conditions. We chose methanol as the formyl group source because it can be readily oxidized to formaldehyde, forming an hemiaminal upon reaction with an amine. We hypothesized that the resulting hemiaminal could be further oxidized to an *N*-formyl group under catalytic oxidation conditions with AuPd–Fe_3_O_4_ catalyst.

Because the formylation of amine by methanol requires the oxidation of methanol to an hemiaminal and subsequent dehydrogenation of the hemiaminal, an appropriate oxidant should be selected. Various oxidants have been used for alcohol oxidation [72,73,74]. O_2_ gas was chosen as the oxidizing agent to avoid chemical waste problems [75,76,77,78,79,80,81].

**Scheme 3:**
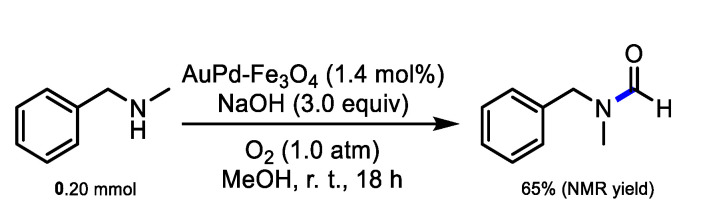
Initial observation of formamide under AuPd–Fe_3_O_4_.

Initially, a primary amine, such as benzylamine, was mixed with 1.4 mol% of AuPd–Fe_3_O_4_ (2.8 mol% of total metal contents except for Fe) and methanol under 1.0 atm of O_2_ gas, but no *N*-benzylformamide product was detected, while the starting material remained. Subsequently, we added 3.0 equivalents of sodium hydroxide, which led to the formation of *N*-benzylformamide in 25% yield after 18 h. In addition, several unknown high-molecular-weight products, along with a small amount of benzonitrile, were detected from liquid chromatography-mass spectrometry (LC-MS) analysis of the crude mixture. These results suggest that the base plays an important role in the conversion of the amine substrate. According to a report from Mallat group [75], bases are known to facilitate hydrogen abstraction in alcohol dehydrogenation with Au catalysts. However, because the reaction of a primary amine did not yield a clean formylation product, we focused our attention on the formylation of secondary amines. Our first reaction of *N*-methyl-1-phenylmethanamine with methanol, in the presence of AuPd–Fe_3_O_4_ with sodium hydroxide as a base, yielded the desired *N*-benzyl-*N*-methylformamide in 65% NMR yield (Scheme 3).

Based on these initial findings, various reaction conditions were tested, and the results are presented in Table 1 and Tables S1– S3. Using *N*-methyl-1-phenylmethanamine (**1a**) as a standard substrate, we tested various reaction conditions with methanol as the formylating agent. Using only iron oxide as a catalyst and sodium hydroxide as a base resulted no progress of the reaction at all (entry 1). However, a reaction with 2.8 mol% Pd–Fe_3_O_4_ catalyst furnished *N*-benzyl-*N*-methylformamide (**2a**) at a 55% yield (entry 2). Reactions with other monometallic catalysts, such as Au–Fe_3_O_4_, showed low reactivity (entry 3). The use of the AuPd–Fe_3_O_4_ bimetallic catalyst afforded the product in 65% yield (entry 4). Without a base, the reaction was sluggish (entry 5). When the O_2_ balloon was replaced with an air balloon, the reactivity lowered to afford the desired product at only 43% yield (entry 6). By changing the base to cesium carbonate, we observed a yield similar to that of the reaction using sodium hydroxide (entry 7). However, other inorganic bases were not as effective as hydroxide bases. The reaction using cesium hydroxide monohydrate afforded 90% yield under the same conditions (entry 8). Furthermore, the reaction yield was maintained when the reaction was scaled up to 0.50 mmol (entry 9). The reaction was complete in 4 h when the substrate concentration was increased from 0.20 to 1.0 M, resulting in 89% yield (entry 10). A reaction using ethanol instead of methanol afforded the acetylated product in 42% yield (entry 11). The catalyst loading could be low as 0.40 mol% without significant loss of yield, which is translated to high turnover number (TON) (entry 12 and Table S3).

Knowing that hydroxide bases were effective, we performed further investigations with bases (Table 2 and Table S2). Without a base, only 14% yield of **2a** was obtained (entry 1). Reactions employing LiOH·H_2_O and NaOH resulted in 60% and 65% yields, respectively, of the desired products (entries 2 and 3). The use of slightly stronger hydroxide bases, such as KOH or CsOH·H_2_O, ensured 75% and 91% yields, respectively (entries 4 and 5). However, carbonate bases were not as effective as hydroxide bases (entries 6 and 7). Reaction yields obtained with other bases, including KO*t*Bu, K_3_PO_4_, and CsF, were moderate (entries 8–10). In summary, CsOH·H_2_O was the optimal base.

### 3.3. Effect of Alloy Bimetallic Catalyst vs. Combination of Two Metal Catalysts

To investigate the distinctive advantage of the bimetallic nanocatalyst, several control experiments were conducted (Table 3). Because we used 1.4 mol% of AuPd–Fe_3_O_4_ to obtain the optimum results with **1a**, reactions with 2.8 mol% of either Au–Fe_3_O_4_ (entry 1) or Pd–Fe_3_O_4_ (entry 2) were performed under the standard reaction conditions to test the reactivity of each monometallic catalyst. The *N*-formylation reactions of *N*-methyl-1-phenylmethanamine, employing either Au or Pd catalysts, were not as efficient as that employing the AuPd catalyst (entry 4), giving 51% and 57% yields of **2a**, respectively. To further investigate the alloy nanocatalyst, we examined the reactions of other substrates, such as *N*-methyl-1-(*p*-tolyl)methanamine, 1-(4-methoxyphenyl)-*N*-methylmethanamine, *N*-methyl-1-(3-nitrophenyl)methanamine, and 3-(methylaminomethyl)benzonitrile. With 2.8 mol% of Au–Fe_3_O_4_ (entry 1) or 2.8 mol% of Pd–Fe_3_O_4_ (entry 2), the reactions of secondary amines showed lower yields than those using AuPd–Fe_3_O_4_ (entry 4). These results indicate that both Au and Pd within the alloy catalyst contribute to product formation through a synergistic effect [82,83,84,85,86]. When we used 1.4 mol% each of both Au–Fe_3_O_4_ and Pd–Fe_3_O_4_, interestingly the reaction of *N*-methyl-1-phenylmethanamine afforded 90% yield of the desired product (entry 3). The fact that similar product yields were obtained from the reactions employing AuPd and the combination of the Au and Pd toward *N*-methyl-1-phenylmethanamine was an exception rather than a general trend, as can be seen in the reactions of various substituted *N*-methylarylmethanamines. Reactions of other substrates with both Au–Fe_3_O_4_ and Pd–Fe_3_O_4_ provided the corresponding products with much lower yields than those obtained with the bimetallic AuPd catalyst. To rule out the possibility of homogeneous catalysis with both Au and Pd leached out into the solution, we carried out a filtration experiment [59,60]. The solution from a reaction of *N*-methyl-1-phenylmethanamine with 1.4 mol% AuPd-Fe_3_O_4_ under the optimized condition after 0.5 h was filtered through a syringe filter. The filtrated solution was then stirred for 6 h under O_2_ atmosphere, and the progress of the reaction was checked. There was no further increase in the reaction yield. This result shows that the homogeneous solution did not drive the reaction further without the nanocatalyst, indicating that there is no catalysis from any homogeneous metal species. Next, we also tested the kinetics in the reaction of *N*-methyl-1-phenylmethanamine, employing either Au, Pd, a mixture of Au and Pd, or AuPd catalyst (Figure S14). The differences in the reaction rates indicate that there is a distinctive synergistic effect within the bimetallic catalyst, as the reaction employing this catalyst proceeded the fastest. Thus, considering both the product yields and the initial kinetics, there is an advantage in employing the bimetallic alloy catalyst, instead of using the monometallic catalysts separately.

### 3.4. Substrate Scope

**Scheme 4:**
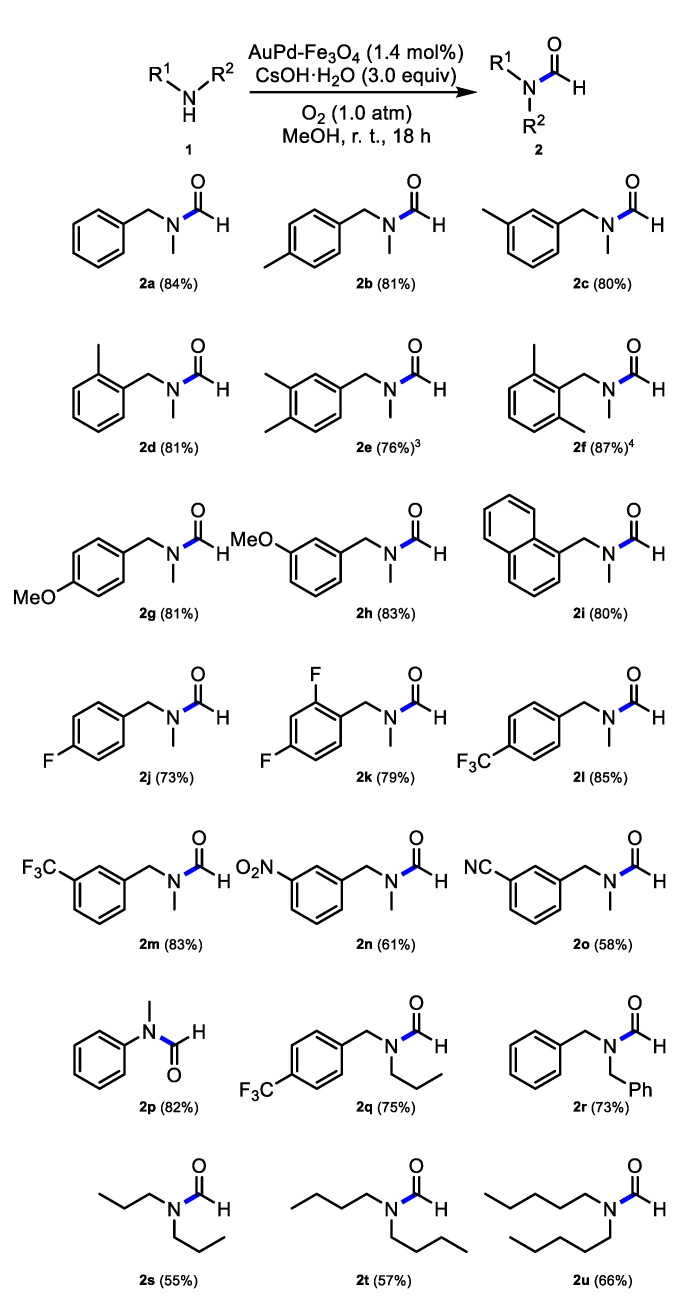
Substrate scope of *N*-formylation^1,2 1^ Isolated yield. ^2^ **1** (0.50 mmol), AuPd–Fe_3_O_4_ (1.4 mol%), CsOH·H_2_O (3.0 equiv), O_2_ (1.0 atm), methanol (2.0 mL), r. t., 18 h. ^3^ 2.4 mol% of AuPd–Fe_3_O_4_ used. ^4^ The reaction was run at 0.20 mmol scale of **1f** in methanol (1.0 mL).

Using the optimized condition, we tested the *N*-formylation of various secondary amines bearing substituted aromatic rings, using 0.25 M of substrate over 18 h (Scheme 4). From the reaction of unsubstituted *N*-methyl-1-phenylmethanamine, *N*-formylated product was obtained in 84% (**2a**). Substrates bearing a methyl group on the aromatic ring furnished a good product yield, regardless of its position (**2b**–**2d**). Reactivity was maintained in the case of a substrate with two methyl groups on the aromatic ring (**2e**). High yield (87%) was also observed from a sterically hindered substrate possessing methyl groups at positions 2 and 6 (**2f**) when the reaction was run at 0.20 M (0.20 mmol scale). The reactions of substrates possessing a strong electron-donating methoxy group resulted in good yields (81% and 83%, **2g** and **2h**, respectively). The reaction of *N*-methyl-1-(naphthalen-1-yl)methanamine also resulted in good yield (80%, **2i**). When an electron-withdrawing group exists on the aromatic ring, good to moderate yields of the desired products were obtained (85%, 83%, 61% and 58% for **2l**–**2o**, respectively). Reactions of substrates bearing fluorine(s) resulted in acceptable product yields (73% and 79% for **2j** and **2k**, respectively). Interestingly, reactions of amines with trifluoromethyl groups at the 3 or 4 position on the aromatic ring furnished good yields (85%, 83%, and 75% for **2l**, **2m**, and **2q**, respectively) compared with other electron-deficient amines. Reactions of substrates possessing other electron-withdrawing substituents, such as nitro- or cyano- groups, yielded products with 61% and 58% yields (**2n** and **2o**), respectively. In addition, the reaction of *N*-methylaniline resulted in the desired formylated product in 82% yield (**2p**). We confirmed that not only *N*-methylamines, but other *N*-alkyl-substituted secondary amines are also good substrates for the reaction, as shown in the case of **2q** and **2r**, with the reaction of *N*,*N*-dibenzylamine, resulting in 73% yield (**2r**). In the reactions of non-benzylic secondary alkylamines, products were obtained in moderate yields (55%, 57%, and 66%, **2s**–**2u**, respectively). We hypothesized that the reactions could be run at higher concentrations and thus expedited. When reactions were conducted at 1.0 M, most were completed in 6–8 h instead of 18 h at 0.20 M. The reactions of several substrates maintained their yields at higher concentrations in shorter reaction times, and the results are shown in the (supporting information Table S6).

### 3.5. Mechanistic Investigation

**Scheme 5:**
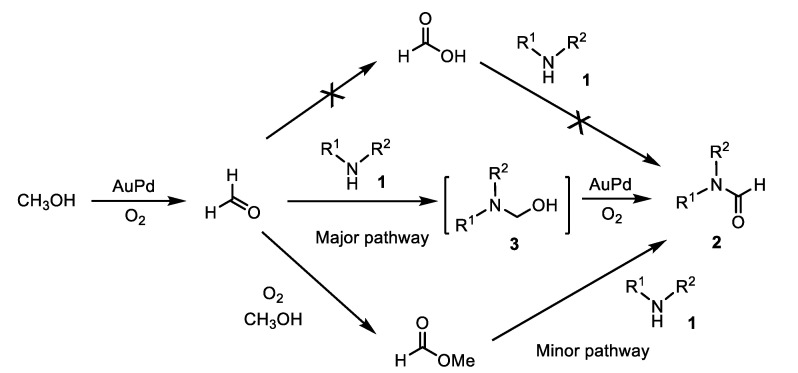
A plausible reaction pathway for *N*-formylation of amines.

Based on the results of optimization and the substrate scope investigation, we conducted several kinetic experiments and control experiments to probe the reaction mechanism (Figures S15 and S16, and Table S7). To summarize the results, the amine substrate (**1**) is consumed under oxidative condition in the presence of AuPd–Fe_3_O_4_ catalyst in methanol. The oxidation of methanol in the presence of the catalyst by the assistance of a base generates mostly HCHO and a small portion of HCO_2_Me, which rapidly react with an amine to furnish *N*-formamide (**2**). The formation of HCO_2_H from methanol was ruled out because almost no HCO_2_H was detected from the control oxidation of methanol. Both methanol and the resulting hemiaminal intermediate (**3**) are oxidized by the AuPd–Fe_3_O_4_ catalyst. These results are presented in Scheme 5.

### 3.6. Effect of Au:Pd Ratio and Various Supports

To further investigate the correlation between the Au:Pd ratio and the product yield, we synthesized catalysts with various Au:Pd ratios and tested them using 2.0 mol% of catalyst under the same reaction condition (Table 4 and Figure S10). The ~1:1 atomic ratio of Au and Pd was found to be the best combination. Additionally, to test the effect of iron oxide, we synthesized several AuPd catalysts with various supports for comparison (Tables S4 and S5 and Figure S13). AuPd on the iron oxide support showed the highest reactivity for *N*-formylation.

### 3.7. Recycling Test

**Figure 3:**
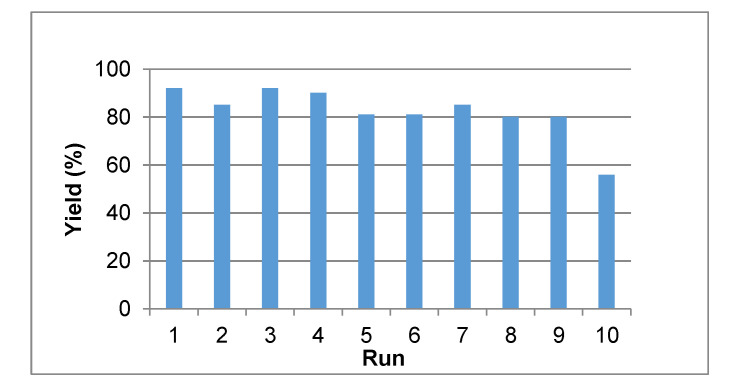
Results of recycling test.

Because heterogeneous catalysts are employed for easy recovery and reuse, the recyclability of AuPd–Fe_3_O_4_ was tested based on the optimized condition (Figure 3). In the recycling experiment with **1a**, the catalytic activity was maintained without significant loss of yield. Based on our previous report [66], a strong base disintegrates the iron oxide support, resulting in poor recyclability. However, the catalytic activity was somehow maintained for 9 cycles. When the SEM and HR-TEM images of the catalyst after 10 cycles were examined, Au and Pd particles appeared to be significantly agglomerated (Figures S11 and S12). Additionally, the ICP-AES data of the used catalyst showed lower Au and Pd contents (wt%) (Figure S8). Therefore, considering the excess amount of strong base and oxidative environment that can be detrimental to the catalyst, our reaction system has some reusability advantages.

### 3.8. Gram Scale Reaction

**Scheme 6:**
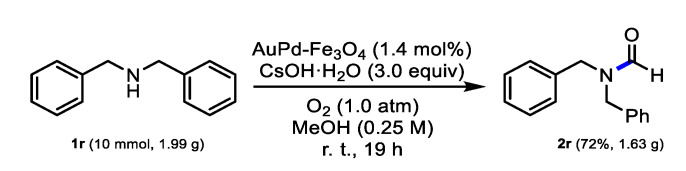
Gram scale reaction.

We subsequently performed a gram-scale reaction (Scheme 6). At a 10 mmol scale, the reaction of **1r** afforded **2r** in 72% yield, demonstrating the viability of the reaction in organic synthesis.

## 4. Conclusions

In conclusion, we developed a novel catalytic system for the mild synthesis of formamides in good-to-moderate yields. Utilizing a reusable AuPd bimetallic nanocatalyst, we synthesized formamides at room temperature under atmospheric pressure of O_2_. The use of methanol as the formyl source, instead of toxic or polluting reagents, bodes well for sustainable organic synthesis. During the investigation of various catalysts, the bimetallic alloy AuPd–Fe_3_O_4_ catalyst proved to be superior to either of the monometallic catalysts. Based on the product yield and initial kinetics, there appears to be a synergistic effect between the Au and Pd during the reaction. The broad substrate scope could enlarge the *N*-formylation reaction library. The formamide yield was the highest when the Au:Pd ratio was ~1:1. In addition, the AuPd on the Fe_3_O_4_ support proved to be more effective than AuPd catalysts on other supports. Furthermore, the reaction was scalable to the gram scale. This new method avoids the requirements of high temperature and high pressure of O_2_ gas. Moreover, the good recyclability of the catalyst broadens the potential applications of the reaction.

## Figures and Tables

**Table 1:**
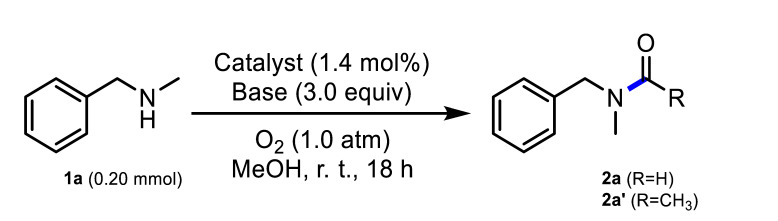
Screening data of the *N*-formylation of amine ^1^.

Entry	Catalyst	Base	TON ^2^	Yield ^3^
1	Fe_3_O_4_	NaOH	0	N. D. ^4^
2 ^5^	Pd–Fe_3_O_4_	NaOH	20	55
3 ^6^	Au–Fe_3_O_4_	NaOH	9	25
4	AuPd–Fe_3_O_4_	NaOH	23	65
5	AuPd–Fe_3_O_4_	-	5	14
6 ^7^	AuPd–Fe_3_O_4_	NaOH	15	43
7	AuPd–Fe_3_O_4_	Cs_2_CO_3_	25	69
8	AuPd–Fe_3_O_4_	CsOH·H_2_O	33	90 ^8^
9 ^9^	AuPd–Fe_3_O_4_	CsOH·H_2_O	33	92 (84 ^10^)
10 ^11^	AuPd–Fe_3_O_4_	CsOH·H_2_O	32	89
11 ^1^^2^	AuPd–Fe_3_O_4_	CsOH·H_2_O	15	42 ^13^
12 ^14^	AuPd–Fe_3_O_4_	CsOH·H_2_O	89	71

^1^ Reaction conditions: **1a** (0.20 mmol), catalyst (1.4 mol%), base (3.0 equiv), O_2_ (1.0 atm), methanol (1.0 mL), r. t., 18 h. ^2^ Turnover number (TON) = mmol of product/mmol of total metal except Fe. ^3^ Determined from ^1^H NMR spectral analysis through the use of mesitylene as an internal standard. ^4^ N. D. = not detected. ^5^ Pd–Fe_3_O_4_ (2.8 mol%) was used as a catalyst. ^6^ Au–Fe_3_O_4_ (2.8 mol%) was used as a catalyst. ^7^ An air balloon was used instead of O_2_. ^8^ An average value of three runs (91%, 90%, and 88%). ^9^ Result with **1a** (0.50 mmol) in MeOH (2.0 mL). ^10^ Yield of isolated product. ^11^ Result with **1a** (1.0 mmol) in MeOH (1.0 mL), 4 h. ^1^^2^ Ethanol (1.0 mL) was used instead of methanol. ^13^ Yield of **2a’**. ^1^^4^ Result with 0.40 mol% of catalyst.

**Table 2:**
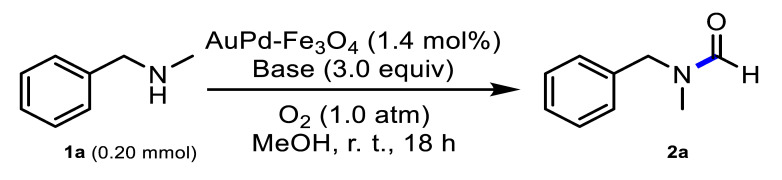
Base screening of the *N*-formylation of amine ^1^.

Entry	Base	Yield ^2^	Entry	Base	Yield ^2^
1	-	14	6	K_2_CO_3_	54
2	LiOH·H_2_O	60	7	Cs_2_CO_3_	69
3	NaOH	65	8	KO*t* Bu	69
4	KOH	75	9	K_3_PO_4_	60
5	CsOH·H_2_O	91	10	CsF	39

^1^ Reaction conditions: **1a** (0.20 mmol), AuPd–Fe_3_O_4_ (1.4 mol%), base (3.0 equiv), O_2_ (1.0 atm), methanol (1.0 mL), r. t., 18 h. ^2^ Determined from ^1^H NMR spectral analysis through the use of mesitylene as an internal standard.

**Table 3:**
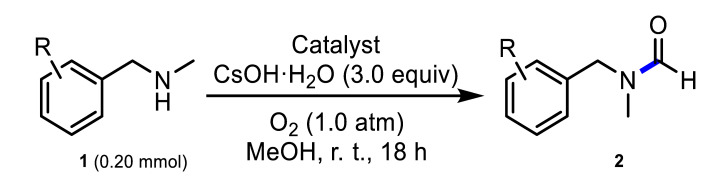
Comparison on the Effect of Alloy Bimetallic Catalyst and Combination of Two Metal Catalysts ^1^.

Entry	Catalyst ^3^	Product Yield with Various R Group (%) ^2^				
		H	4-Me	4-OMe	3-NO_2_	3-CN
1 ^4^	Au	51	61	27	25	23
2 ^5^	Pd	57	28	18	8	5
3 ^6^	Au + Pd	90	62	34	26	32
4 ^7^	AuPd	91	69	83	73	59

^1^ Reaction conditions: **1** (0.20 mmol), catalyst, CsOH·H_2_O (3.0 equiv), O_2_ (1.0 atm), methanol (1.0 mL), r. t., 18 h. ^2^ Yields were determined from ^1^H NMR spectral analysis through the use of mesitylene as an internal standard. ^3^ Catalyst composition: Au–Fe_3_O_4_ (5.47 wt% Au), Pd–Fe_3_O_4_ (8.20 wt% Pd), AuPd–Fe_3_O_4_ (11.7 wt% Au, 6.23 wt% Pd, Au:Pd = 1:0.99). ^4^ Au–Fe_3_O_4_ (2.8 mol%) used. ^5^ Pd–Fe_3_O_4_ (2.8 mol%) used. ^6^ Au–Fe_3_O_4_ (1.4 mol%), Pd–Fe_3_O_4_ (1.4 mol%) used. ^7^ AuPd–Fe_3_O_4_ (1.4 mol%) used.

**Table 4:**
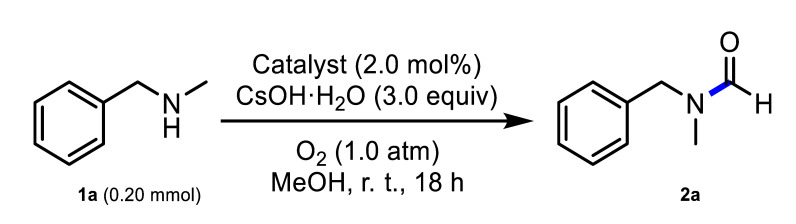
Reactivity comparison of various AuPd catalysts ^1^.

Entry	Catalyst	Yield ^2^
1	Au_1_Pd_0.23_–Fe_3_O_4_	76
2	Au_1_Pd_0.39_–Fe_3_O_4_	76
3	Au_1_Pd_1.08_–Fe_3_O_4_	91
4	Au_0.65_Pd_1_–Fe_3_O_4_	79
5	Au_0.39_Pd_1_–Fe_3_O_4_	69

^1^ Reaction conditions: **1a** (0.20 mmol), catalyst (2.0 mol%), CsOH·H_2_O (3.0 equiv), O_2_ (1.0 atm), methanol (1.0 mL), r. t., 18 h. ^2^ Determined from ^1^H NMR spectral analysis through the use of mesitylene as an internal standard.

## Data Availability

Data are contained within the article or Supplementary Materials.

## Supplementary Materials

The following are available online at https://www.mdpi.com/article/10.3390/nano11082101/s1, Table S1: Catalyst screening, Table S2. Base screening, Table S3. Catalyst loading, Figure S1. SEM analysis, Figure S2. SEM-EDS analysis, Figure S3. EDS map spectrum, Figure S4. HR-TEM analysis, Figure S5. BF-STEM and HADDF-STEM analysis, Figure S6. Particle distribution of AuPd–NPs, Figure S7. XPS analysis, Figure S8. ICP-AES analysis, Figure S9. XRD analysis, Figure S10. SEM, EDS, and ICP-AES analysis of Au_x_Pd_y_–Fe_3_O_4_, Figure S11. SEM analysis of recycled catalyst, Figure S12. HR-TEM analysis of recycled catalyst, Figure S13. SEM analysis of other AuPd–NPs, Table S4. ICP-AES of other AuPd–NPs, Table S5. Formylation data with other AuPd–NPs, Figure S14. Kinetic data of *N*-formylation, Table S6. Substrate scope under high substrate concentration, Figure S15. Determination of the methanol oxidation products, Figure S16. Initial kinetics of HCHO-DNPH generation, Table S7. Control experiments.
