# Supplementary material for: Oxidative N-Formylation of Secondary Amines Catalyzed by Reusable Bimetallic AuPd–Fe3O4 Nanoparticles
Source: Nanomaterials (Basel). 2021 Aug 18;11(8):2101. doi: 10.3390/nano11082101 (PMC8400704; doi:10.3390/nano11082101)
Supplement: Supplementary file 1 [file nanomaterials-11-02101-s001.zip › nanomaterials-1316044-supplementary.pdf]

Supporting Information

Oxidative *N*-Formylation of Secondary Amines Catalyzed by Reusable Bimetallic AuPd–Fe_3O_4 Nanoparticles

Sabyuk Yang, Ahra Cho [†], Jin Hee Cho [†] and Byeong Moon Kim *

Department of Chemistry, College of Natural Sciences, Seoul National University, Seoul 08826, Korea;
diablopkpk@snu.ac.kr (S.Y.); araelliecho@snu.ac.kr (A.C.); 201722658@snu.ac.kr (J.H.C.)
***** Correspondence: kimbm@snu.ac.kr
† These authors contributed equally to this work.

Table of Contents

I. Experimental section

1. General information……………………………………………………….S3
2. Experimental procedures of the synthesis of catalysts…………………...S8
3. Supplementary reaction optimization data
· **Table S1.** Catalyst screening…………………………………….……..S10
· **Table S2.** Base screening…………………………………………….....S11
· **Table S3.** Catalyst loading……………………………………………....S12
4. Characterization of catalysts
· **Figure S1.** SEM analysis…………………………………………...…...S13
· **Figure S2.** SEM-EDS analysis…………………………………………....S14
· **Figure S3.** EDS map spectrum...............………..……………………S17
· **Figure S4.** HR-TEM analysis…………………………………………...…S18
· **Figure S5.** BF-STEM and HADDF-STEM analysis……………………...S20
· **Figure S6.** Particle distribution of AuPd–NPs……..………..…………S22
· **Figure S7.** XPS analysis………………………………………………….S24
· **Figure S8.** ICP-AES analysis…………………………………………….S26
· **Figure S9.** XRD analysis…………………………..…...………………S27

5. Supplementary data
· **Figure S10.** SEM, EDS, and ICP-AES analysis of Au_xPd_y–Fe_3O_4……S28
· **Figure S11.** SEM analysis of recycled catalyst……..………………….. S32
· **Figure S12.** HR-TEM analysis of recycled catalyst……………………..S33
· **Figure S13.** SEM analysis of other AuPd–NPs.....………..…………..S34
· **Table S4.** ICP-AES of other AuPd–NPs.……………….……...……..S34
· **Table S5.** Formylation data with other AuPd–NPs……………………...S35
· **Figure S14.** Kinetic data of *N*-formylation......……………………........S35
· **Table S6.** Substrate scope under high substrate concentration............S36
· **Figure S15.** Determination of the methanol oxidation products…..…..S37
· **Figure S16.** Initial kinetics of methanol oxidation to HCHO as measured from HCHO-DNPH generation...S38
· **Table S7.** Control experiments……………………………….........S39
6. Characterization of products…..……………………..……………......S40

II. References…..…………………………………………………………..S48

III. NMR spectra…..……………………………………………………...S49

I. Experimental section

1. General information

All 1D NMR spectroscopy experiments were conducted with a DD2MR400 (Agilent Technologies, Santa Clara, CA, USA) for 400 MHz, Varian 500 (Varian, Inc., Palo Alto, CA, USA) for 500 MHz. NMR spectra were processed with MestReNova. Chemical shifts are reported in ppm and referenced to residual solvent peaks ($CHCl_3$ in $CDCl_3$: 7.26 ppm for ^1H, 77 ppm for ^{13}C). Coupling constants are reported in Hertz. Secondary amine substrates (**1b**, **1d**, **1e**, **1f**, **1i**, **1k**, **1l**, **1m**, **1q**) were synthesized by known procedures.[1–3] Rest of the starting materials and reagents were purchased from Acros Organics (Pittsburgh, PA, USA), Sigma-Aldrich Aldrich (St. Louis, MO, USA), Alfa Aesar (Ward Hill, MA, USA), Tokyo Chemical Industry (Tokyo, Japan), and used without further purification.

Catalyst characterization

SEM images were obtained using JSM-7800F Prime (JEOL Ltd., Tokyo, Japan). HR-TEM images were obtained using JEM-3010 (JEOL Ltd., Tokyo, Japan). STEM images were obtained using JEM-ARM200F (JEOL Ltd., Tokyo, Japan). XPS data were obtained using AXIS SUPRA (Kratos Analytical Ltd., Manchester, UK). ICP-AES data were obtained using OPTIMA 8300 (Perkin-Elmer, Waltham, MA, USA). The machines mentioned above are installed at the National Center for Inter-University Research Facilities (NCIRF) at Seoul National University.

The powder X-ray diffraction (XRD) was performed using a D8 Advance (Bruker, Billerica, MA, USA) installed the National Instrumentation Center for Environmental Management (NICEM) at Seoul National University.

Materials/Instrumentation

ESCA (Electron Spectroscopy for Chemical Analysis)

1. Model: AXIS SUPRA (Kratos Analytical Ltd., Manchester, UK)
2. Vacuum System
(1) The sample analysis chamber is a multiport ultra-high vacuum chamber of mu-metal construction.
(2) Bakeout: 24 hours~7 day timer with thermostatically controlled, no need remove any cables and cameras before baking.
(3) Pumping kit
 - The sample analysis chamber: TMP, TSP
 (Base pressure in the analysis chamber is < $5x10^{-10}$ torr)
 - The load lock chamber: TMP, oil free dry scroll pump.
 (Base pressure in the analysis chamber is < $5x10^{-8}$ torr)

3. Electron energy analyzer
(1) 165 mm mean radius concentric hemispherical analyzer for spectroscopy and a spherical mirror analyzer for imaging.
(2) 128 channel delay line detector (DLD) be used for both spectroscopy and parallel imaging modes.
(3) In spectroscopy mode should have greater than 100 discrete data channels improving the sensitivity in spectroscopy mode.
(4) X-ray photoelectron spectroscopy
 - Ultimate energy resolution: ≤ 0.48 eV ($Ag3d_{5/2}$ peak)
(5) X-ray photoelectron imaging
 - Lateral resolution of the parallel imaging: ≤1 μm

4. Automated monochromatic X-ray source
(1) 500 mm Rowland circle geometry, controlled by data system.
(2) Auto arrangement provides easy control, optimization and calibration of the mirror

position and ensures that the X-ray illuminated area is correctly aligned with the analysis position.

5. Sample alignment system

(1) Load lock chamber camera gives a global view of each sample holder such that analysis positions can be viewed, indexed and pre-programmed ready for data acquisition.

(2) High magnification sample analysis chamber microscope has a motorized, more than 12 x optical zoom controlled from the data-system facilitating remote operation of the camera.

(3) The optical images are captured within the Data Acquisition software and should be saved and manipulated in the Data Processing software.

6. Sample manipulator and platen

(1) Automated five axis X, Y, Z, ϴ, Φ precision manipulator system

X-axis; 150 mm

Y-axis; ±15 mm

Z-axis; -12~+16 mm

θ-rotation; ±90°

Φ-rotation; ±180°

(2) The sample manipulator have standard reference materials (Au, Ag and Cu) for spectroscopic calibration as well as an Au grid for imaging calibration permanently mounted.

(3) sample holder: Dual height sample holder, Combination sample holder, Z-rotation sample holder

7. Gas cluster ion source

(1) Monatomic Ar^{+} ion mode: 500 eV ~ 5 keV

(2) Ar^{n+} cluster ion mode: ~ 20 keV

(3) The ion source be fully integrated with the data acquisition system.

8. UPS (Ultraviolet Photoelectron Spectroscopy)

(1) Windowless discharge lamp of fully bakeable metal and ceramic construction

(2) The minimum step size: 1 meV

(3) Sensitivity: 1,000,000 cps at 120 meV resolution

- Ag Fermi edge excited by He I radiation

(4) He I: He II ratio: 4 : 1

9. ISS (Ion Scattering Spectroscopy)

(1) Ion scattering spectroscopy: Ar^+ or He^+ ions.

(2) Energy resolution

Sensitivity: 12,000 cps/nA at 12 eV FWHM

- Au scattering, 1 kV He^+ ions

Transmission Electron Microscope II (ccd camera type)

1. Model: JEM-3010 (JEOL Ltd., Tokyo, Japan)
2. Accelerating Voltage: 80 to 300 Kv
3. Gatan Digital Camera (MSC-794)
4. Resolution: Point image: 0.17 nm

   Lattice image: 0.14 nm
5. MAG: x50 ~ x1,500,000
6. Camera Length: SA DIFF Mode: 120 ~ 3,000 mm

Cs-STEM (Cs corrected STEM with Cold FEG)

1. Model: JEM-ARM200F (JEOL Ltd., Tokyo, Japan)
2. Specifications

   a. HT: 60, 80, 120, 200 kV

   b. Magnification: 50 to 2,000,000 X (TEM), 200 to 1,500,000 X (STEM)

   c. Resolution

- STEM mode: HAADF 0.08 nm/ BF 0.136 nm
- TEM mode: Point 0.23 nm

d. Sample tilting

- X / Y: ±35° / ±30°

3. Analysis functions

a. CCD Camera: OneView camera (25 fps at full 4k x 4k resolution)

b. EDS: SDD Type (Active area 100mm^2/ Solid angle 0.9 sr)

c. EELS: Model 965 GIF Quantum ER

2. Experimental procedures of the synthesis of catalysts

Synthesis of AuPd–Fe_3O_4 NPs

Initially, 177 mg of $PdCl_2$ and 2.00 g of PVP (Mw~10,000 mg mol^{-1}) were placed in 40 mL of ethylene glycol (EG) in a 100 mL round-bottom flask. This mixture was sonicated for 10 min and stirred for 1 h at 100 °C in an oil bath. In a separate 100 mL round-bottom flask, 390 mg of $HAuCl_4{\cdot}3H_2O$ and 1.00 g of PVP were added to 40 mL of water in a 100 mL round-bottom flask. This mixture was sonicated for 10 min and stirred for 30 min at 60 °C in an oil bath. Meanwhile, 1.00 g of Fe_3O_4 NPs were added to 300 mL of water in a two-necked 500 mL round-bottom flask and then sonicated for 10 min. The prepared Pd precursor solution was then injected dropwise onto the Fe_3O_4 suspension with vigorous stirring. After 5 min, the Au precursor solution was added and 100 mg of sodium borohydride in 40 mL of water was injected dropwise using a syringe pump. The resulting mixture was stirred for 5 h at 60 °C. Subsequently, the AuPd alloy on Fe_3O_4 nanoparticles was retrieved via sonication and washing with ethanol (40 mL x 10 times) and dried on a rotary evaporator to give AuPd–Fe_3O_4 NPs (950 mg, 95% yield).

Synthesis of Au–Fe_3O_4 NPs

Initially, 39 mg of $HAuCl_4{\cdot}3H_2O$ and 100 mg of PVP were placed in 4.0 mL of water in a 10 mL round-bottom flask. This solution was sonicated for 1 min and stirred for 30 min at 60 °C. Meanwhile, 100 mg of Fe_3O_4 NPs was added to 30 mL of water in a two-necked 100 mL round-bottom flask. The prepared precursor solution was then injected dropwise to Fe_3O_4 NPs in 30 mL of water, followed by dropwise addition of 10 mg of sodium borohydride in 4.0 mL of water. The mixture was stirred at 60 °C for an additional 5 h. The resultant product was washed with ethanol (40 mL x 10 times) and dried on a rotary evaporator to give Au–Fe_3O_4 NPs (94.8 mg, 95% yield).

Synthesis of Pd–Fe_3O_4 NPs

Initially, 177 mg of $PdCl_2$ and 2.00 g of PVP were placed in 40 mL of EG in a 100 mL round-bottom flask. This mixture was sonicated for 10 min and stirred for 1 h at 100 °C. Meanwhile, 500 mg of Fe_3O_4 NPs was added to 150 mL of EG in a two-necked 500 mL round-bottom flask. The prepared precursor solution was then injected dropwise to Fe_3O_4 NPs in 150 mL of EG, and stirred at 100 °C for an additional 24 h. The resultant product was washed with ethanol (40 mL x 10 times) and dried on a rotary evaporator to give Pd–Fe_3O_4 NPs (440.0 mg, 88% yield).

Synthesis of Au_xPd_y–Fe_3O_4 NPs

For Au_xPd_y–Fe_3O_4 synthesis, the same method used for the synthesis of AuPd–Fe_3O_4 NPs was employed, with different quantities of metals and sodium borohydride. To prepare $Au_1Pd_{0.23}$–Fe_3O_4 NPs, $PdCl_2$ (0.011 g) with PVP (0.125 g), $HAuCl_4{\cdot}3H_2O$ (0.098 g) with PVP (0.250 g), and sodium borohydride (0.025 g) were used. For the synthesis of $Au_1Pd_{0.39}$–Fe_3O_4 NPs, $PdCl_2$ (0.022 g) with PVP (0.250 g), $HAuCl_4{\cdot}3H_2O$ (0.098 g) together with PVP (0.250 g), and sodium borohydride (0.025 g) were used. In the case of $Au_{0.65}Pd_1$–Fe_3O_4 NPs, $PdCl_2$ (0.044 g) with PVP (0.500 g), $HAuCl_4{\cdot}3H_2O$ (0.049 g) with PVP (0.125 g), and sodium borohydride (0.013 g) were used. Finally, for the preparation of $Au_{0.39}Pd_1$–Fe_3O_4 NPs, $PdCl_2$ (0.044 g) with PVP (0.500 g), $HAuCl_4{\cdot}3H_2O$ (0.033 g) with PVP (0.083 g), and sodium borohydride (0.008 g) were used. The resulting mixture was stirred for 5 h at 60 °C. Subsequently, the Au_xPd_y alloy on Fe_3O_4 nanoparticles was retrieved via sonication and washing with ethanol (40 mL x 10 times) and dried on a rotary evaporator to give $Au_1Pd_{0.23}$–Fe_3O_4 NPs (85.6 mg, 86% yield), $Au_1Pd_{0.39}$–Fe_3O_4 NPs (80.6 mg, 81% yield), $Au_{0.65}Pd_1$–Fe_3O_4 NPs (99.9 mg, >99% yield), and $Au_{0.39}Pd_1$–Fe_3O_4 NPs (89.6 mg, 90% yield).

3. Supplementary reaction optimization data

Table S1. Catalyst screening[a]

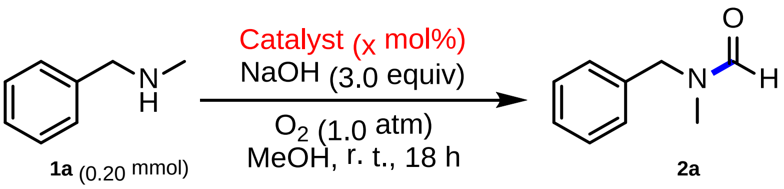

Entry	Catalyst (mol%)	Yield (%)[b]
1	Fe_3O_4 (1.4)	Not detected
2	Au–Fe_3O_4 (2.8)	25
3	Pd–Fe_3O_4 (2.8)	55
4	Au–Fe_3O_4 (1.4) + Pd–Fe_3O_4 (1.4)	91
5	AuPd–Fe_3O_4 (1.4)	65
6	PdPt–Fe_3O_4 (2.0)	21

[a] Reaction conditions: *N*-methyl-1-phenylmethanamine (0.20 mmol), catalyst (x mol%) NaOH (3.0 equiv), methanol (1.0 mL), O_2 (1.0 atm), r. t., 18 h.

[b] Determined from ^1H NMR spectral analysis through the use of mesitylene as an internal standard.

Table S2. Base screening[a]

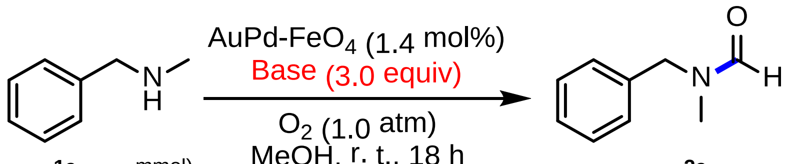

Entry	Base	Yield (%)[b]	Entry	Base	Yield (%)[b]
1	-	14	9	K_2CO_3	54
2	$LiOH \cdot H_2O$	60	10	Cs_2CO_3	69
3	NaOH	65	11	NaO*t*Bu	59
4	KOH	75	12	KO*t*Bu	69
5	$RbOH \cdot xH_2O$	74	13	K_2HPO_4	3
6	$CsOH \cdot H_2O$	91	14	K_3PO_4	60
7	Li_2CO_3	20	15	NaOAc	19
8	Na_2CO_3	29	16	CsF	39

[a] Reaction conditions: *N*-methyl-1-phenylmethanamine (0.20 mmol), AuPd–Fe_3O_4 (1.4 mol%), base (3.0 equiv), methanol (1.0 mL), O_2 (1.0 atm), r. t., 18 h.

[b] Determined from ^1H NMR spectral analysis through the use of mesitylene as an internal standard.

Table S3. Catalyst loading[a]

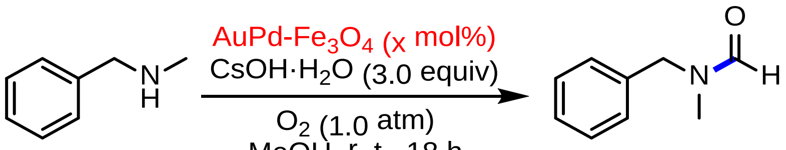

1a (0.20 mmol) **2a**

Entry	Catalyst loading (mol%)	TON[b]	Yield (%)[c]
1	0.4	89	71
2	0.7	50	70
3	1.1	35	78
4	1.4	33	91
5	1.8	24	85
6	2.1	20	83

[a] Reaction conditions: *N*-methyl-1-phenylmethanamine (0.20 mmol), AuPd–Fe_3O_4 (x mol%), CsOH·H_2O (3.0 equiv), methanol (1.0 mL), O_2 (1.0 atm), r. t., 18 h.
[b] Turnover number (TON)=mmol of product/mmol of metal
[c] Determined from ^1H NMR spectral analysis through the use of mesitylene as an internal standard.

3. Characterization of Catalysts

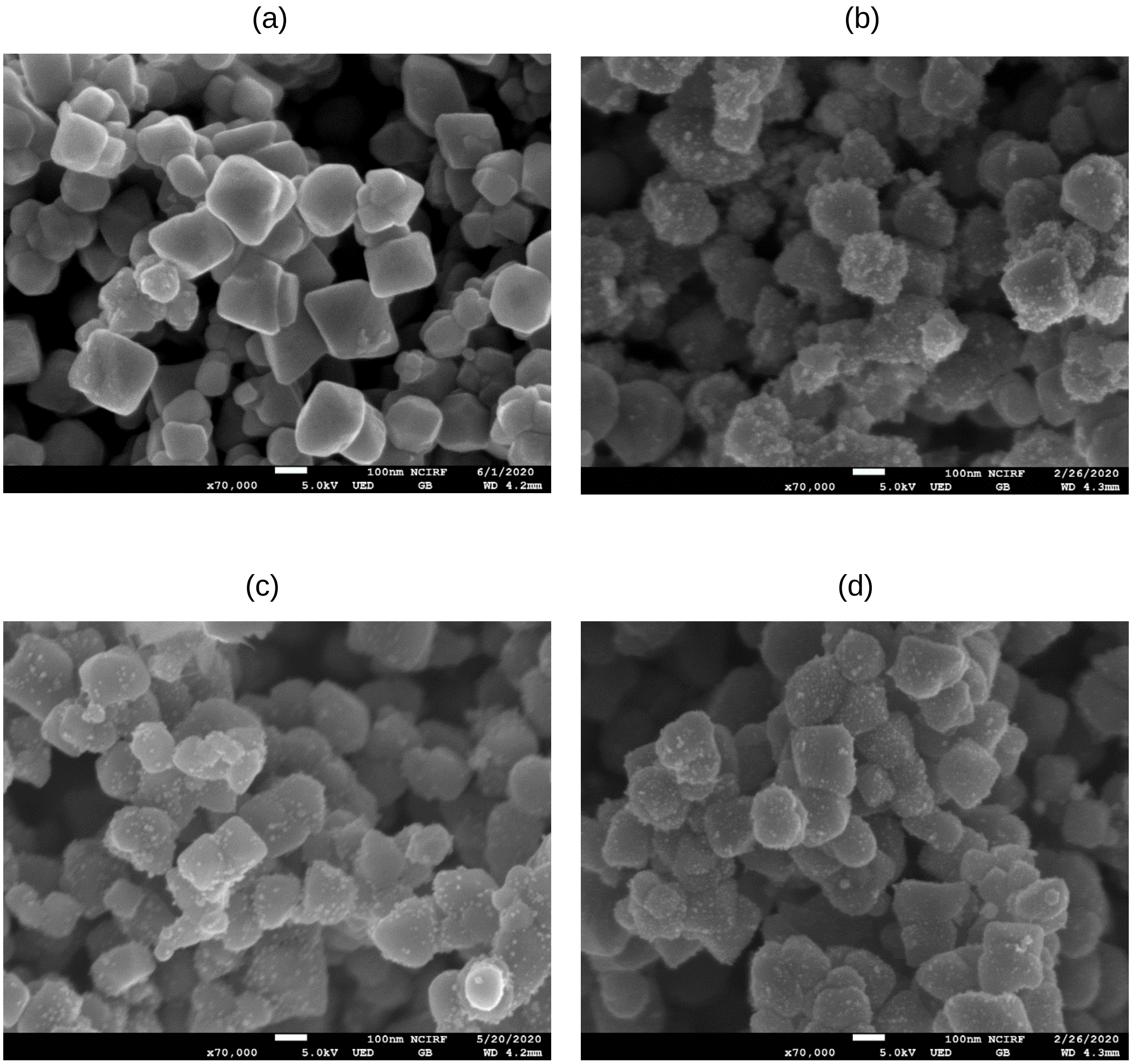

Figure S1. SEM image of (a) Fe_3O_4 NPs, (b) AuPd–Fe_3O_4 NPs, (c) Au–Fe_3O_4 NPs, and (d) Pd–Fe_3O_4 NPs

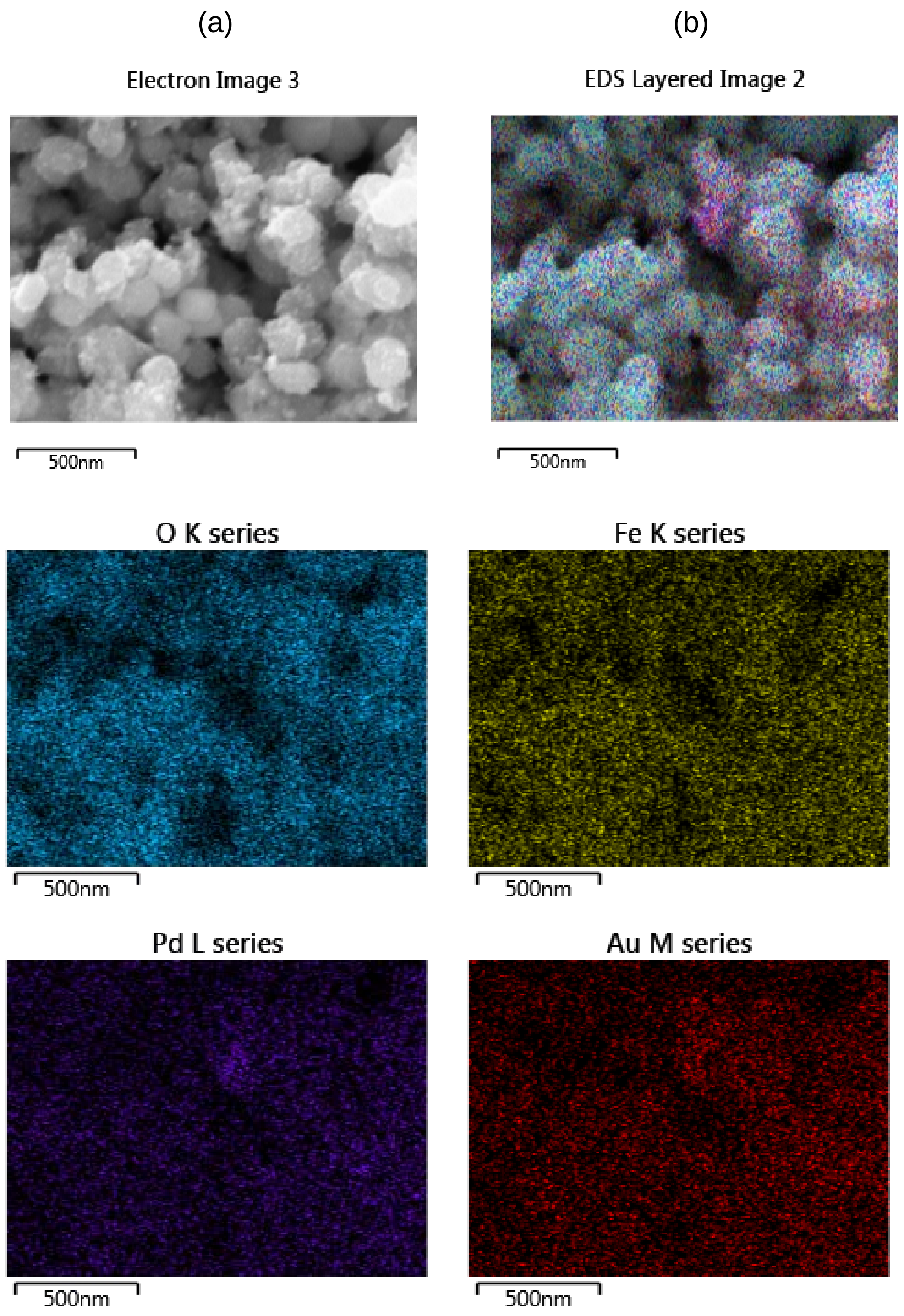

(a)
(b)
Electron Image 3
EDS Layered Image 2
500nm
500nm
O K series
Fe K series
500nm
500nm
Pd L series
Au M series
500nm
500nm

(c) (d)

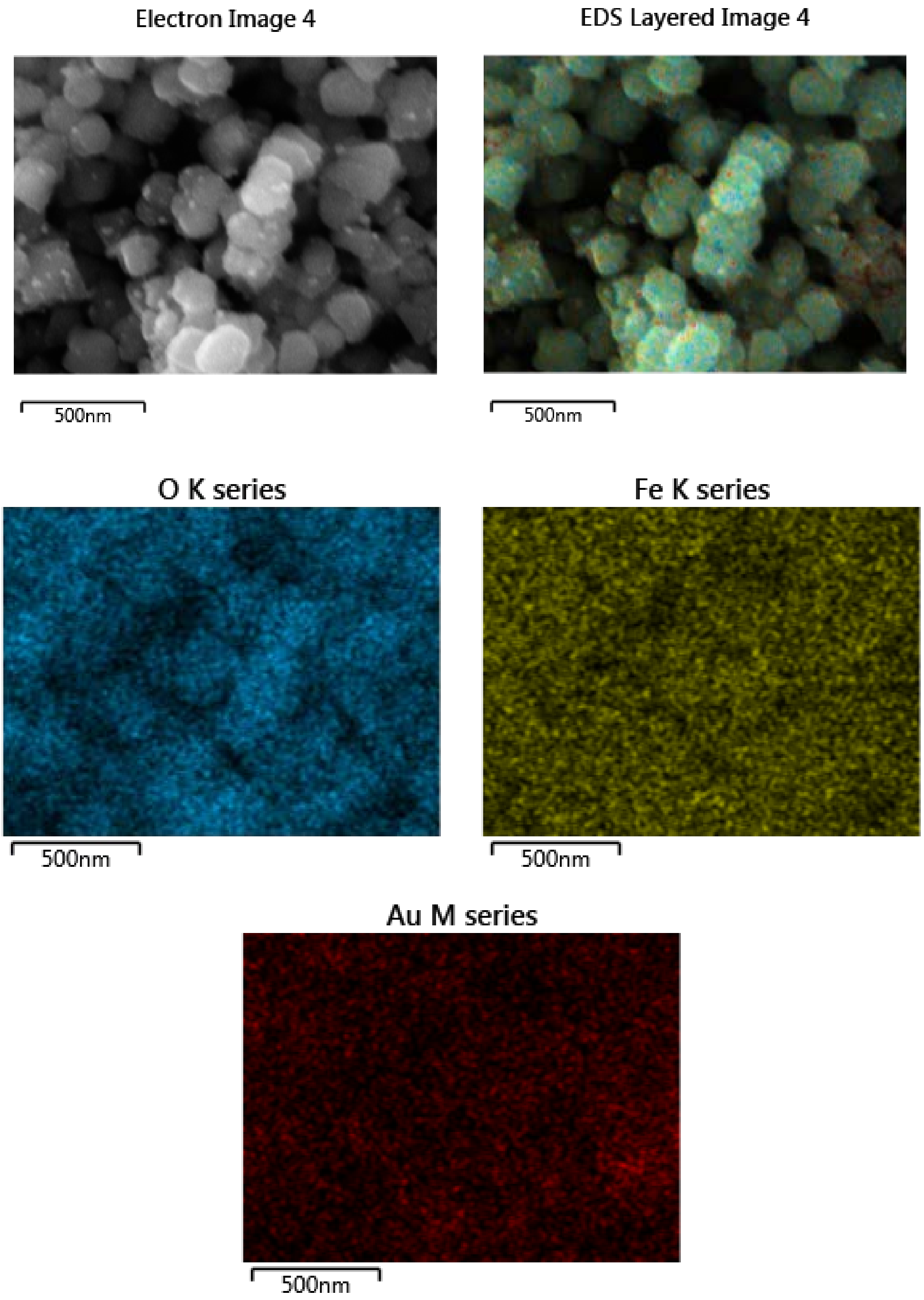
Electron Image 4
EDS Layered Image 4
500nm
500nm
O K series
Fe K series
500nm
500nm
Au M series
500nm

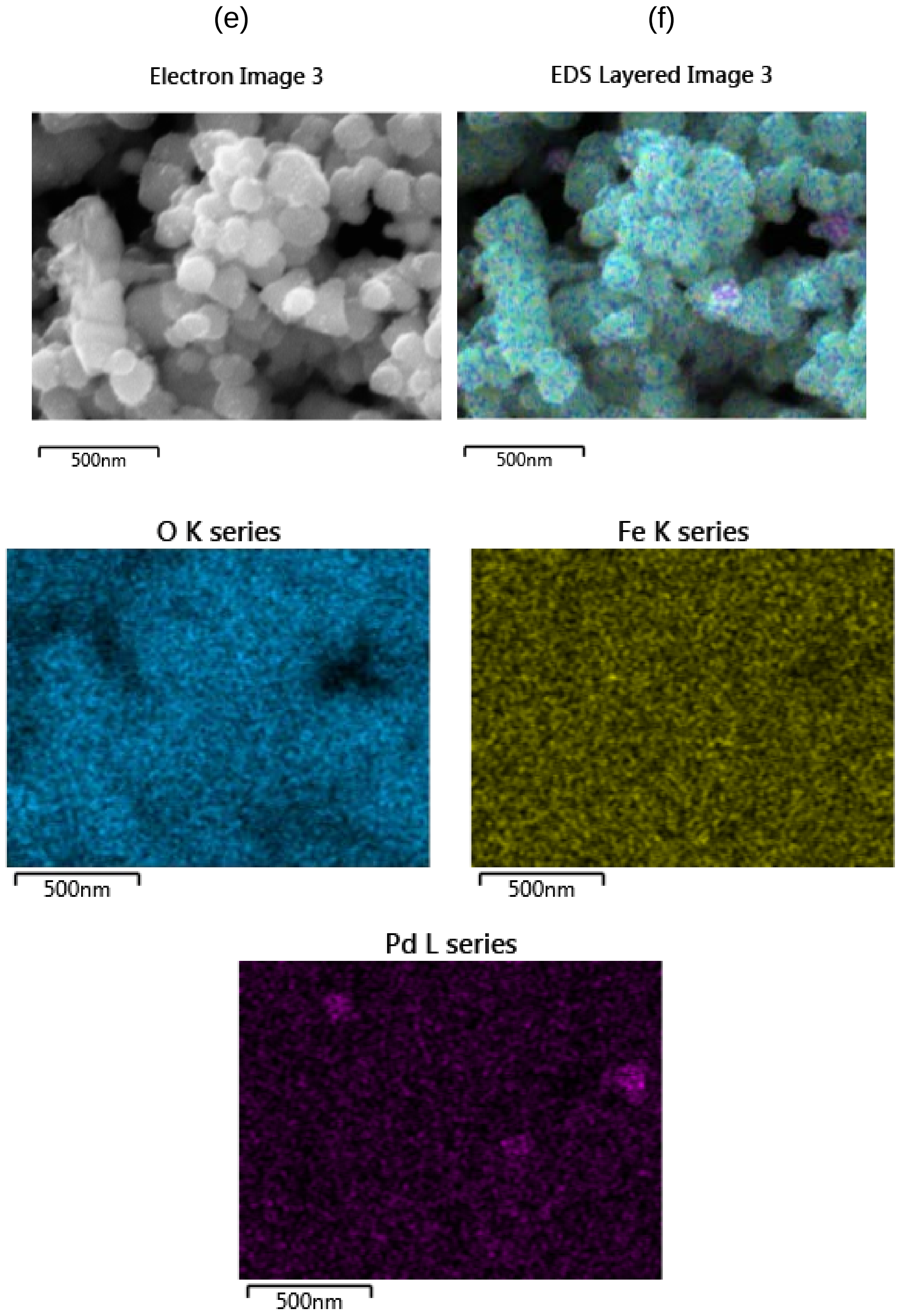

Figure S2. (a) and (b) SEM-EDS images of AuPd–Fe_3O_4 NPs; (c) and (d) SEM-EDS images of Au–Fe_3O_4 NPs; (e) and (f) SEM-EDS images of Pd–Fe_3O_4 NPs

(a)

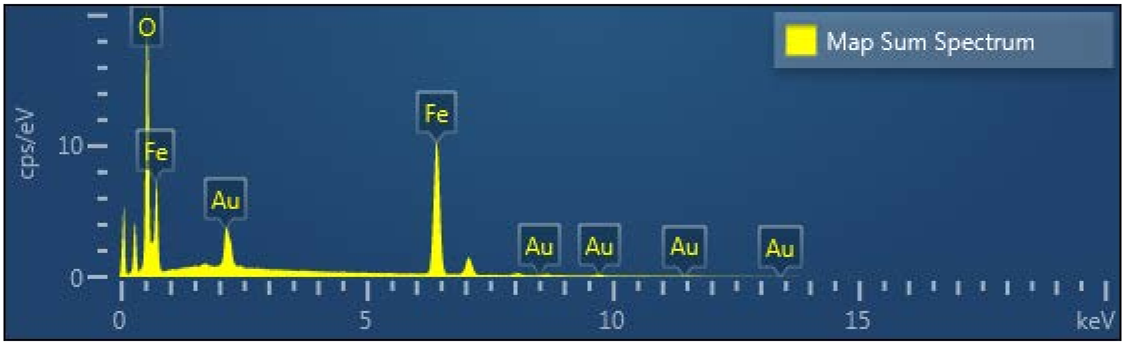

(b)

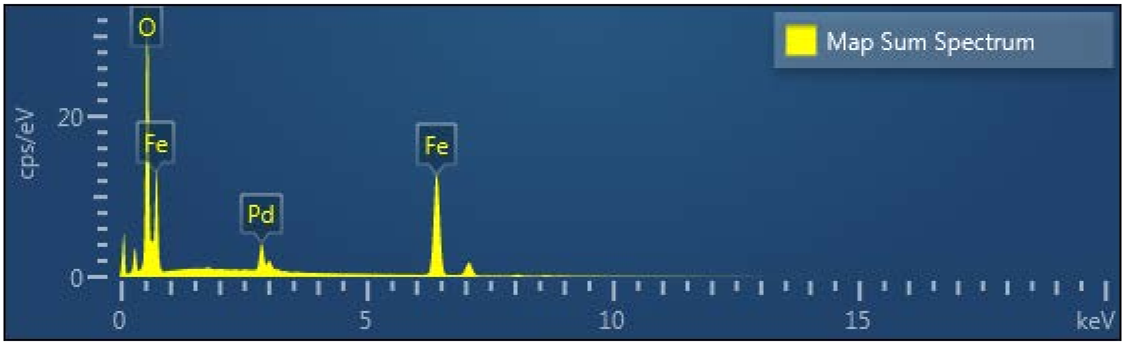

(c)

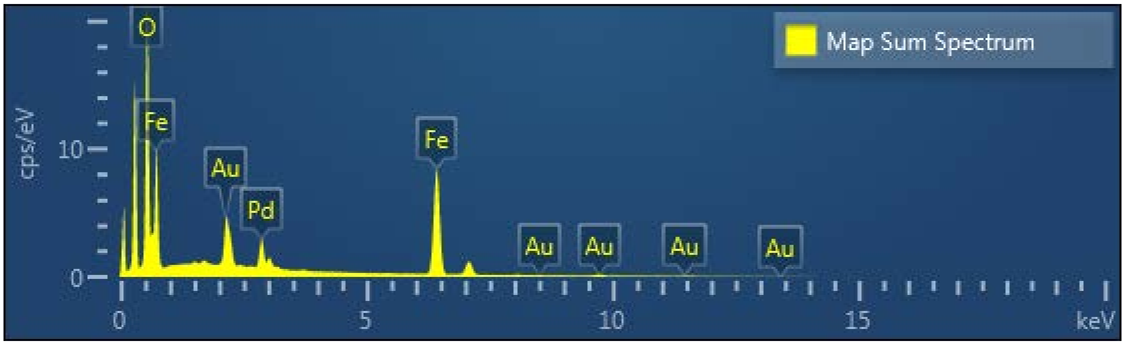

Figure S3. The energy disperse spectroscopy (EDS) map sum spectrum pattern of NPs; (a) Au–Fe_3O_4 NPs, (b) Pd–Fe_3O_4 NPs, and (c) AuPd–Fe_3O_4 NPs

(a)

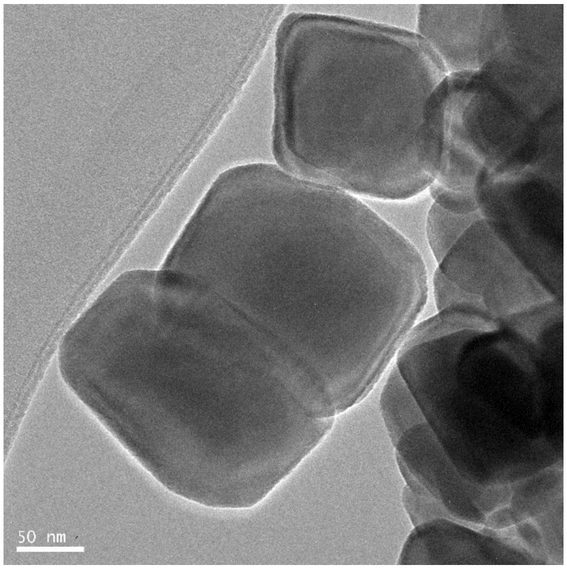

(b)

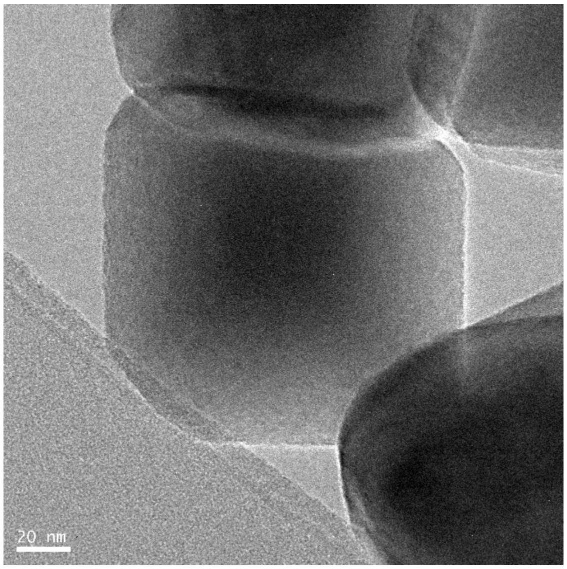

(c)

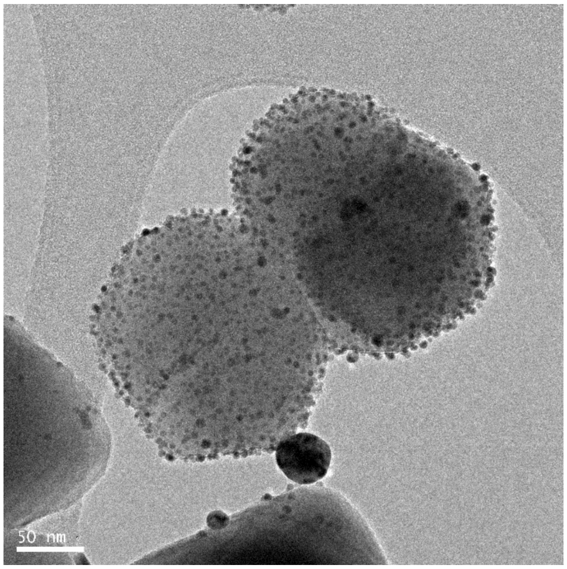

(d)

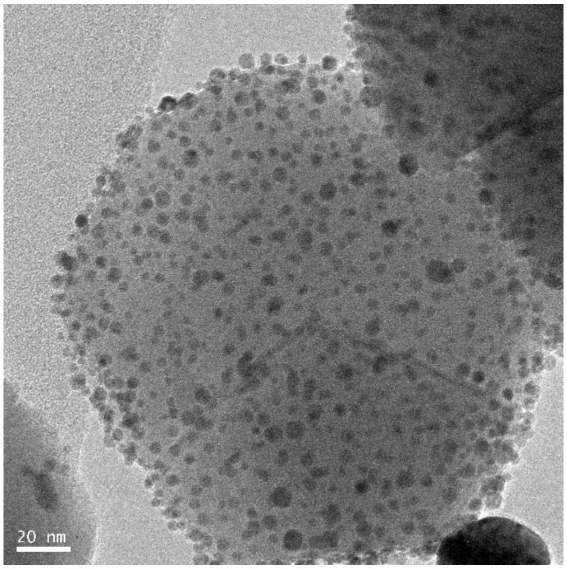

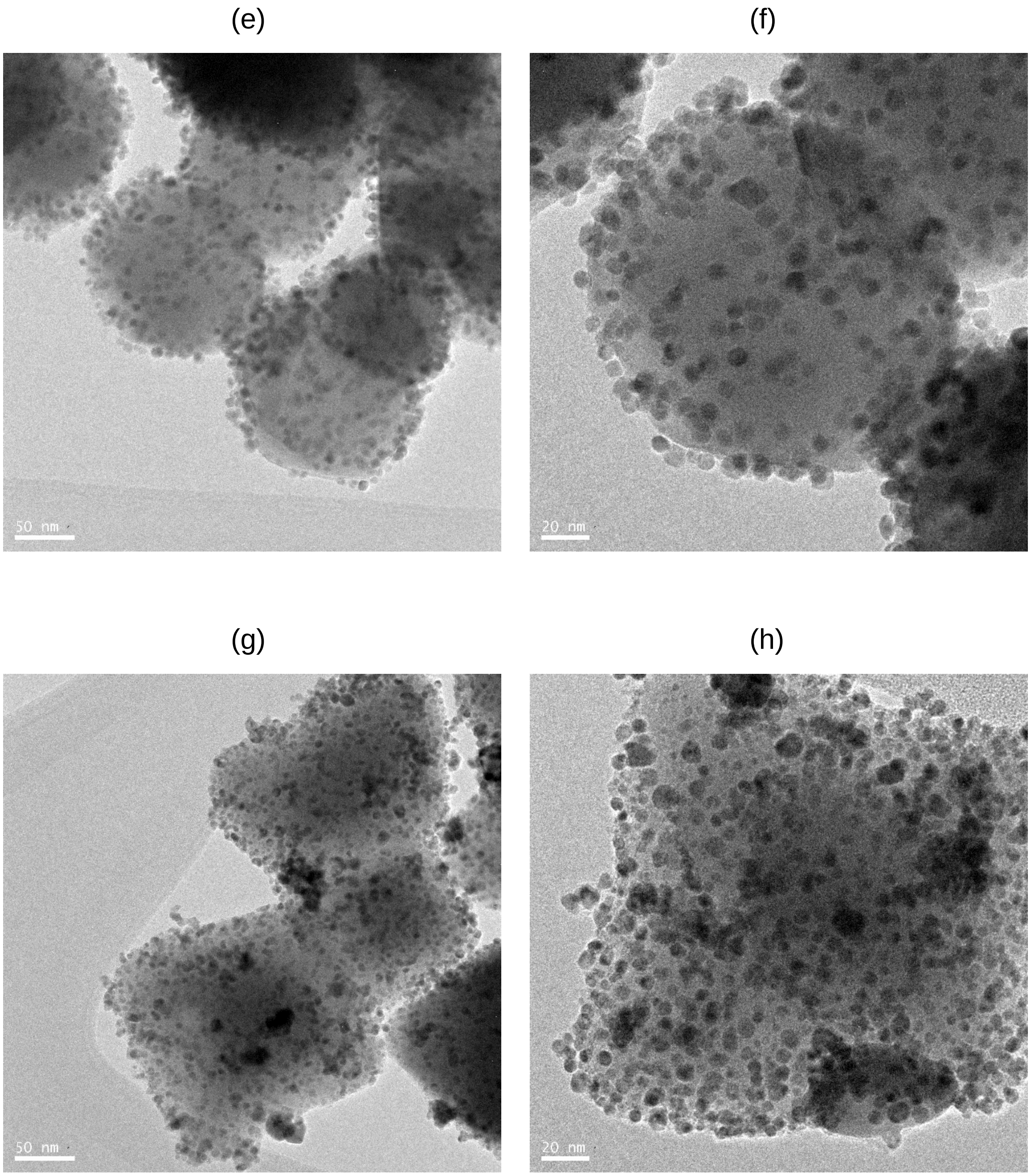

Figure S4. HR-TEM images of (a) and (b) Fe_3O_4 NPs; (c) and (d) Au–Fe_3O_4 NPs; (e) and (f) Pd–Fe_3O_4 NPs; (g) and (h) AuPd–Fe_3O_4 NPs

(a) (b)

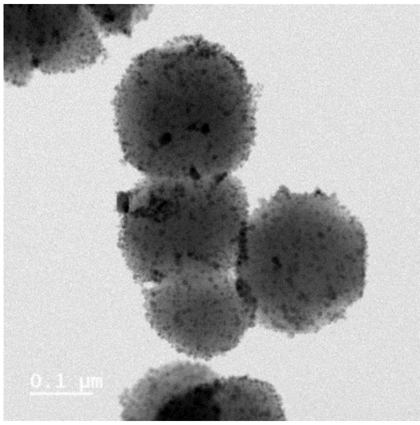

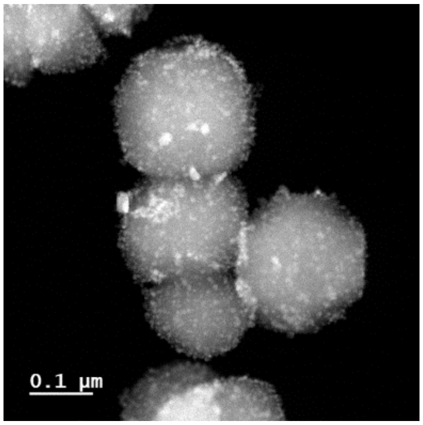

(c) (d)

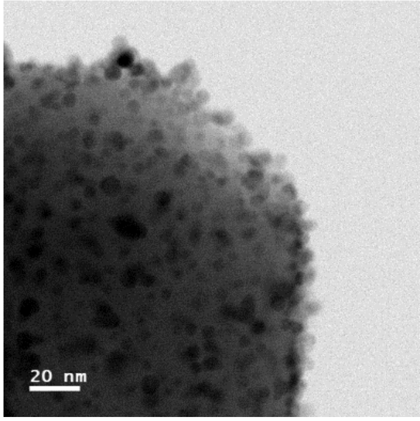

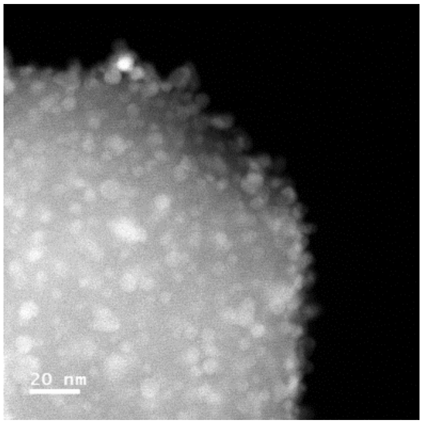

(e) (f)

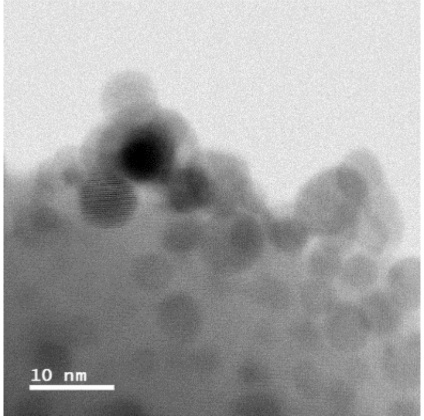

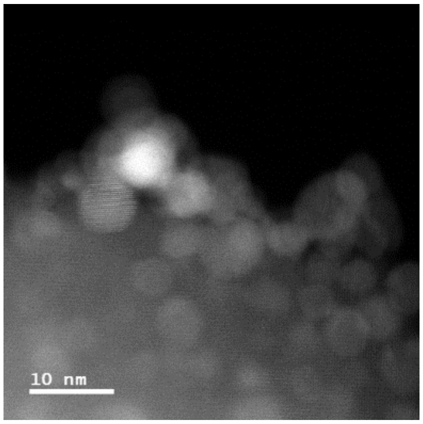

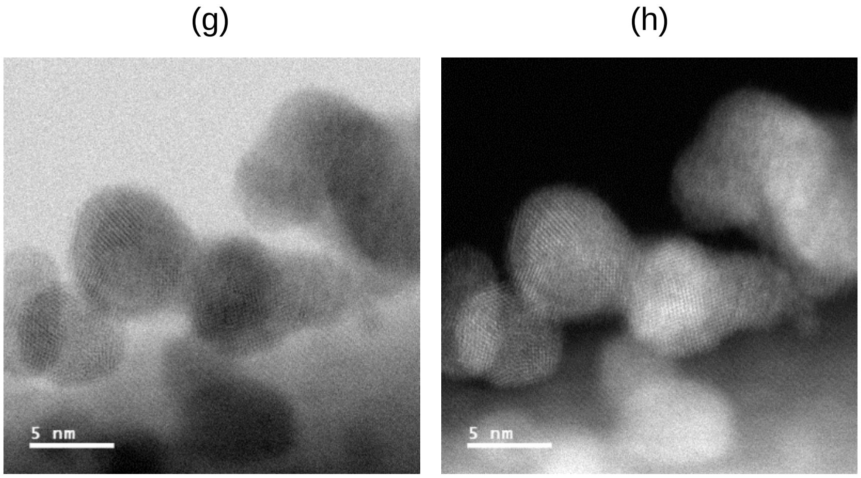

Figure S5. (a), (c), (e), and (g) BF-STEM images of AuPd–Fe_3O_4 NPs; (b), (d), (f), and (h) HADDF-STEM images of AuPd–Fe_3O_4 NPs

(a)

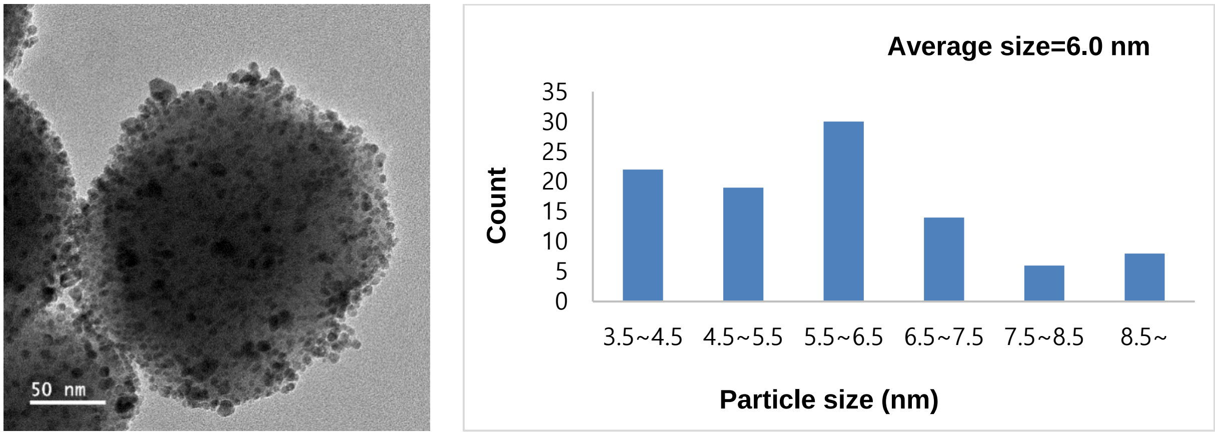
50 nm
Average size=6.0 nm
Count
35
30
25
20
15
10
5
0
3.5~4.5
4.5~5.5
5.5~6.5
6.5~7.5
7.5~8.5
8.5~
Particle size (nm)

(b)

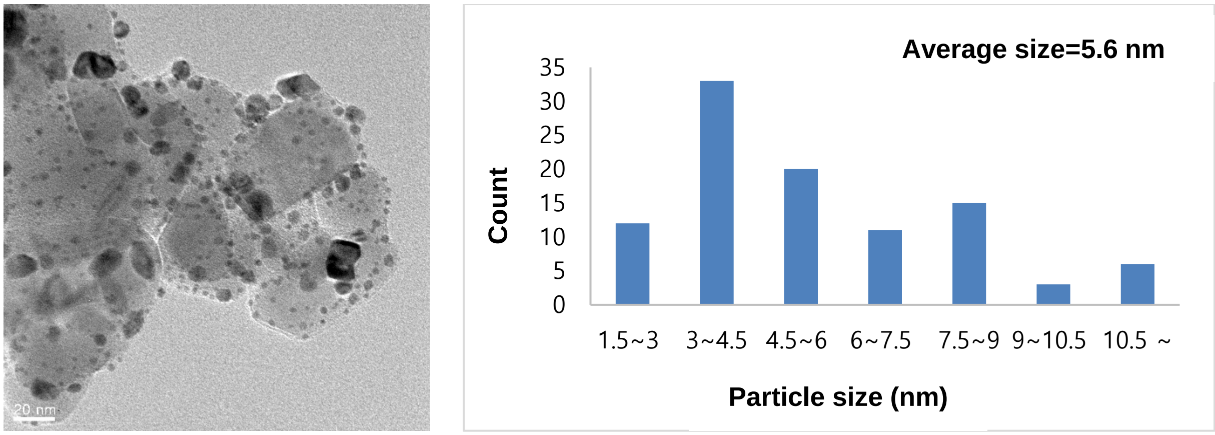
20 nm
Average size=5.6 nm
Count
35
30
25
20
15
10
5
0
1.5~3
3~4.5
4.5~6
6~7.5
7.5~9
9~10.5
10.5 ~
Particle size (nm)

(c)

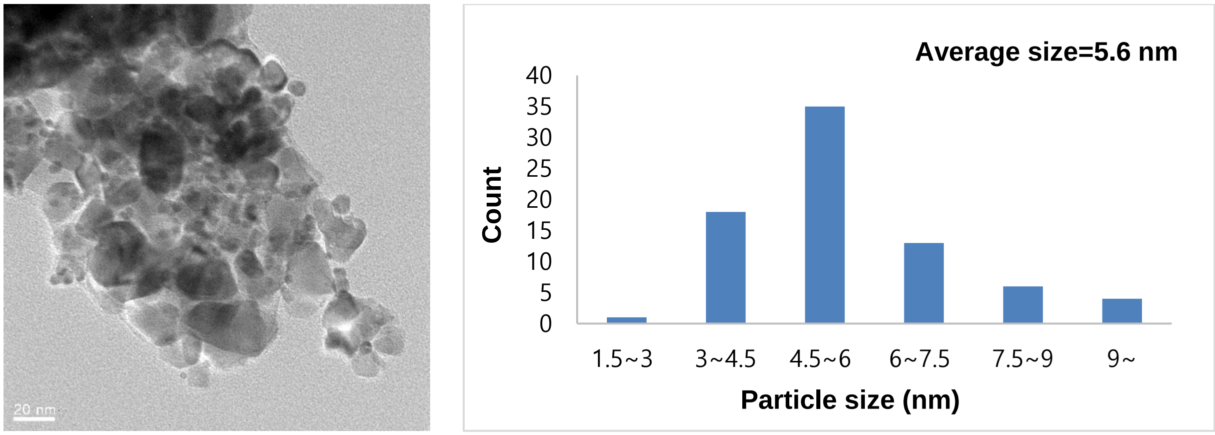
20 nm
Average size=5.6 nm
Count
40
35
30
25
20
15
10
5
0
1.5~3
3~4.5
4.5~6
6~7.5
7.5~9
9~
Particle size (nm)

(d)

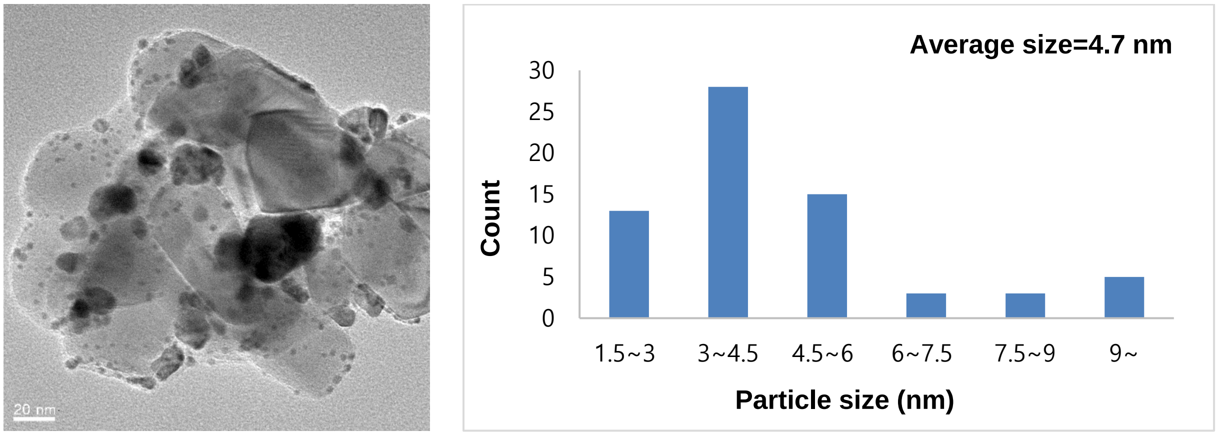

Figure S6. HR-TEM images and particle distribution of (a) AuPd–Fe_3O_4 NPs, (b) AuPd–TiO_2 NPs, (c) AuPd–CeO_2 NPs, and (d) AuPd–C NPs

(a)

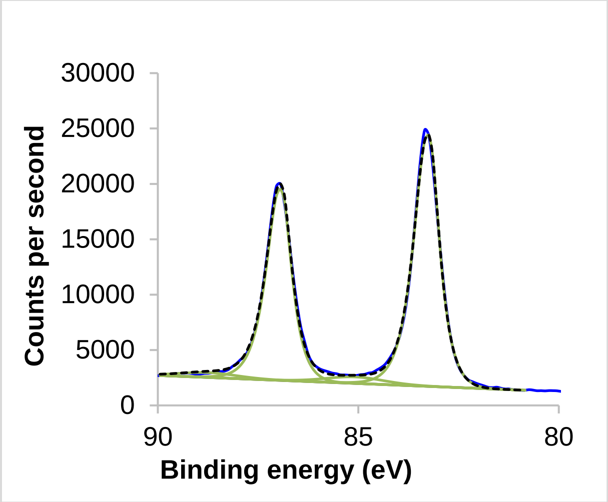
Counts per second
30000
25000
20000
15000
10000
5000
0
90
85
80
Binding energy (eV)

(b)

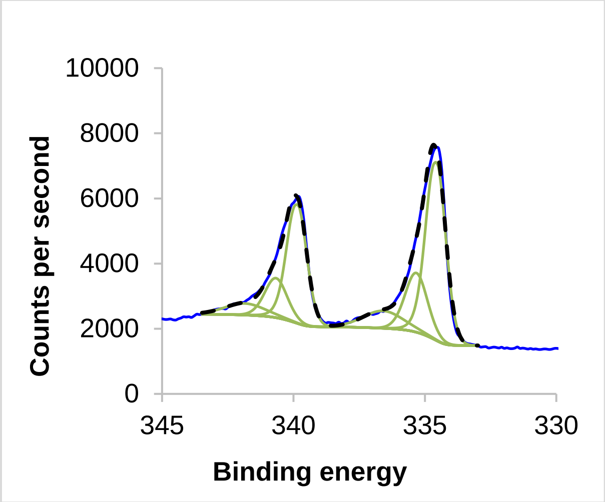
Counts per second
10000
8000
6000
4000
2000
0
345
340
335
330
Binding energy

(c)

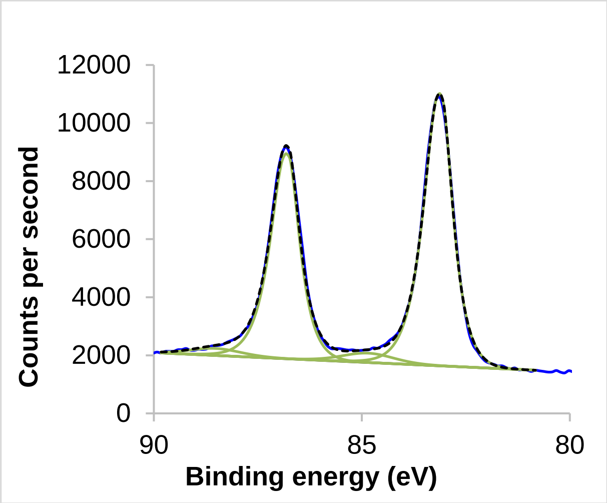
Counts per second
12000
10000
8000
6000
4000
2000
0
90
85
80
Binding energy (eV)

(d)

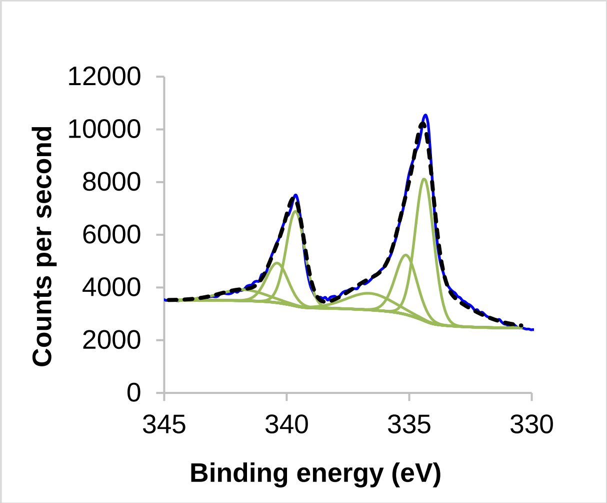
Counts per second
12000
10000
8000
6000
4000
2000
0
345
340
335
330
Binding energy (eV)

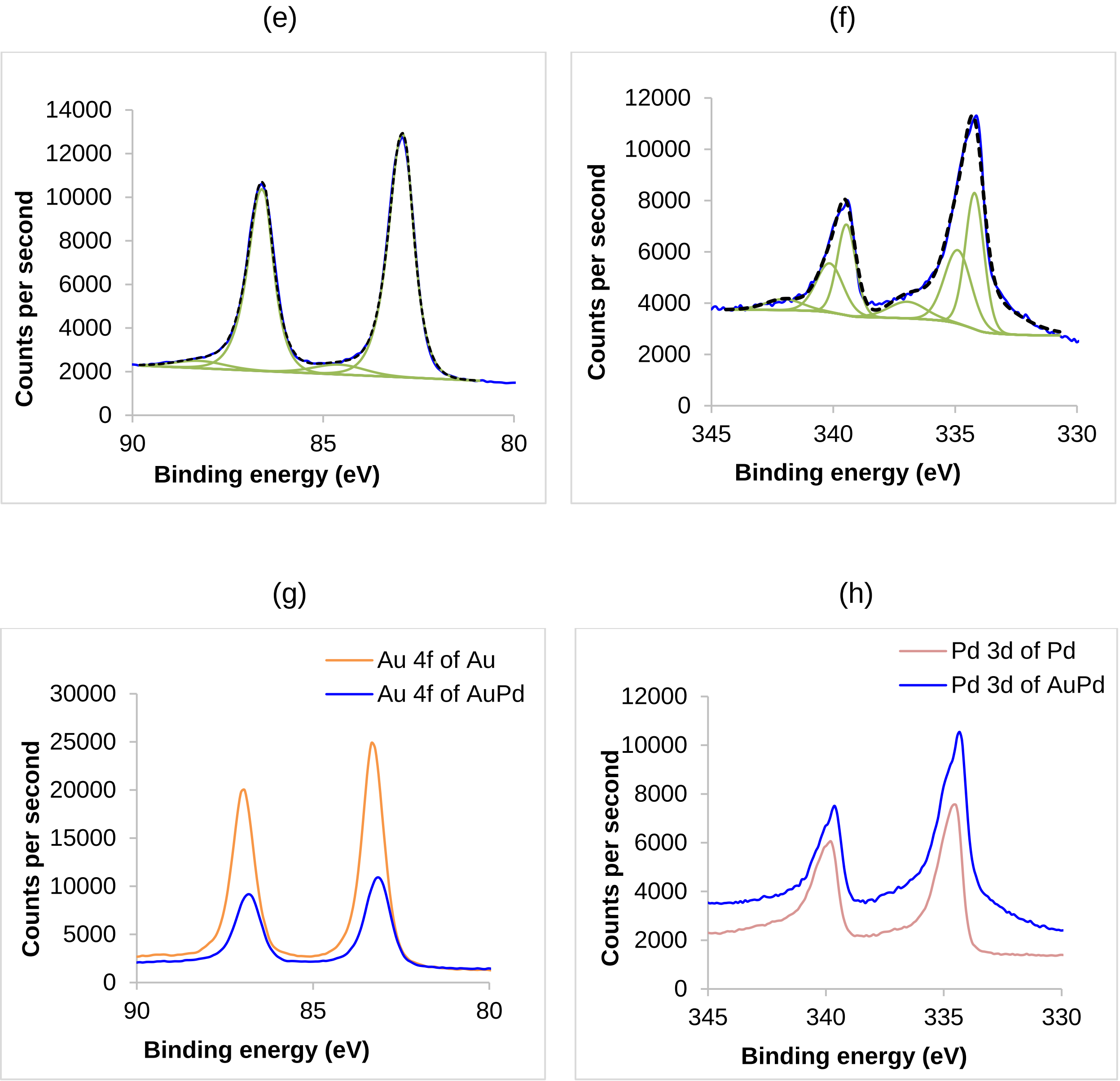

Figure S7. XPS data (a) Au 4f spectra of Au–Fe_3O_4 NPs; (b) Pd 3d spectra of Pd–Fe_3O_4 NPs; (c) Au 4f spectra of fresh AuPd–Fe_3O_4 NPs; (d) Pd 3d spectra of fresh AuPd–Fe_3O_4 NPs; (e) Au 3d spectra of AuPd–Fe_3O_4 NPs after 1 cycle of the catalytic reaction; (f) Pd 3d spectra of AuPd–Fe_3O_4 NPs after 1 cycle of the catalytic reaction; (g) Au 4f spectra of AuPd–Fe_3O_4 NPs and Au–Fe_3O_4 NPs (h) Pd 3d spectra of AuPd–Fe_3O_4 NPs and Pd–Fe_3O_4 NPs

Catalyst	Au (wt%)	Pd (wt%)
AuPd–Fe_3O_4	8.91	5.19
Au–Fe_3O_4	10.5	-
Pd–Fe_3O_4	-	7.52
AuPd–Fe_3O_4 (after 1st reaction)	8.00	4.18
AuPd–Fe_3O_4 (after 10th reaction)	4.35	1.89

Figure S8. ICP-AES data of AuPd–Fe_3O_4 NPs, Au–Fe_3O_4 NPs, Pd–Fe_3O_4 NPs, AuPd–Fe_3O_4 NPs after the first reaction, and AuPd–Fe_3O_4 NPs after the 10th reaction

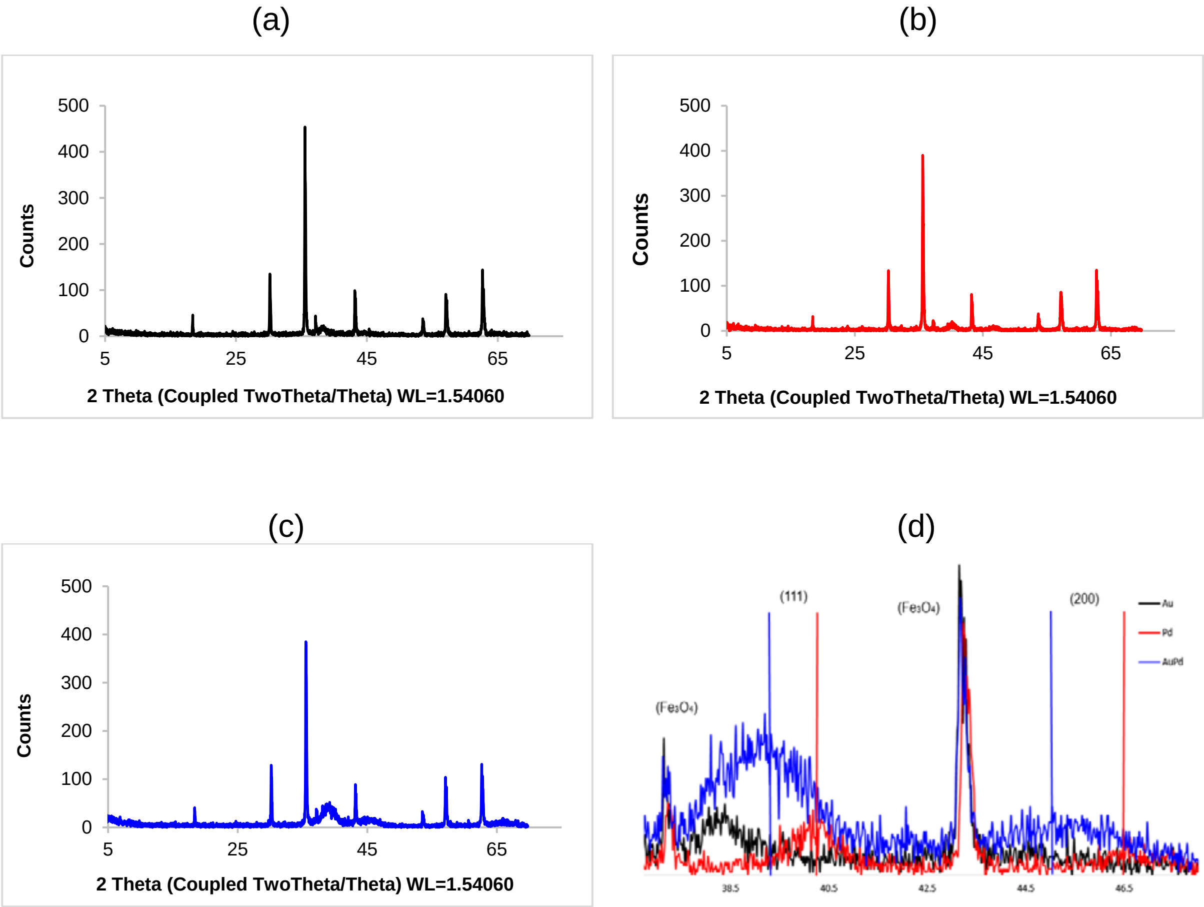

Figure S9. XRD data of (a) Au–Fe_3O_4 NPs, (b) Pd–Fe_3O_4 NPs, (c) AuPd–Fe_3O_4 NPs, and (d) Comparison of XRD peaks of the catalysts

3. Supplementary data

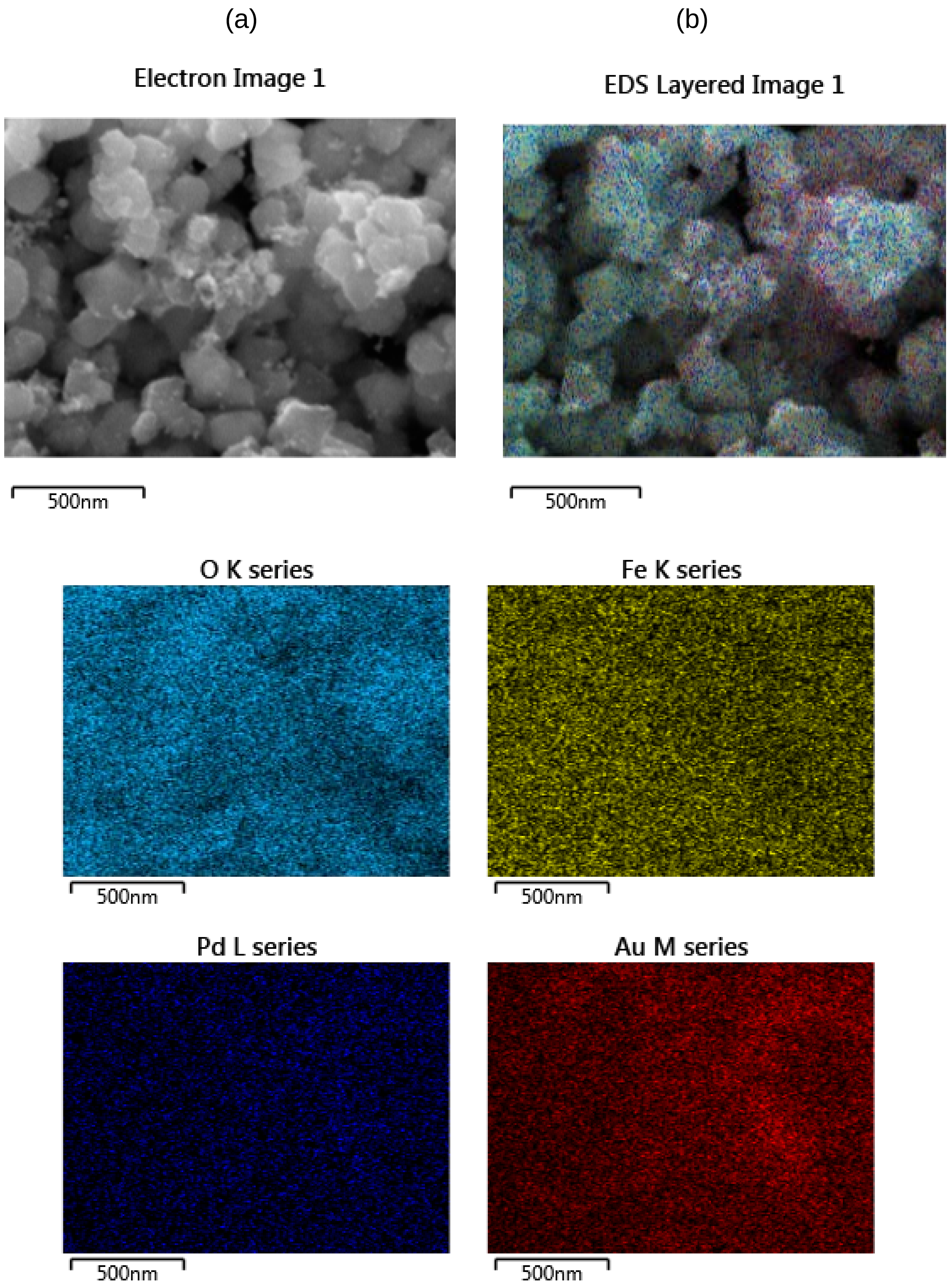

(c)

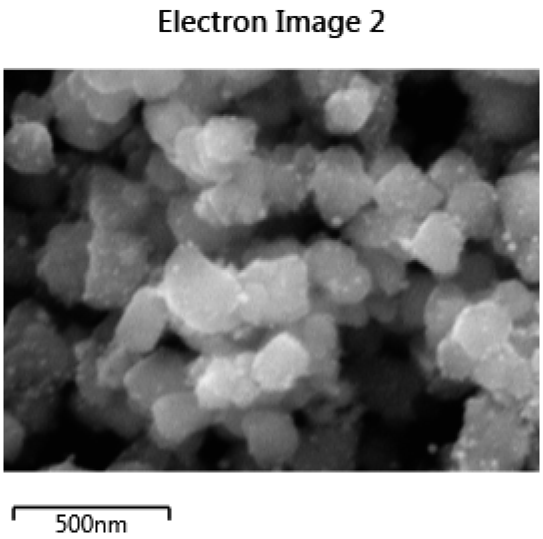
Electron Image 2
500nm

(d)

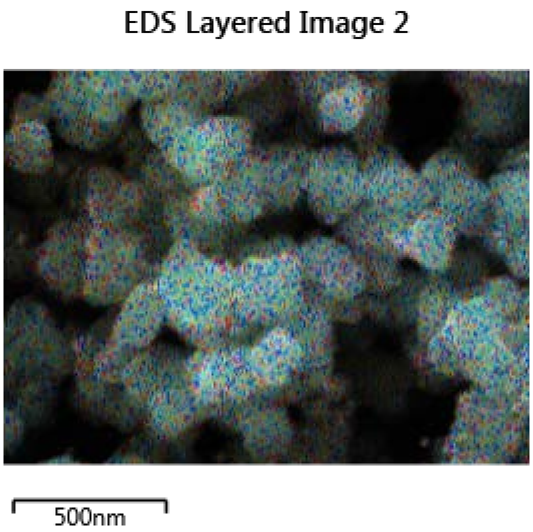
EDS Layered Image 2
500nm

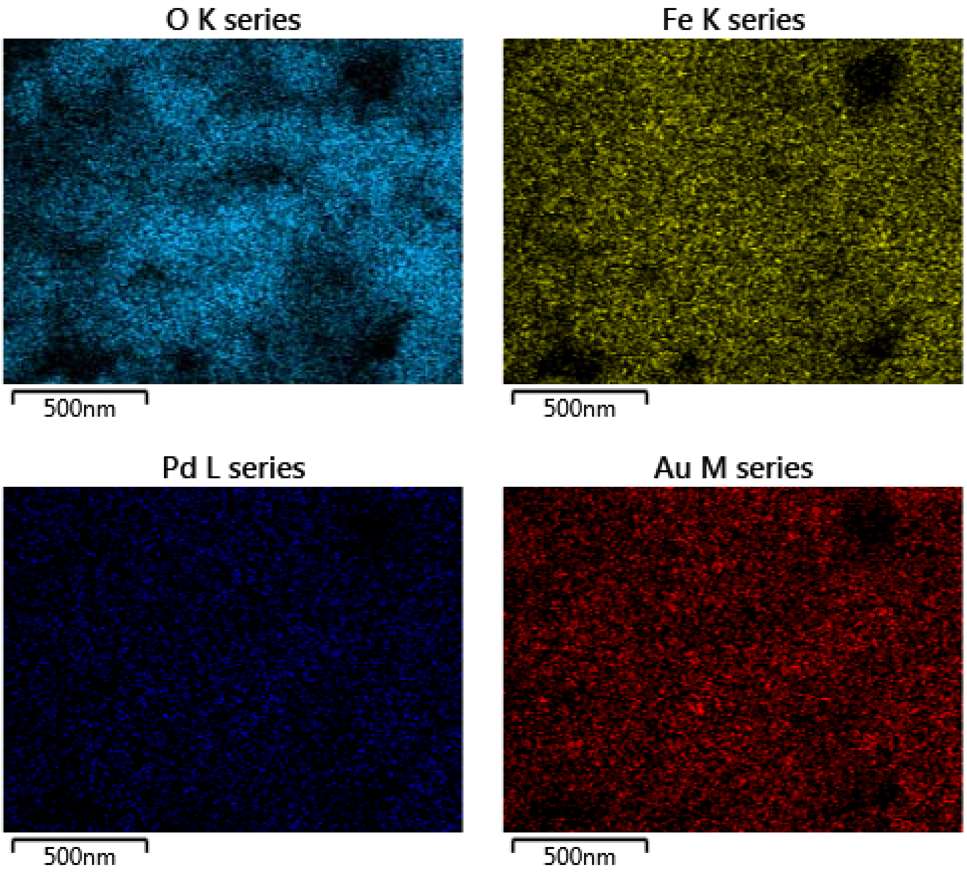
O K series
500nm
Fe K series
500nm
Pd L series
500nm
Au M series
500nm

(e)

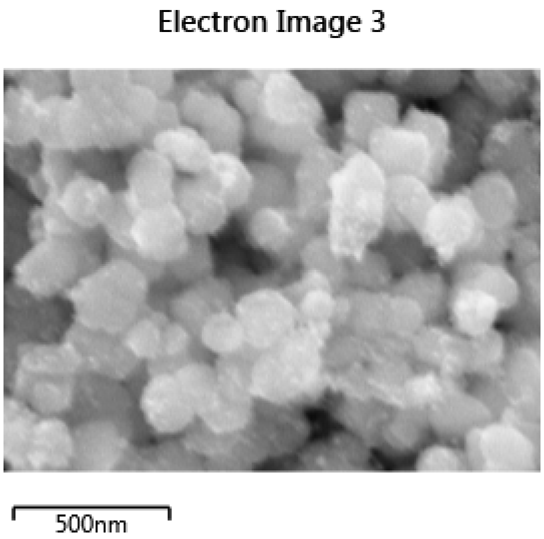
Electron Image 3
500nm

(f)

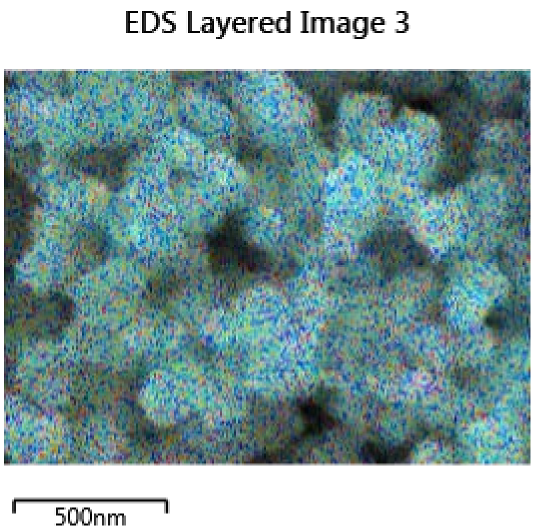
EDS Layered Image 3
500nm

7

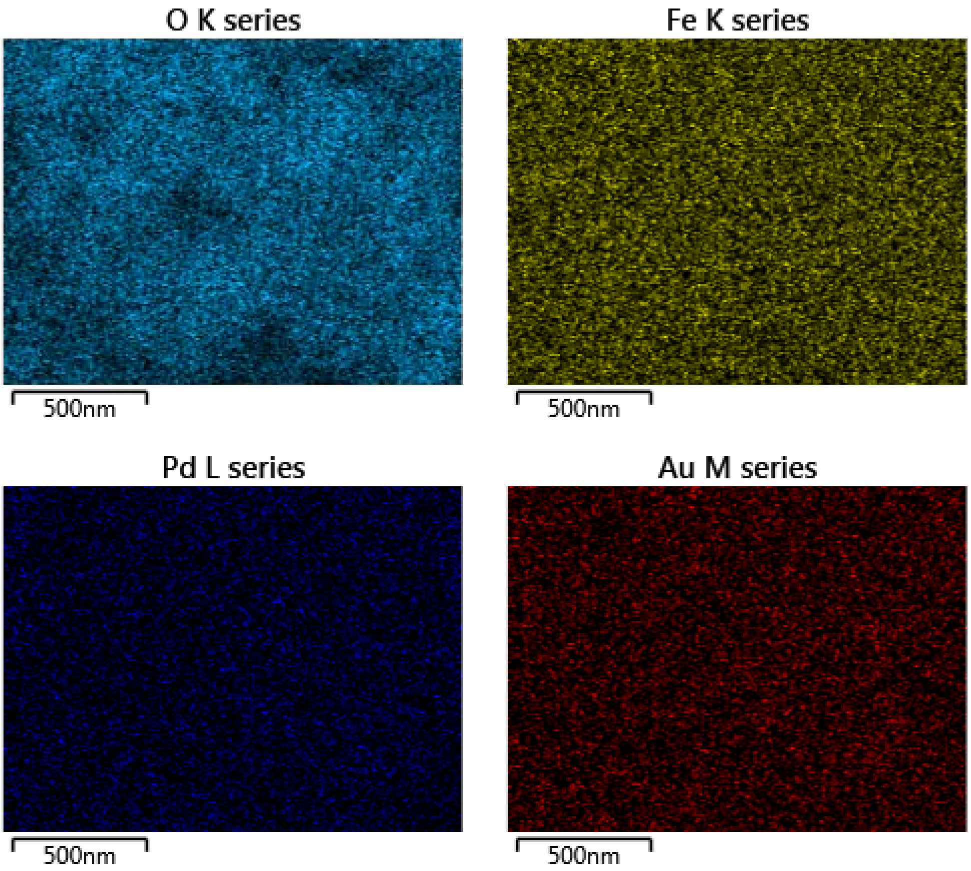
O K series
500nm
Fe K series
500nm
Pd L series
500nm
Au M series
500nm

(g) (h)

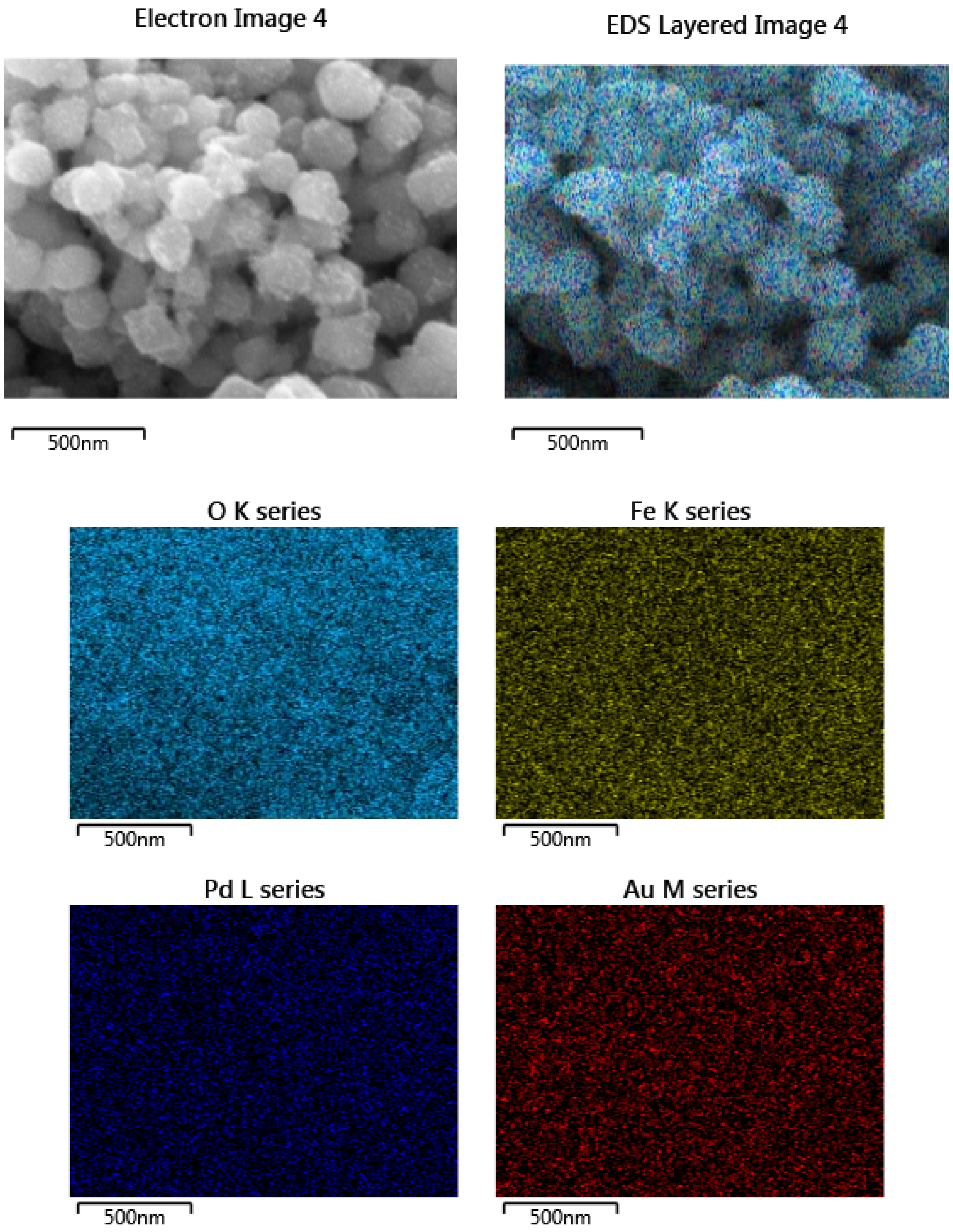

Electron Image 4
EDS Layered Image 4
500nm
500nm
O K series
Fe K series
500nm
500nm
Pd L series
Au M series
500nm
500nm

(i)

	Au (wt%)	Pd (wt%)	Molar ratio
$Au_1Pd_{0.23}–Fe_3O_4$	15.1	1.89	1:0.23
$Au_1Pd_{0.39}–Fe_3O_4$	12.4	2.62	1:0.39
$Au_1Pd_{1.08}–Fe_3O_4$	8.91	5.19	1:1.08
$Au_{0.65}Pd_1–Fe_3O_4$	6.54	5.46	0.65:1
$Au_{0.39}Pd_1–Fe_3O_4$	4.39	6.14	0.39:1

Figure S10. SEM and EDS images of (a) and (b) $Au_1Pd_{0.23}–Fe_3O_4$ NPs; (c) and (d) $Au_1Pd_{0.39}–Fe_3O_4$ NPs; (e) and (f) $Au_{0.65}Pd_1–Fe_3O_4$ NPs; (g) and (h) $Au_{0.39}Pd_1–Fe_3O_4$ NPs; (i) ICP-AES data of $Au_xPd_y–Fe_3O_4$ NPs

(a) (b)

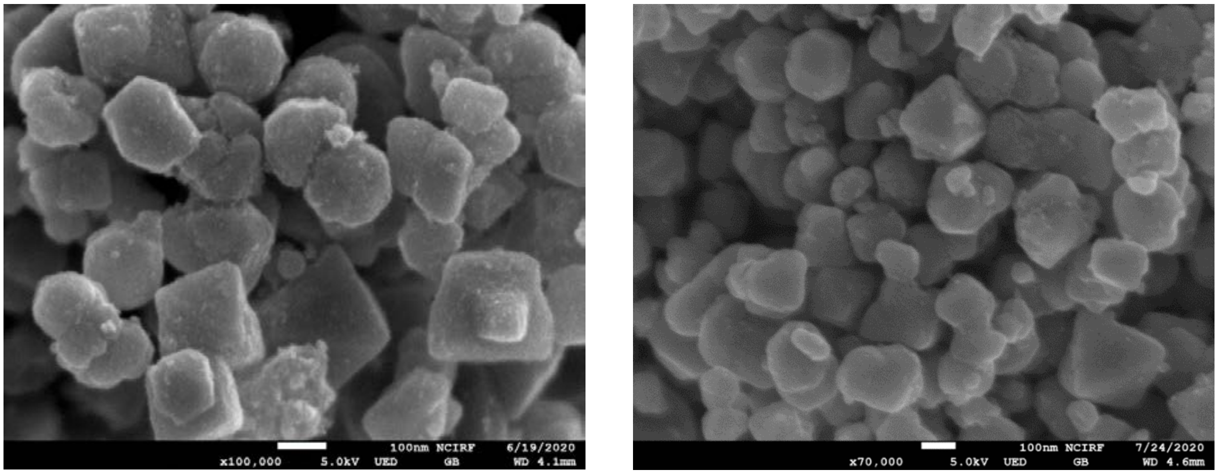

Figure S11. SEM images of AuPd–Fe_3O_4 NPs after 18 h under 0.50 mmol *N*-methyl-1-phenylmethanamine, 2.4 mol% AuPd–Fe_3O_4, 3.0 equiv $CsOH·H_2O$ after (a) 1st reaction, (b) 10th reaction

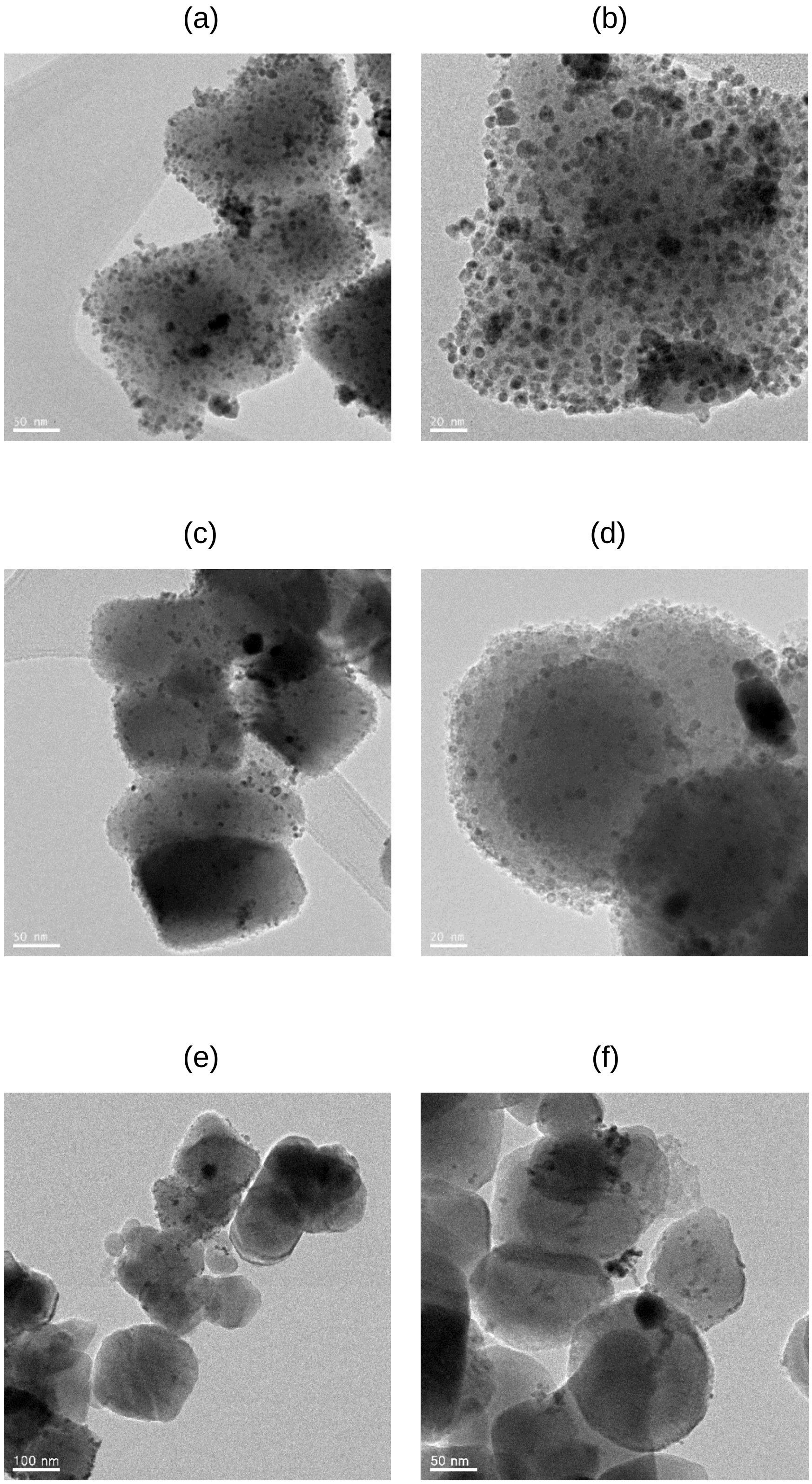

Figure S12. HR-TEM images of (a) and (b) fresh AuPd–Fe_3O_4 NPs; (c) and (d) AuPd–Fe_3O_4 NPs after 1 catalytic cycle; (e) and (f) AuPd–Fe_3O_4 NPs after 10 catalytic cycle

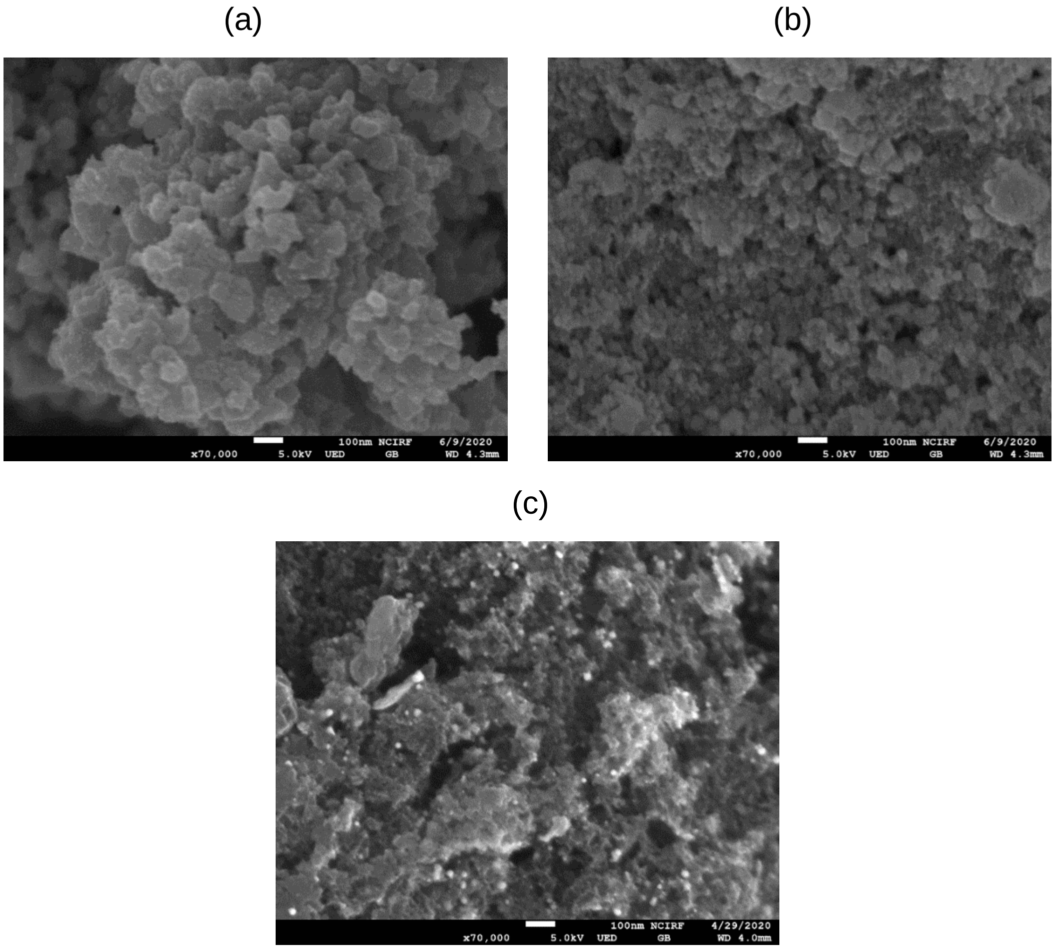

Figure S13. SEM images of (a) AuPd–TiO_2 NPs, (b) AuPd–CeO_2 NPs, and (c) AuPd–C NPs

	**Au (wt%)**	**Pd (wt%)**	**Au:Pd**
AuPd–TiO_2	10.3	4.51	1:0.81
AuPd–CeO_2	10.8	5.46	1:0.93
AuPd–C	2.07	1.21	1:1.08

Table S4. ICP-AES data of AuPd NPs with different supports

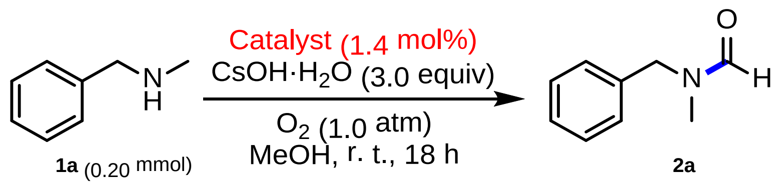

Entry	Catalyst	Au:Pd	Yield (%)
1	AuPd–TiO_2	1:0.81	55
2	AuPd–CeO_2	1:0.93	80
3	AuPd–C	1:1.08	48

Table S5. Formylation data of AuPd NPs with different supports

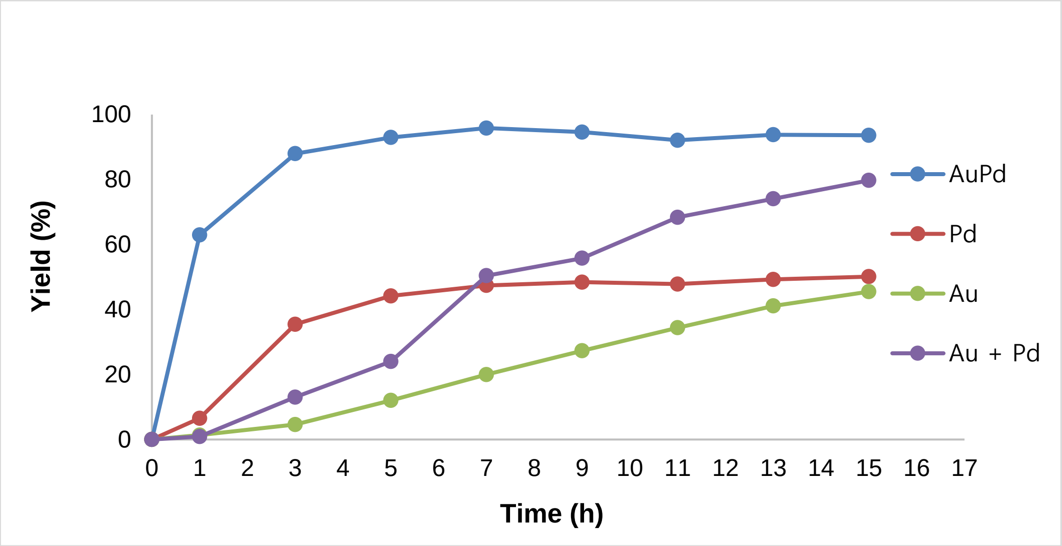

Figure S14. Kinetic data of *N*-formylation of *N*-methyl-1-phenylmethanamine (1.0 mmol scale) with AuPd–Fe_3O_4 NPs (1.4 mol% each of Au and Pd), Au–Fe_3O_4 NPs (2.8 mol%), Pd–Fe_3O_4 NPs (2.8 mol%), and a mixture of Au–Fe_3O_4 NPs (1.4 mol%) with Pd–Fe_3O_4 NPs (1.4 mol%)

Table S6. Substrate scope under high substrate concentration

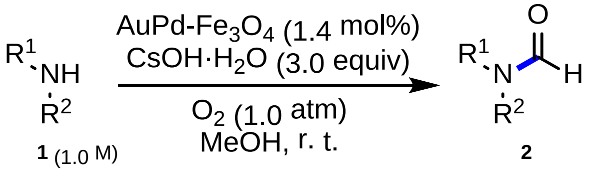

Entry	Substrate	Yield (%)[b]	Entry	Substrate	Yield (%)[b]
1[c]	**1a**	89[d] (79)	12	**1l**	78
2	**1b**	69	13	**1m**	79
3	**1c**	83	14	**1n**	59
4	**1d**	77	15	**1o**	22
5	**1e**	81	16	**1p**	67
6	**1f**	73	17	**1q**	81
7[e]	**1g**	87	18	**1r**	81
8	**1h**	78	19	**1s**	9
9	**1i**	82	20	**1t**	46
10	**1j**	76	21	**1u**	37
11	**1k**	71			

[a] Reaction conditions: Amine (1.0 mmol), AuPd–Fe_3O_4 (1.4 mol%), O_2 (1.0 atm), CsOH·H_2O (3.0 equiv), methanol (1.0 mL), r. t., 8 h.
[b] Yield of Isolated products.
[c] 4 h
[d] Determined from ^{1}H NMR spectral analysis through the use of mesitylene as an internal standard.
[e] 6 h

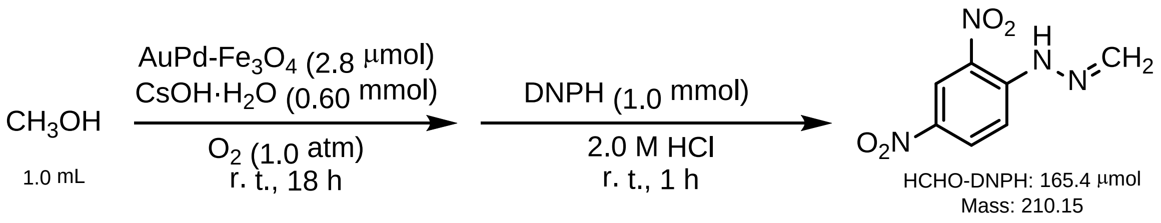

CH3OH
1.0 mL
AuPd-Fe3O4 (2.8 μmol)
CsOH·H2O (0.60 mmol)
O2 (1.0 atm)
r. t., 18 h
DNPH (1.0 mmol)
2.0 M HCl
r. t., 1 h
NO2
H
N
N
CH2
O2N
HCHO-DNPH: 165.4 μmol
Mass: 210.15

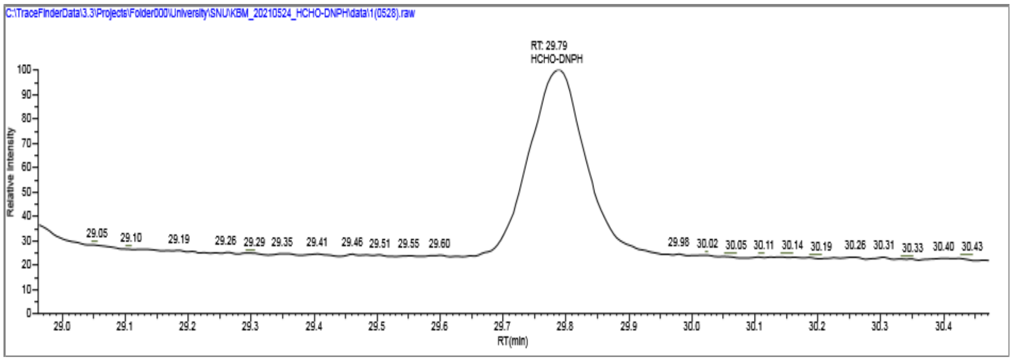

RT: 29.79
HCHO-DNPH
Relative Intensity
RT(min)

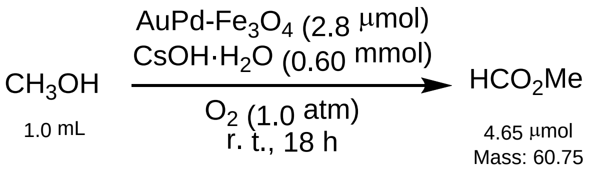

CH3OH
1.0 mL
AuPd-Fe3O4 (2.8 μmol)
CsOH·H2O (0.60 mmol)
O2 (1.0 atm)
r. t., 18 h
HCO2Me
4.65 μmol
Mass: 60.75

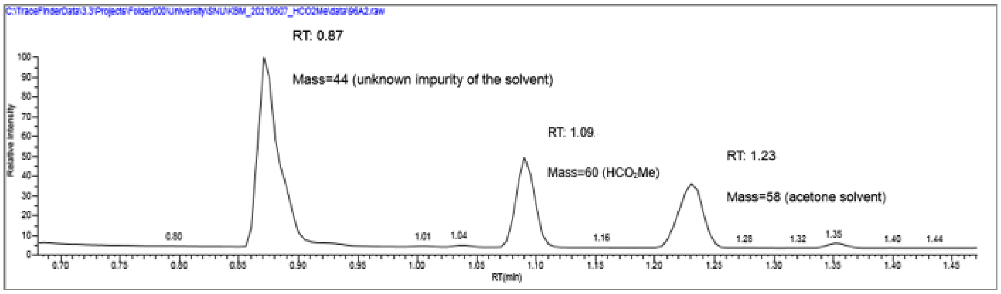

RT: 0.87
Mass=44 (unknown impurity of the solvent)
RT: 1.09
Mass=60 (HCO2Me)
RT: 1.23
Mass=58 (acetone solvent)
Relative Intensity
RT(min)

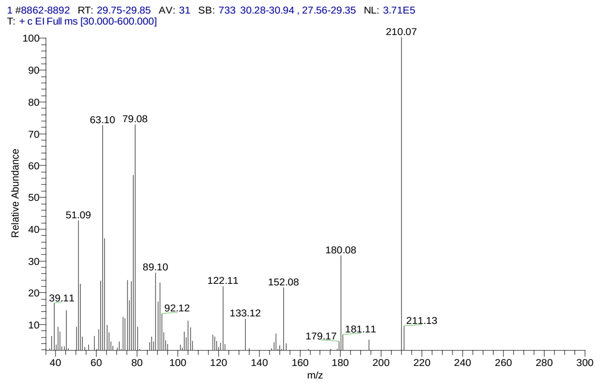

1 #8862-8892 RT: 29.75-29.85 AV: 31 SB: 733 30.28-30.94 , 27.56-29.35 NL: 3.71E5
T: + c EI Full ms [30.000-600.000]
210.07
63.10
79.08
51.09
89.10
122.11
152.08
180.08
39.11
92.12
133.12
179.17
181.11
211.13
Relative Abundance
m/z

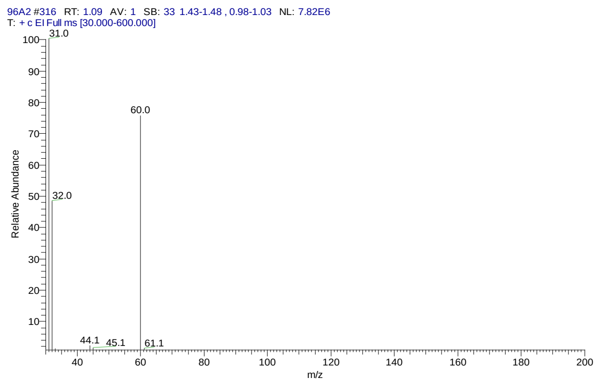

Figure S15. Determination of the methanol oxidation products

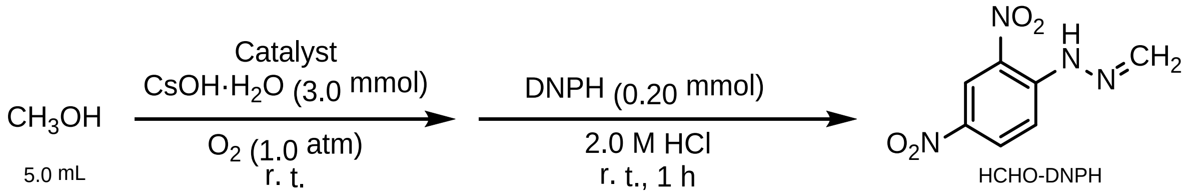

(a) Au-Fe_3O_4 (28 μmol)
(b) Pd-Fe_3O_4 (28 μmol)
(c) Au-Fe_3O_4 (14 μmol) + Pd-Fe_3O_4 (14 μmol)
(d) AuPd-Fe_3O_4 (14 μmol)

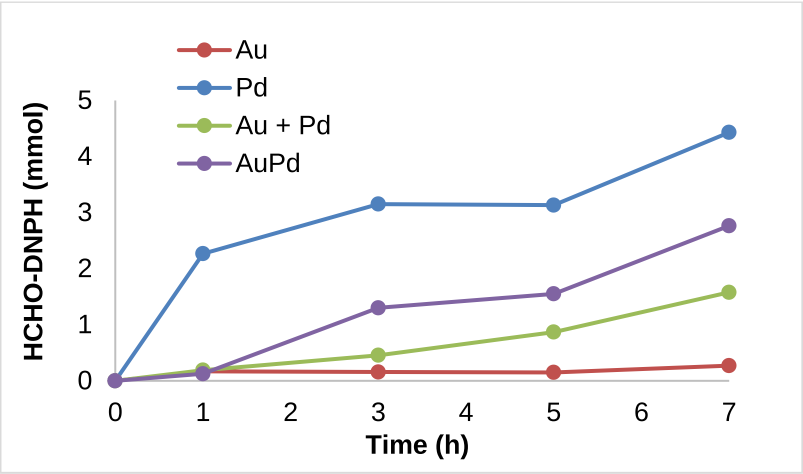

Figure S16. Initial kinetics of methanol oxidation to HCHO as measured from HCHO-DNPH generation

Table S7. Control experiments[a]

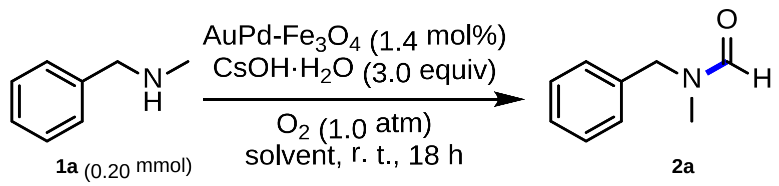

Entry	Condition	Product yield with the following as a solvent[b]		
		HCHO (37 wt% in H_2O)	HCO_2H	HCO_2Me
1	AuPd	52	< 0.5	71
2	AuPd, CsOH	78	< 0.5	75
3	CsOH	N. D.	N. D.	67

[a] *N*-Methyl-1-phenylmethanamine (0.20 mmol), AuPd–Fe_3O_4 (1.4 mol%) $CsOH{\cdot}H_2O$ (3.0 equiv), solvent (1.0 mL), O_2 (1.0 atm), r. t., 18 h.
[b] Determined from ^1H NMR spectral analysis through the use of mesitylene as an internal standard.

6. Characterization of Products

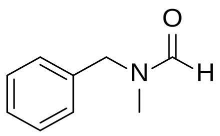

N-benzyl-N-methylformamide (2a)

Yellow oil. The compound was identified by spectral comparison with literature data.[4]

^{1}H NMR (500 MHz, $CDCl_3$) δ 8.29 (s, 0.520H, major rotamer), 8.17 (s, 0.380H, minor rotamer), 7.40 – 7.19 (m, 5.00H), 4.53 (s, 0.870H, minor rotamer), 4.40 (s, 1.15H, major rotamer), 2.85 (s, 1.30H, minor rotamer), 2.79 (s, 1.59H, major rotamer). ^{13}C NMR (101 MHz, $CDCl_3$) δ 162.83, 162.67, 136.08, 135.83, 128.97, 128.76, 128.24, 127.71, 127.47, 53.56, 47.84, 34.13, 29.53.

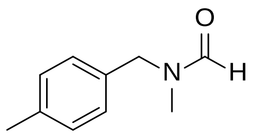

N-methyl-N-(4-methylbenzyl)formamide (2b)

Yellow oil. The compound was identified by spectral comparison with literature data.[5]

^{1}H NMR (400 MHz, $CDCl_3$) δ 8.27 (s, 0.620H, major rotamer), 8.14 (s, 0.410H, minor rotamer), 7.21 – 7.11 (m, 3.00H), 7.09 (d, *J* = 7.9 Hz, 1.00H), 4.47 (s, 0.910H, minor rotamer), 4.34 (s, 1.32H, major rotamer), 2.82 (s, 1.40H, minor rotamer), 2.76 (s, 1.83H, major rotamer), 2.34 (d, *J* = 6.5 Hz, 3.00H, mixture of rotamers). ^{13}C NMR (101 MHz, $CDCl_3$) δ 162.75, 162.58, 137.93, 137.39, 133.08, 132.76, 129.62, 129.41, 128.34, 127.46, 53.31, 47.53, 34.03, 29.42, 21.16.

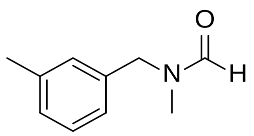

N-methyl-N-(3-methylbenzyl)formamide (2c)

Yellow oil.

^{1}H NMR (400 MHz, $CDCl_3$) δ 8.28 (s, 0.590H, major rotamer), 8.16 (s, 0.410H, minor rotamer), 7.27 – 7.20 (m, 1.00H), 7.14 – 6.99 (m, 3.00H), 4.48 (s, 0.910H, minor rotamer),

4.35 (s, 1.28H, major rotamer), 2.84 (s, 1.37H, minor rotamer), 2.78 (s, 1.79H, major rotamer), 2.34 (d, J = 6.5 Hz, 3.00H, mixture of rotamers). ^{13}C NMR (101 MHz, $CDCl_3$) δ 162.84, 162.64, 138.78, 138.52, 136.05, 135.81, 129.02, 128.92, 128.86, 128.63, 128.48, 128.13, 125.41, 124.58, 53.54, 47.80, 34.16, 29.56, 21.46. HRMS (ESI) calculated for $C_{10}H_{13}NO$ $[M]^+$ 163.0997, found 163.0997.

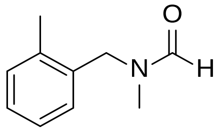

***N*-methyl-*N*-(2-methylbenzyl)formamide (2d)**

Yellow oil.

^{1}H NMR (400 MHz, $CDCl_3$) δ 8.24 (s, 0.570H, major rotamer), 8.16 (s, 0.420H, minor rotamer), 7.26 – 7.08 (m, 4.00H), 4.54 (s, 0.950H, minor rotamer), 4.40 (s, 1.20H, major rotamer), 2.79 (d, J = 5.3 Hz, 3.00H, mixture of rotamers), 2.30 (d, J = 2.5 Hz, 3.00H, mixture of rotamers). ^{13}C NMR (101 MHz, $CDCl_3$) δ 163.06, 162.42, 136.72, 136.19, 133.54, 133.46, 130.84, 130.59, 128.71, 128.07, 127.86, 127.76, 126.37, 126.10, 51.27, 45.62, 34.02, 29.56, 19.18, 19.12. HRMS (ESI) calculated for $C_{10}H_{13}NO$ $[M]^+$ 163.0997, found 163.1000.

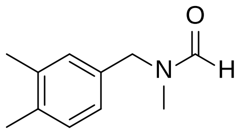

***N*-(3,4-dimethylbenzyl)-*N*-methylformamide (2e)**

Pale yellow oil.

^{1}H NMR (400 MHz, $CDCl_3$) δ 8.26 (s, 0.570H, major rotamer), 8.13 (s, 0.370H, minor rotamer), 7.12 – 7.07 (m, 1.00H), 7.03 – 6.90 (m, 2.00H), 4.45 (s, 0.760H, minor rotamer), 4.31 (s, 1.16H, major rotamer), 2.82 (s, 1.22H, minor rotamer), 2.76 (s, 1.68H, major rotamer), 2.24 (d, J = 6.0 Hz, 6.00H, mixture of rotamers). ^{13}C NMR (101 MHz, $CDCl_3$) δ 162.72, 162.53, 137.22, 136.96, 136.50, 135.98, 133.46, 133.15, 130.07, 129.86, 129.60, 128.69, 125.81, 124.93, 53.26, 47.47, 34.02, 29.40, 19.77, 19.73, 19.45. HRMS (ESI) calculated for $C_{11}H_{15}NO$ $[M]^+$ 177.1154, found 177.1154.

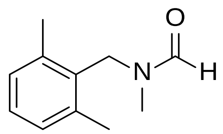

***N*-(2,6-dimethylbenzyl)-*N*-methylformamide (2f)**

Yellow oil.

^{1}H NMR (400 MHz, $CDCl_3$) δ 8.15 (s, 0.460H, major rotamer), 8.11 (s, 0.430H, minor rotamer), 7.13 (dd, *J* = 15.7, 8.8 Hz, 1.00H), 7.05 (t, *J* = 7.1 Hz, 2.00H), 4.64 (s, 0.980H, minor rotamer), 4.45 (s, 1.07H, major rotamer), 2.72 (s, 1.53H, major rotamer), 2.64 (s, 1.49H, minor rotamer), 2.34 (s, 3.06H, major rotamer), 2.31 (s, 2.88H, minor rotamer). ^{13}C NMR (101 MHz, $CDCl_3$) δ 162.79, 162.33, 138.15, 137.84, 131.46, 130.82, 128.95, 128.59, 128.41, 128.12, 127.95, 126.78. 53.64, 47.52, 41.25, 40.35, 32.78, 28.84, 20.27, 20.17, 20.10. HRMS (ESI) calculated for $C_{11}H_{15}NO$ $[M]^+$ 177.1154, found 177.1155.

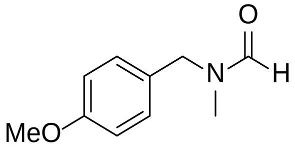

***N*-(4-methoxybenzyl)-*N*-methylformamide (2g)**

Yellow oil. The compound was identified by spectral comparison with literature data.[4]

^{1}H NMR (400 MHz, $CDCl_3$) δ 8.27 (s, 0.590H, major rotamer), 8.12 (s, 0.410H, minor rotamer), 7.18 (d, *J* = 8.3 Hz, 1.00H), 7.13 (d, *J* = 8.4 Hz, 1.00H), 6.88 (dd, *J* = 12.5, 8.4 Hz, 2.00H), 4.45 (s, 0.870H, minor rotamer), 4.32 (s, 1.18H, major rotamer), 3.80 (d, *J* = Hz, 3.00H, mixture of rotamers), 2.82 (s, 1.34H, minor rotamer), 2.75 (s, 1.69H, major rotamer). ^{13}C NMR (101 MHz, $CDCl_3$) δ 162.64, 162.54, 159.50, 159.17, 129.69, 128.82, 128.19, 127.69, 114.31, 114.09, 55.35, 55.33, 53.01, 47.19, 33.96, 29.29.

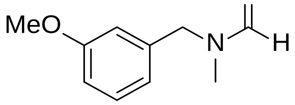

***N*-(3-methoxybenzyl)-*N*-methylformamide (2h)**

Yellow oil.

^{1}H NMR (400 MHz, $CDCl_3$) δ 8.20 (s, 0.540H, major rotamer), 8.09 (s, 0.400H, minor rotamer), 7.26 – 7.15 (m, 1.00H), 6.83 – 6.65 (m, 3.00H), 4.43 (s, 0.880H, minor rotamer),

4.30 (s, 1.12H, major rotamer), 3.74 (d, 3.00H, mixture of rotamers), 2.79 (s, 1.43H, minor rotamer), 2.73 (s, 1.60H, major rotamer). ^{13}C NMR (101 MHz, $CDCl_3$) δ 162.74, 162.56, 160.04, 159.90, 137.59, 137.43, 129.94, 129.66, 120.46, 119.58, 113.67, 113.30, 113.08, 113.03, 55.23, 55.19, 53.37, 47.66, 34.05, 29.45. HRMS (ESI) calculated for $C_{10}H_{13}NO_2$ $[M]^+$ 179.0946, found 179.0947.

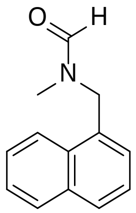

***N*-methyl-*N*-(naphthalen-1-ylmethyl)formamide (2i)**

Yellow oil. The compound was identified by spectral comparison with literature data.[4]

^{1}H NMR (400 MHz, $CDCl_3$) δ 8.37 (s, 0.420H, minor rotamer), 8.15 (s, 0.570H, major rotamer), 8.10 – 7.79 (m, 3.00H), 7.58 – 7.27 (m, 4.00H), 4.97 (s, 1.24H, major rotamer), 4.85 (s, 0.830H, minor rotamer), 2.84 (s, 1.20H, major rotamer), 2.72 (s, 1.81H, minor rotamer). ^{13}C NMR (101 MHz, $CDCl_3$) δ 163.24, 162.35, 133.91, 131.61, 131.38, 131.15, 131.09, 129.11, 128.89, 128.85, 128.71, 127.70, 126.76, 126.72, 126.15, 125.60, 125.38, 125.14, 123.87, 122.34, 51.01, 45.87, 34.01, 30.03.

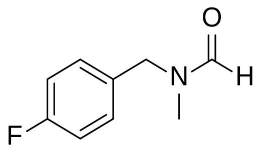

***N*-(4-fluorobenzyl)-*N*-methylformamide (2j)**

Yellow oil. The compound was identified by spectral comparison with literature data.[4]

^{1}H NMR (400 MHz, $CDCl_3$) δ 8.28 (s, 0.500H, major rotamer), 8.15 (s, 0.450H, minor rotamer), 7.21 (m, 2.00H), 7.04 (m, 2.00H), 4.49 (s, 0.990H, minor rotamer), 4.37 (s, 1.06H, major rotamer), 2.85 (s, 1.45H, minor rotamer), 2.77 (s, 1.49H, major rotamer). ^{13}C NMR (101 MHz, $CDCl_3$) δ 163.80, 163.56, 162.64 (d, J = 5.6 Hz), 161.34, 161.11, 131.95 (d, J = 3.2 Hz), 131.57 (d, J = 3.2 Hz), 130.02 (d, J = 8.2 Hz), 129.19 (d, J = 8.3 Hz), 116.02, 115.76 (d, J = 7.7 Hz), 115.51, 52.83, 47.12, 34.03, 29.39. ^{19}F NMR (376 MHz, $CDCl_3$) δ -114.01 – -114.14 (m), -114.72 – -114.87 (m).

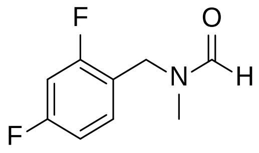

N-(2,4-difluorobenzyl)-N-methylformamide (2k)

Yellow oil.

^{1}H NMR (400 MHz, $CDCl_3$) δ 8.28 (s, 0.510H, major rotamer), 8.12 (s, 0.460H, minor rotamer), 7.34 – 7.17(m, 1.00H), 6.85 (tdd, *J* = 18.5, 13.1, 5.2 Hz, 2.00H), 4.55 (s, 0.940H, minor rotamer), 4.42 (s, 1.04H, major rotamer), 2.91 (s, 1.46H, minor rotamer), 2.79 (s, 1.54H, major rotamer). ^{13}C NMR (101 MHz, $CDCl_3$) δ 164.19, 164.07, 163.77, 163.65, 162.76, 162.71, 162.70, 162.58, 162.45, 162.33, 161.70, 161.59, 161.30, 161.18, 160.09, 159.97, 159.85, 131.74 (dd, *J* = 9.7, 5.7 Hz), 130.76 (dd, *J* = 9.8, 5.5 Hz), 111.81 (t, *J* = 3.7 Hz), 111.60 (t, *J* = 3.8 Hz), 104.67, 104.41, 104.16, 104.04, 103.78, 103.52, 46.86 (d, *J* = 3.3 Hz), 40.64 (d, *J* = 3.3 Hz), 34.23, 29.25 (d, *J* = 2.0 Hz). ^{19}F NMR (376 MHz, $CDCl_3$) δ -109.34 – -109.84 (m), -110.35 – -110.48 (m), -114.13 (dd, J = 17.7, 8.7 Hz), -114.69 (dd, J = 17.2, 8.5 Hz). HRMS (ESI) calculated for $C_9H_9N_2O$ $[M]^+$ 185.0652, found 185.0649.

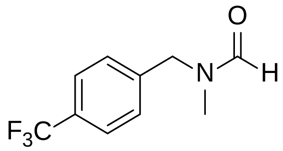

N-methyl-N-(4-(trifluoromethyl)benzyl)formamide (2l)

Colorless oil. The compound was identified by spectral comparison with literature data.[4]

^{1}H NMR (400 MHz, $CDCl_3$) δ 8.31 (s, 0.470H, minor rotamer), 8.19 (s, 0.500H, major rotamer), 7.62 (dd, *J* = 18.5, 8.1 Hz, 2.0H), 7.37 (dd, *J* = 11.4, 8.3 Hz, 2.0H), 4.58 (s, 1.13H, major rotamer), 4.48 (s, 0.980H, minor rotamer), 2.89 (s, 1.67H, major rotamer), 2.80 (s, 1.37H, minor rotamer). ^{13}C NMR (101 MHz, $CDCl_3$) δ 162.78, 162.70, 140.27 (d, *J* = 1.2 Hz), 140.09 (d, *J* = 1.2 Hz), 130.56, 130.24, 130.06, 129.73, 128.39, 127.68, 125.89 (q, *J* = 3.8 Hz), 125.65 (q, *J* = 3.8 Hz), 125.43, 125.31, 122.72, 122.60, 52.93, 47.40, 34.13, 29.54. ^{19}F NMR (376 MHz, $CDCl_3$) δ -62.62, -62.68.

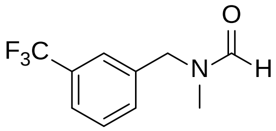

N-methyl-N-(3-(trifluoromethyl)benzyl)formamide (2m)

Colorless oil.

^{1}H NMR (400 MHz, $CDCl_3$) δ 8.31 (s, 0.420H, minor rotamer), 8.19 (s, 0.450H, major rotamer), 7.61 – 7.41 (m, 4.00H), 4.58 (s, 1.02H, major rotamer), 4.47 (s, 0.880H, minor rotamer), 2.89 (s, 1.56H, major rotamer), 2.80 (s, 1.30H, minor rotamer). ^{13}C NMR (101 MHz, $CDCl_3$) δ 162.78 (d, *J* = 2.3 Hz), 137.27, 137.08, 131.60 (d, *J* = 1.2 Hz), 131.30 (d, *J* = 1.2 Hz), 130.99, 130.73 (d, *J* = 1.1 Hz), 129.61, 129.36, 125.39, 125.27, 125.11 (q, *J* = 3.8 Hz), 124.85 (q, *J* = 3.8 Hz), 124.63 (q, *J* = 3.8 Hz), 124.21 (q, *J* = 3.8 Hz), 122.68, 122.56, 53.07, 47.48, 34.19, 29.60. ^{19}F NMR (376 MHz, $CDCl_3$) δ -62.68, -62.76. HRMS (ESI) calculated for $C_{10}H_{10}F_3NO$ $[M]^+$ 217.0714, found 217.0716.

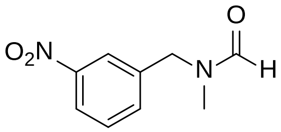

N-methyl-N-(3-nitrobenzyl)formamide (2n)

Yellow oil.

^{1}H NMR (400 MHz, $CDCl_3$) δ 8.34 (s, 0.390H, minor rotamer), 8.22 (s, 0.720H, major rotamer, 8.20 – 8.11 (m, 2.00H), 7.64 – 7.53 (m, 2.00H), 4.64 (s, 1.24H, major rotamer), 4.54 (s, 0.850H, minor rotamer), 2.94 (s, 1.80H, major rotamer), 2.83 (s, 1.17H, minor rotamer). ^{13}C NMR (101 MHz, $CDCl_3$) δ 162.85, 162.79, 148.70, 148.52, 138.41, 138.26, 134.29, 133.34, 130.18, 129.93, 123.31, 122.85, 122.84, 122.43, 52.79, 47.30, 34.38, 29.71. HRMS (ESI) calculated for $C_9H_{10}N_2O_3$ $[M]^+$ 194.0691, found 194.0690.

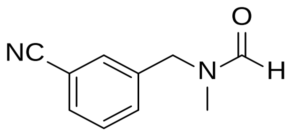

N-(3-cyanobenzyl)-N-methylformamide (2o)

Yellow oil. The compound was identified by spectral comparison with literature data.[6]

^{1}H NMR (400 MHz, $CDCl_3$) δ 8.30 (s, 0.390H, minor rotamer), 8.19 (s, 0.560H, major

rotamer), 7.67 – 7.44 (m, 4.00H), 4.56 (s, 1.23H, major rotamer), 4.45 (s, 0.890H, minor rotamer), 2.90 (s, 1.80H, major rotamer), 2.80 (s, 1.19H, minor rotamer). ^{13}C NMR (101 MHz, $CDCl_3$) δ 162.81, 162.74, 137.86, 137.68, 132.65, 131.98, 131.70, 131.57, 131.51, 130.97, 129.99, 129.74, 118.56, 118.32, 113.32, 112.98, 52.77, 47.31, 34.32, 29.70.

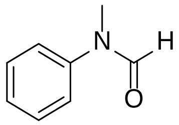

***N*-methyl-*N*-phenylformamide (2p)**

Brown oil. The compound was identified by spectral comparison with literature data.[7]

^{1}H NMR (400 MHz, $CDCl_3$) δ 8.48 (s, 1.00H), 7.42 (t, *J* = 7.8 Hz, 2.00H), 7.29 (d, *J* = 7.4 Hz, 1.00H), 7.18 (d, *J* = 7.7 Hz, 2.00H), 3.33 (s, 3.00H). ^{13}C NMR (126 MHz, $CDCl_3$) δ 162.33, 142.19, 129.63, 126.40, 122.36, 32.05.

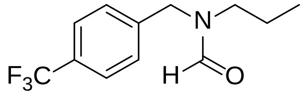

***N*-propyl-*N*-(4-(trifluoromethyl)benzyl)formamide (2q)**

Colorless oil.

^{1}H NMR (400 MHz, $CDCl_3$) δ 8.30 (s, 0.330H, minor rotamer), 8.23 (s, 0.620H, major rotamer), 7.64 – 7.58 (m, 2.00H), 7.39 – 7.34 (m, 2.00H), 4.59 (s, 1.43H, major rotamer), 4.47 (s, 0.670H, minor rotamer), 3.24 – 3.18 (m, 14.0H), 3.14 (t, *J* = 7.1 Hz, 26.0H), 1.61 – 1.48 (m, 2.00H), 0.90 – 0.86 (m, 3.00H). ^{13}C NMR (101 MHz, $CDCl_3$) δ 163.10, 163.00, 140.88 (d, *J* = 1.1 Hz), 140.62, 130.08, 129.76, 128.36, 127.74, 125.94 (q, *J* = 3.8 Hz), 125.69 (q, *J* = 3.8 Hz), 125.50, 122.79, 50.81, 48.87, 45.08, 43.85, 21.52, 20.25, 11.30, 10.94. ^{19}F NMR (376 MHz, $CDCl_3$) δ -62.60, -62.67. HRMS (ESI) calculated for $C_{12}H_{14}F_3NO$ $[M]^+$ 245.1027, found 245.1024.

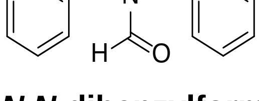

***N,N*-dibenzylformamide (2r)**

Colorless oil. The compound was identified by spectral comparison with literature data.[8]

^{1}H NMR (400 MHz, $CDCl_3$) δ 8.41 (s, 1H), 7.38 – 7.27 (m, 6.00H), 7.20 – 7.15 (m, 4.00H), 4.41 (s, 2.00H), 4.25 (s, 2.00H). ^{13}C NMR (101 MHz, $CDCl_3$) δ 162.85, 136.06, 135.69, 128.95, 128.73, 128.54, 128.17, 127.75, 127.69, 50.27, 44.68.

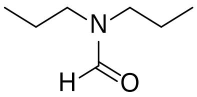

_N,N_-dipropylformamide (2s)

Yellow oil. The compound was identified by spectral comparison with literature data.[8]

^{1}H NMR (400 MHz, $CDCl_3$) δ 8.03 (s, 1.00H), 3.26 – 3.20 (m, 2.00H), 3.14 (t, *J* = 7.1 Hz, 2.00H), 1.57 – 1.51 (m, 4.00H), 0.88 (td, *J* = 7.4, 2.6 Hz, 6.00H). ^{13}C NMR (101 MHz, $CDCl_3$) δ 162.92, 49.27, 43.85, 21.93, 20.64, 11.42, 11.03.

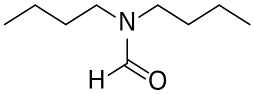

_N,N_-dibutylformamide (2t)

Yellow oil. The compound was identified by spectral comparison with literature data.[8]

^{1}H NMR (400 MHz, $CDCl_3$) δ 8.04 (s, 1.00H), 3.32 – 3.26 (m, 2.00H), 3.20 (t, *J* = 7.1 Hz, 2.00H), 1.57 – 1.47 (m, 4.00H), 1.37 – 1.28 (m, 4.00H), 0.98 – 0.91 (m, 6.00H). ^{13}C NMR (101 MHz, $CDCl_3$) δ 162.76, 47.25, 41.94, 30.83, 29.48, 20.25, 19.73, 13.89, 13.82.

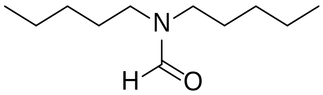

_N,N_-dipentylformamide (2u)

Yellow oil. The compound was identified by spectral comparison with literature data.[9]

^{1}H NMR (400 MHz, $CDCl_3$) δ 8.04 (s, 1.00H), 3.28 (m, 2.00H), 3.19 (t, *J* = 7.1 Hz, 2.00H), 1.56 – 1.52 (m, 4.00H), 1.37 – 1.23 (m, 8.00H), 0.93 – 0.88 (m, 6.00H). ^{13}C NMR (101 MHz, $CDCl_3$) δ 162.74, 47.49, 42.12, 29.15, 28.67, 28.39, 27.04, 22.47, 22.34, 14.05, 14.01.

II. References

[1] S. Mo, J. Xu, *ChemCatChem* **2014**, *6*, 1679.

[2] M. Takhi, K. Sreenivas, C. K. Reddy, M. Munikumar, K. Praveena, P. Sudheer, B. N.V.M. Rao, G. Ramakanth, J. Sivaranjani, S. Mulik, Y. R. Reddy, K. N. Rao, R. Pallavi, A. Lakshminarasimhan, S. K. Panigrahi, T. Antony, I. Abdullah, Y. K. Lee, M. Ramachandra, R. Yusof, N. A. Rahman, H. Subramanya, *Eur. J. Med. Chem.* **2014**, *84*, 382.

[3] K. Orito, M. Miyazawa, T. Nakamura, A. Horibata, H. Ushito, H. Nagasaki, M. Yuguchi, S. Yamashita, T. Yamazaki, M. Tokuda, *J. Org. Chem.* **2006**, *71*, 5951.

[4] S. Nakai, T. Yatabe, K. Suzuki, Y. Sasano, Y. Iwabuchi, J. Hasegawa, N. Mizuno, K. Yamaguchi, *Angew. Chem. Int. Ed.* **2019**, *58*, 16651.

[5] Z. Liu, Z. Yang, Z. Ke, X. Yu, H. Zhang, B. Yu, Y. Zhao, Z. Liu, *New J. Chem.,* **2018**, *42*, 13933.

[6] T. Yamashita, K. Shiomori, M. Yasuda, K. Shima, *Bull. Chem. Soc. Jpn*, **1991**, *64*, 366.

[7] T. B. Nguyen, J. Sorres, M. Q. Tran, L. Ermolenko, A. Al-Mourabit, *Org. Lett.* **2012**, *14*, 3202.

[8] Z. Ke, Y. Zhang, X. Cui, F. Shi, *Green Chem.,* **2016**, *18*, 808.

[9] J. Li, S. Cai, J. Chen, Y. Zhao, D. Z. Wang, *Synlett* **2014**, *25*, 1626.

III. NMR spectra

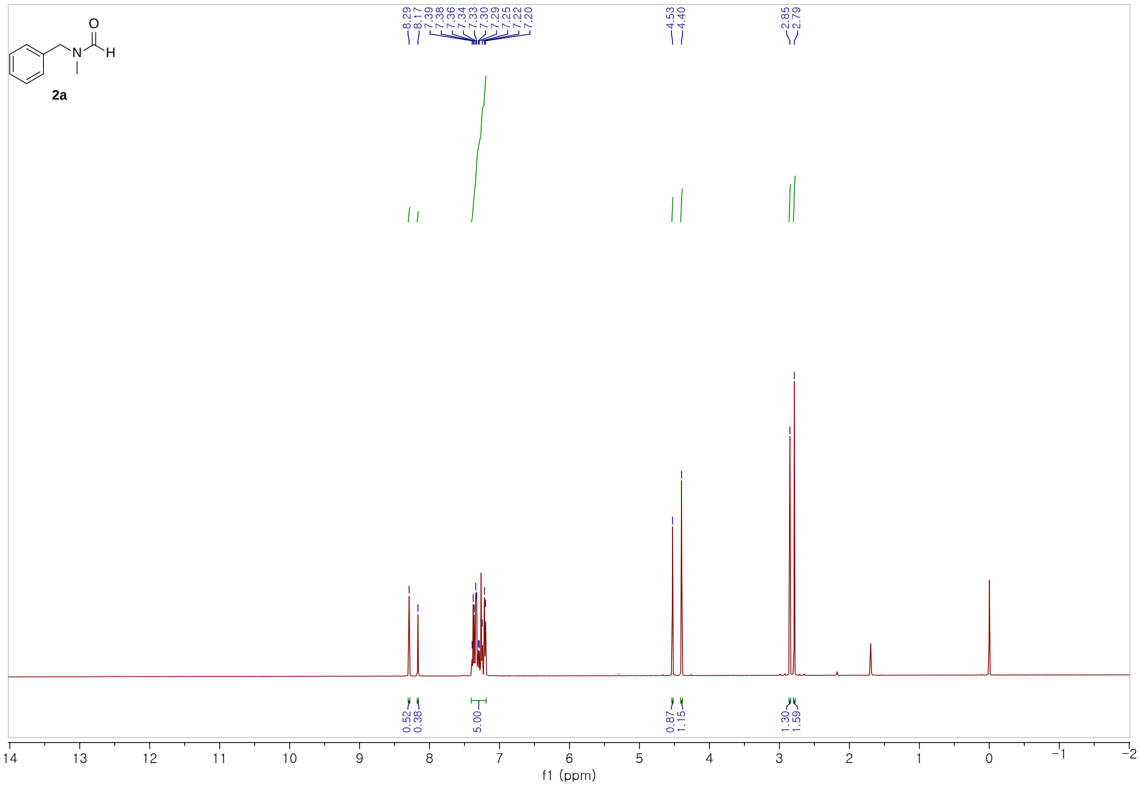

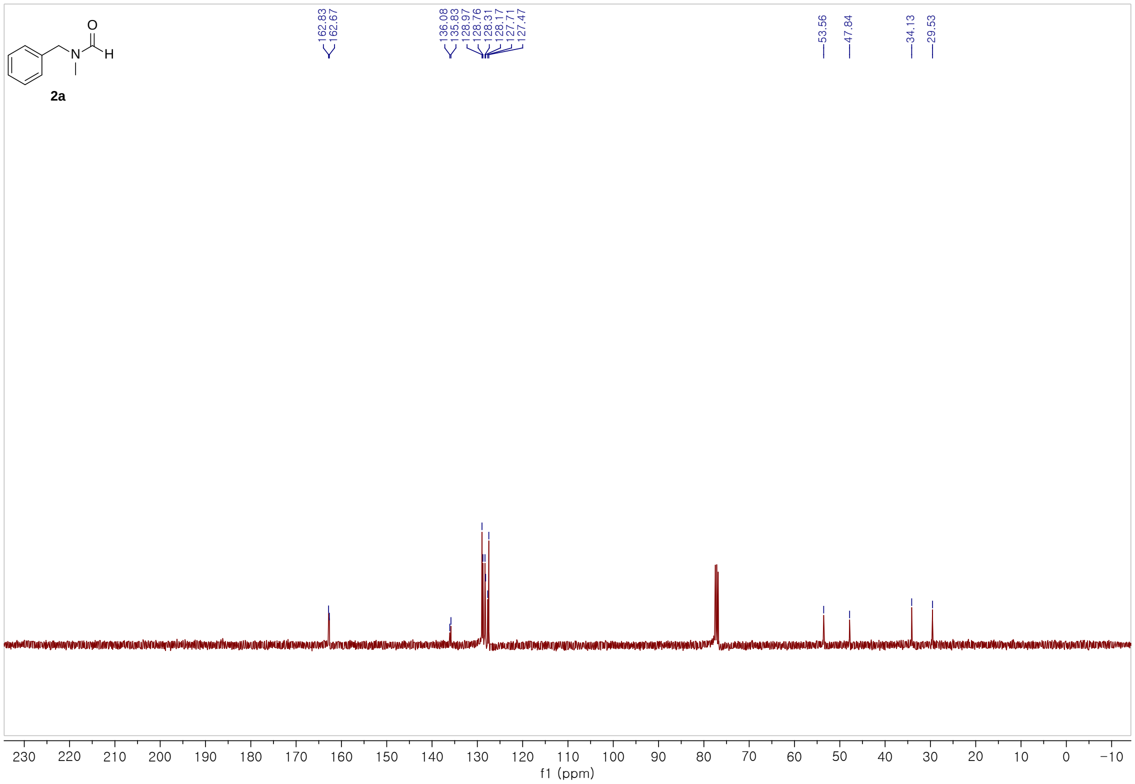

2a
162.83
162.67
136.08
135.83
128.97
128.76
128.31
128.17
127.71
127.47
53.56
47.84
34.13
29.53
f1 (ppm)

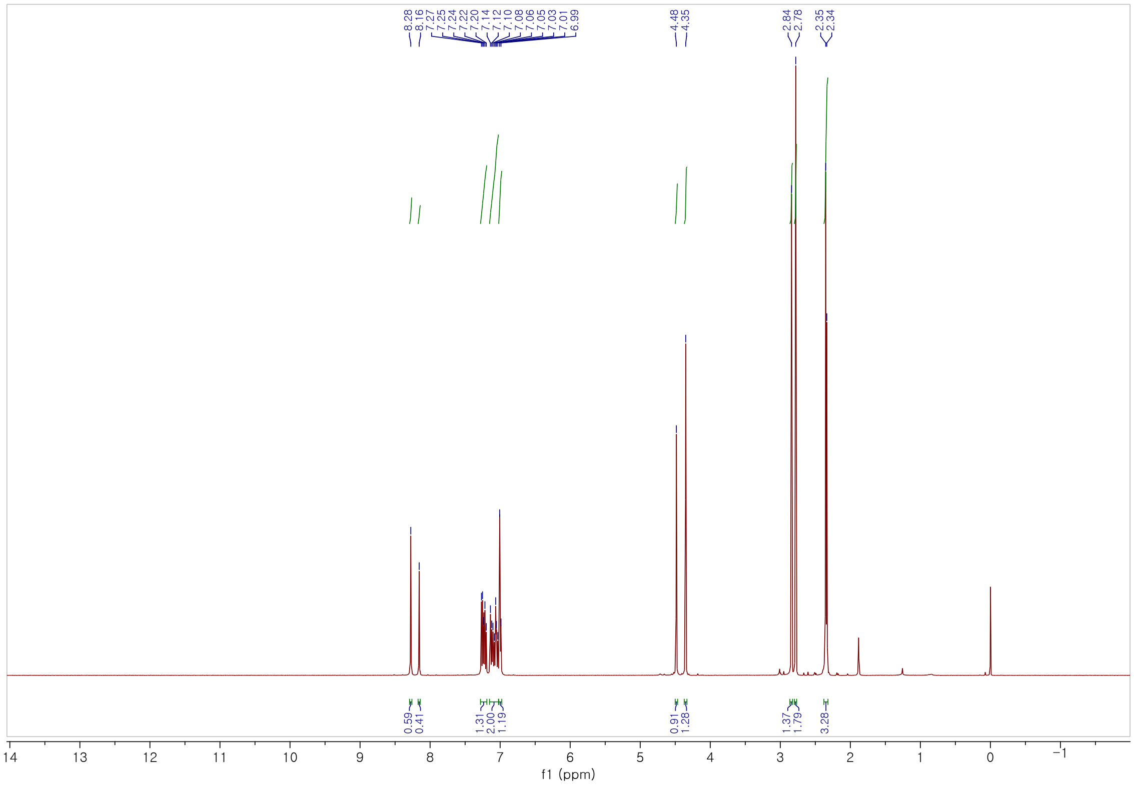

8.28
8.16
7.27
7.25
7.24
7.22
7.20
7.14
7.12
7.10
7.08
7.06
7.05
7.03
7.01
6.99
4.48
4.35
2.84
2.78
2.35
2.34
0.59
0.41
1.31
2.00
1.19
0.91
1.28
1.37
1.79
3.28
f1 (ppm)

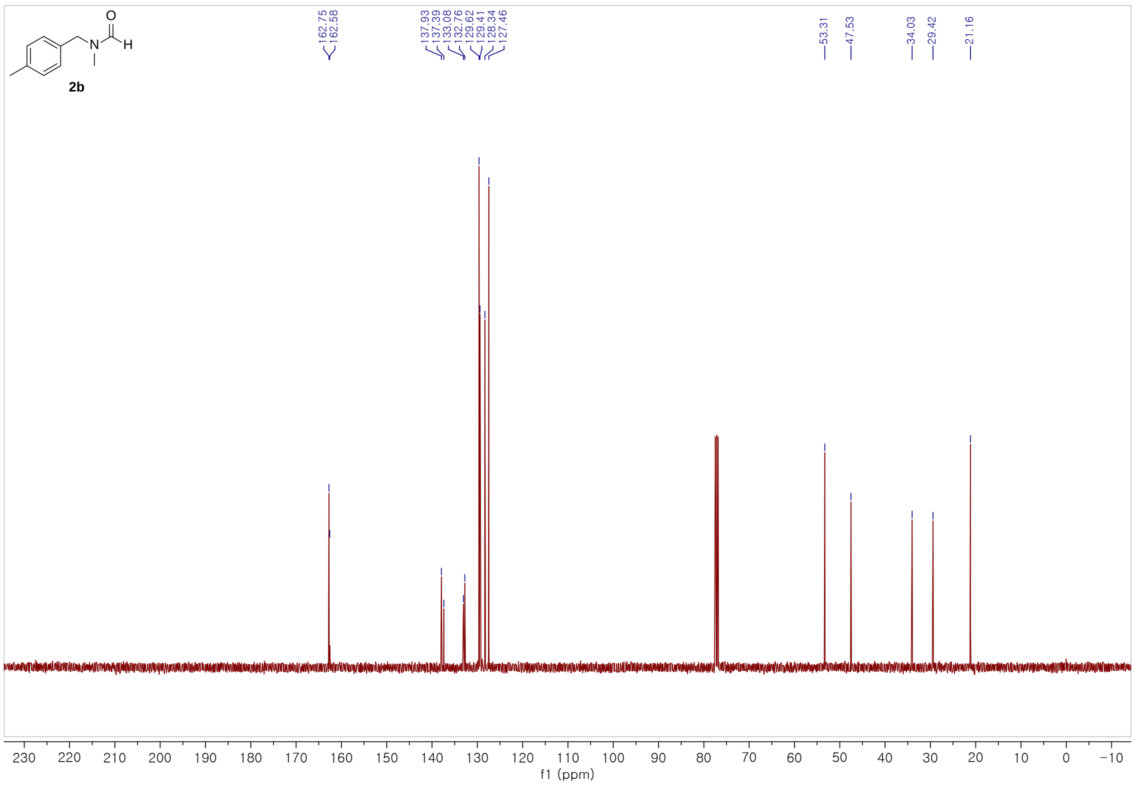
2b
162.75
162.58
137.93
137.39
133.08
132.76
129.62
129.41
128.34
127.46
53.31
47.53
34.03
29.42
21.16
f1 (ppm)

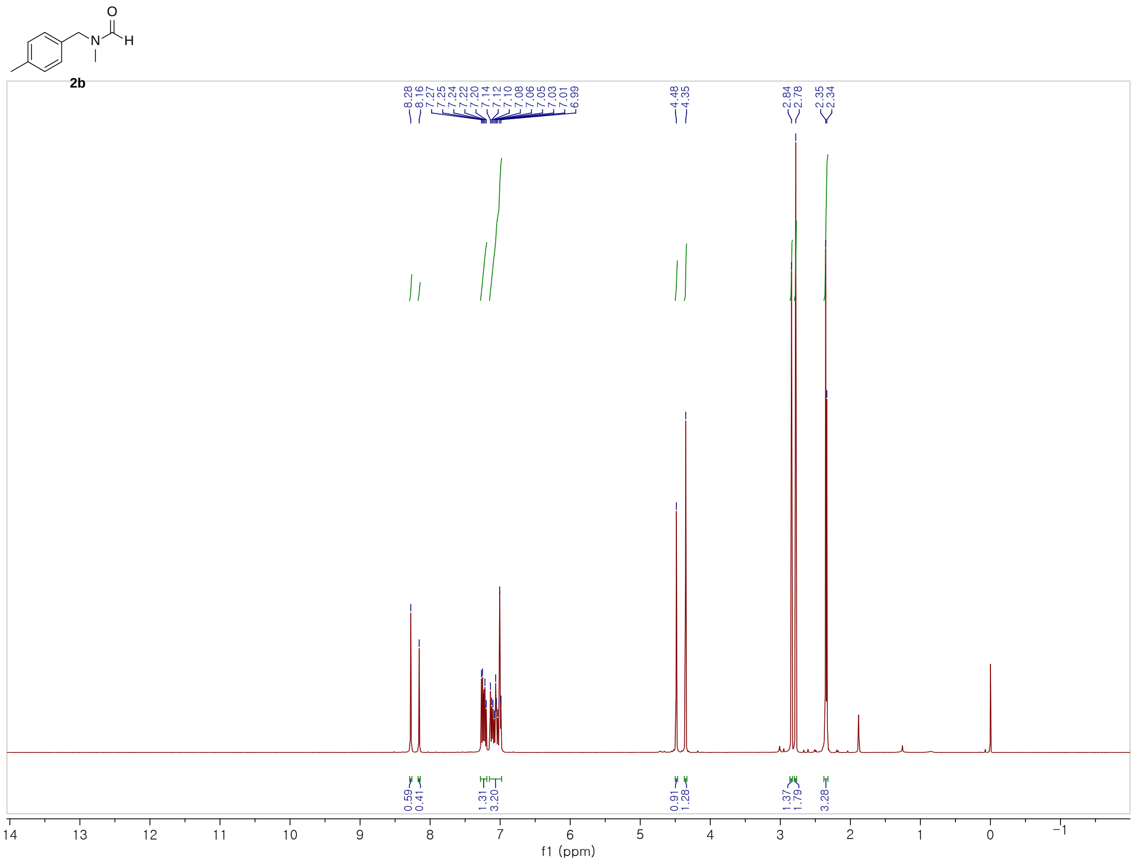
2b
8.28
8.16
7.27
7.25
7.24
7.22
7.20
7.14
7.12
7.10
7.08
7.06
7.05
7.03
7.01
6.99
4.48
4.35
2.84
2.78
2.35
2.34
0.59
0.41
1.31
3.20
0.91
1.28
1.37
1.79
3.28
f1 (ppm)

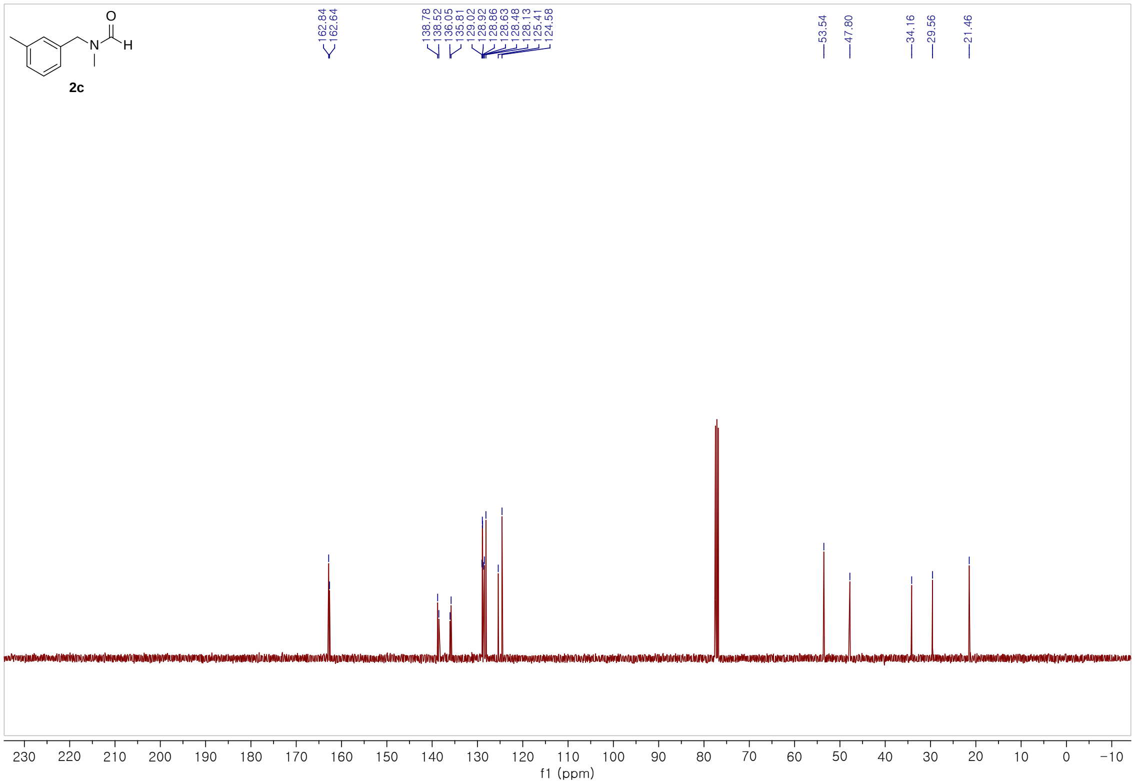
2c
162.84
162.64
138.78
138.52
136.05
135.81
129.02
128.92
128.86
128.63
128.48
128.13
125.41
124.58
53.54
47.80
34.16
29.56
21.46
230 220 210 200 190 180 170 160 150 140 130 120 110 100 90 80 70 60 50 40 30 20 10 0 −10
f1 (ppm)

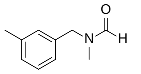

2c

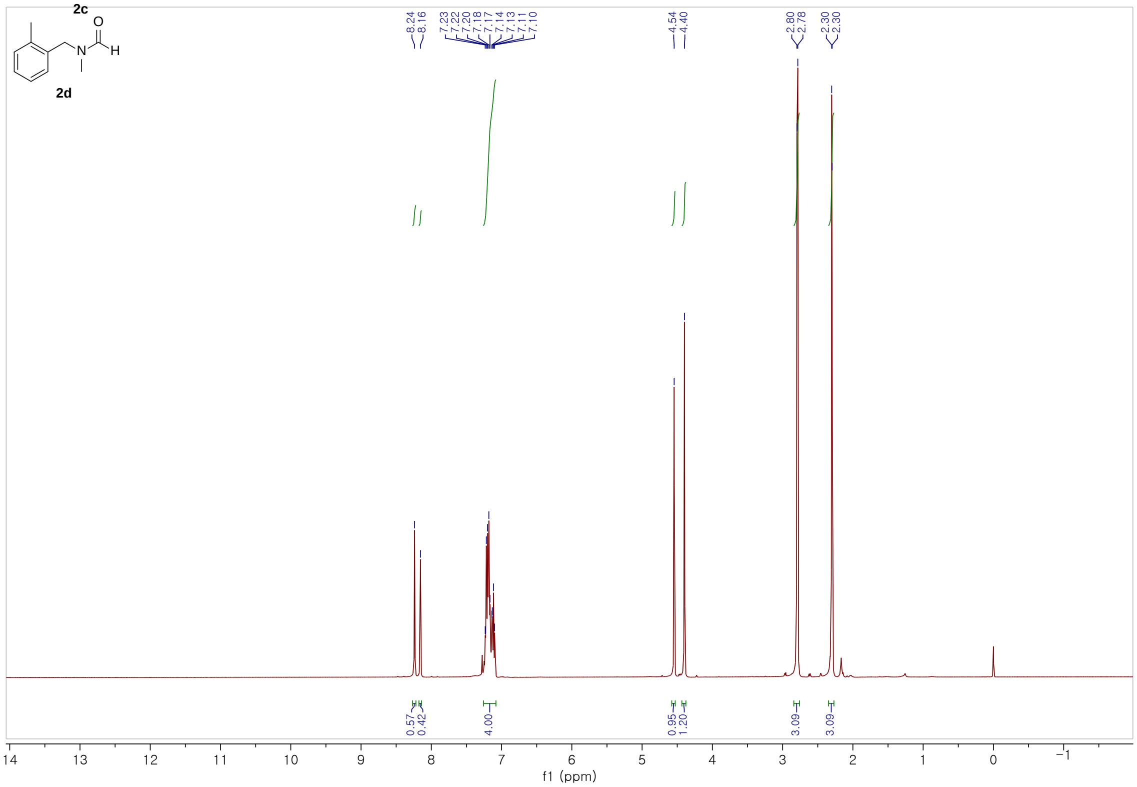
2d
8.24
8.16
7.23
7.22
7.20
7.18
7.17
7.14
7.13
7.11
7.10
4.54
4.40
2.80
2.78
2.30
2.30
0.57
0.42
4.00
0.95
1.20
3.09
3.09
14 13 12 11 10 9 8 7 6 5 4 3 2 1 0 −1
f1 (ppm)

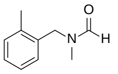

2d

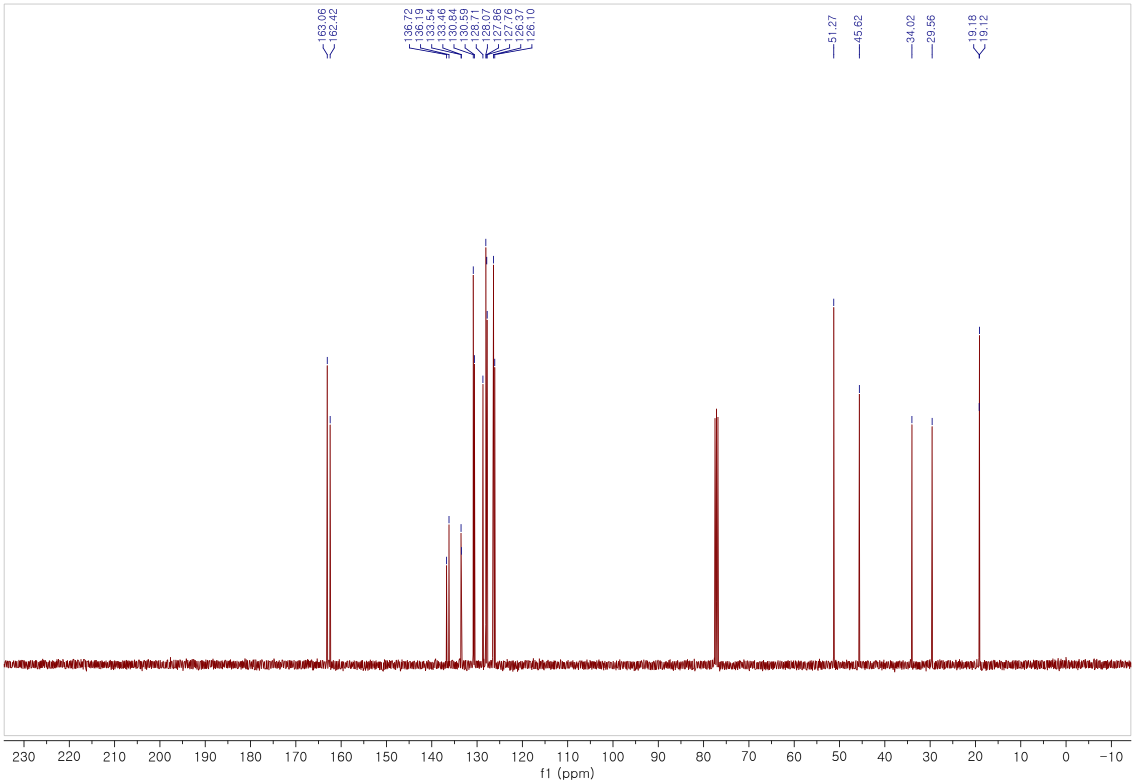
163.06
162.42
136.72
136.19
133.54
133.46
130.84
130.59
128.71
128.07
127.86
127.76
126.37
126.10
51.27
45.62
34.02
29.56
19.18
19.12
230 220 210 200 190 180 170 160 150 140 130 120 110 100 90 80 70 60 50 40 30 20 10 0 −10
f1 (ppm)

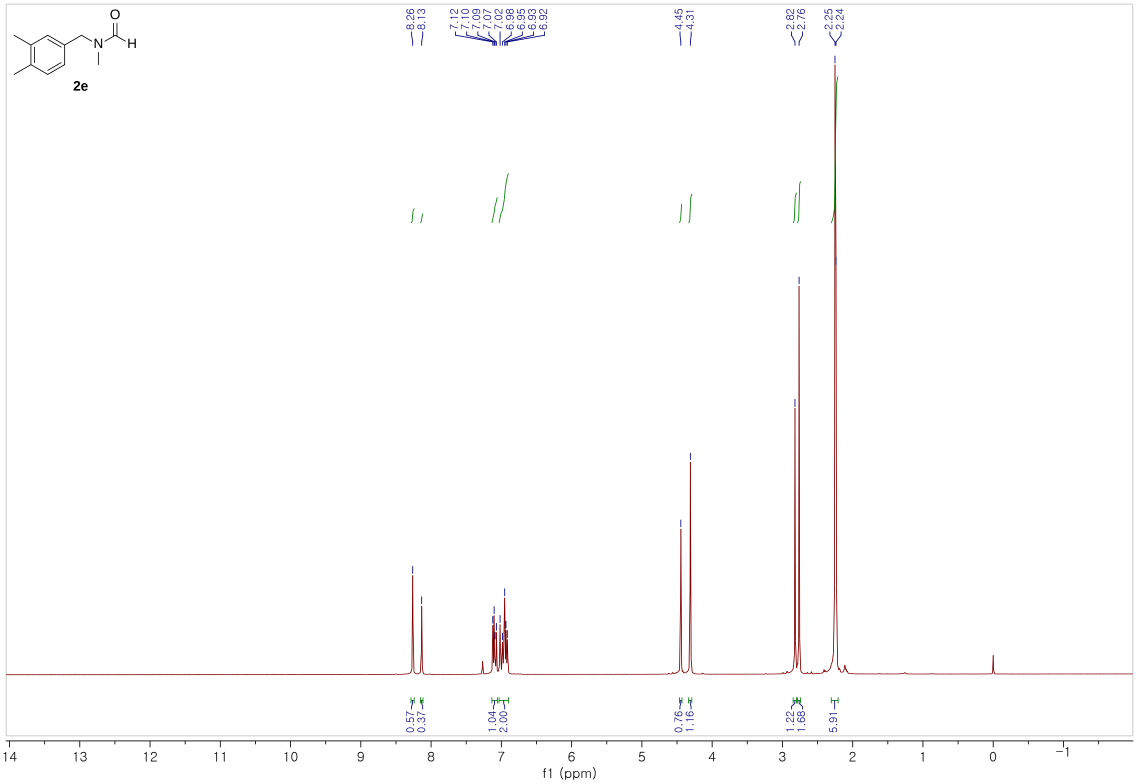
O
N
H
2e
8.26
8.13
7.12
7.10
7.09
7.07
7.02
6.98
6.95
6.93
6.92
4.45
4.31
2.82
2.76
2.25
2.24
0.57
0.37
1.04
2.00
0.76
1.16
1.22
1.68
5.91
14 13 12 11 10 9 8 7 6 5 4 3 2 1 0 −1
f1 (ppm)

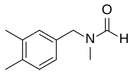
O
N
H
2e

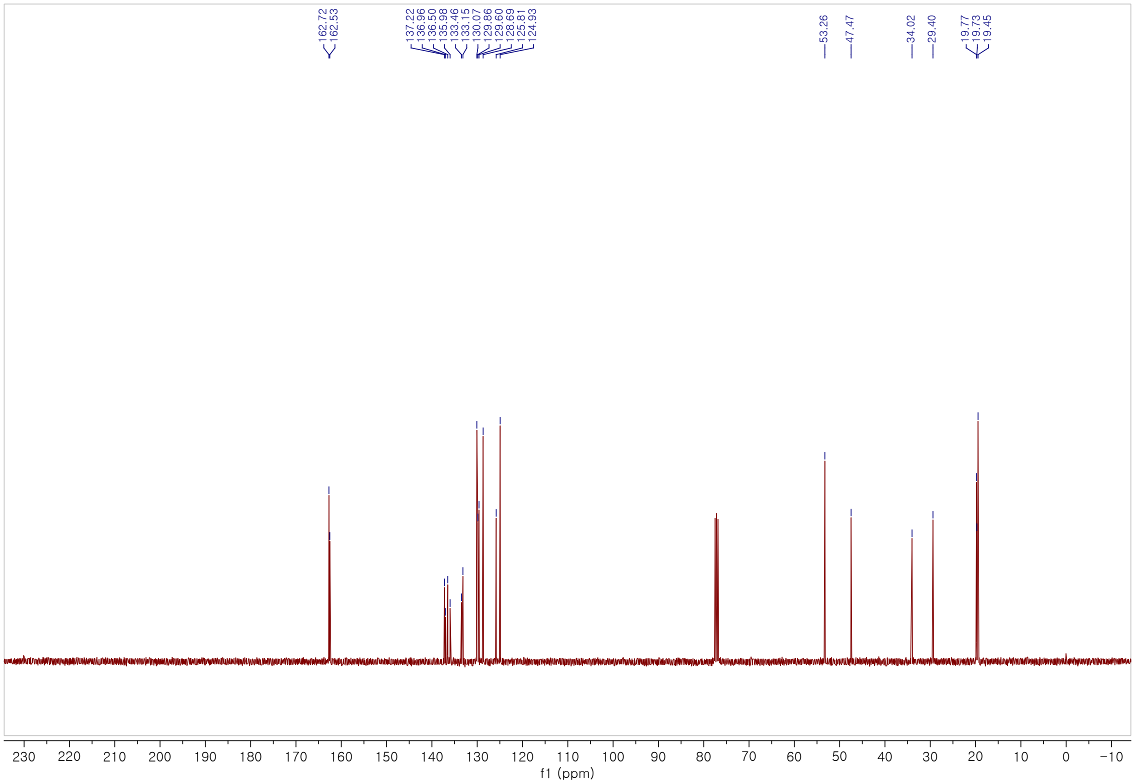
162.72
162.53
137.22
136.96
136.50
135.98
133.46
133.15
130.07
129.86
129.60
128.69
125.81
124.93
53.26
47.47
34.02
29.40
19.77
19.73
19.45
230 220 210 200 190 180 170 160 150 140 130 120 110 100 90 80 70 60 50 40 30 20 10 0 −10
f1 (ppm)

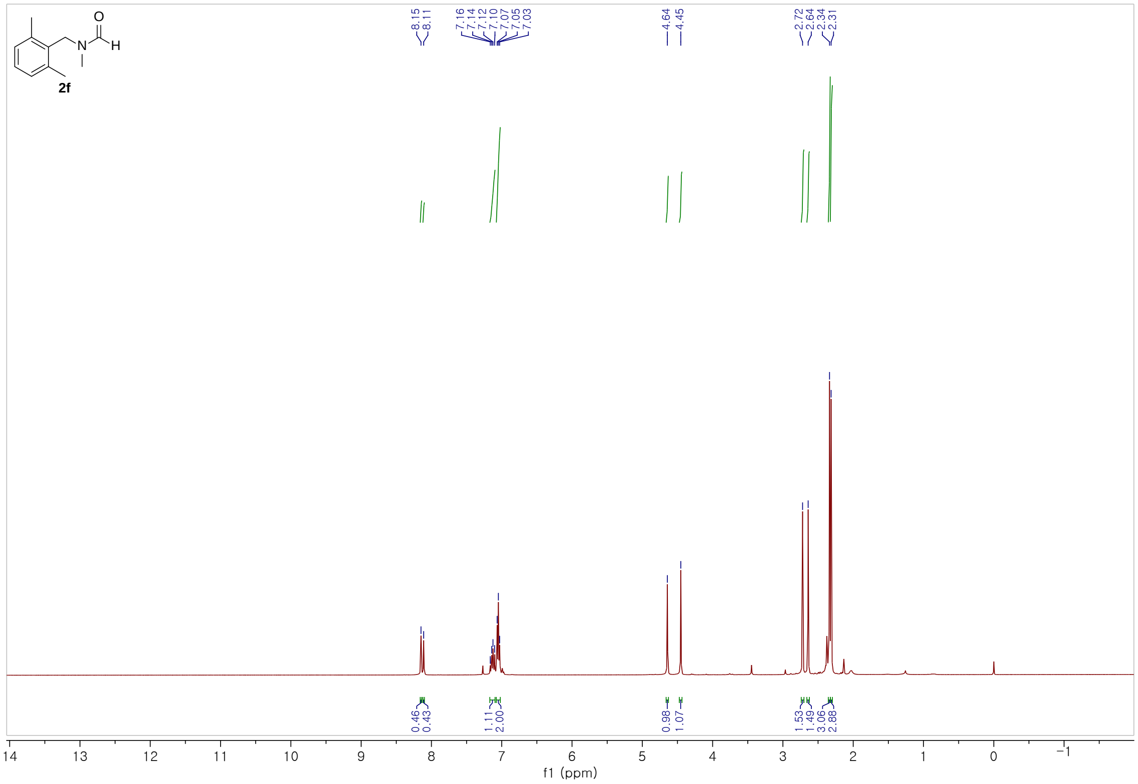
O
N
H
2f
8.15
8.11
7.16
7.14
7.12
7.10
7.07
7.05
7.03
4.64
4.45
2.72
2.64
2.34
2.31
0.46
0.43
1.11
2.00
0.98
1.07
1.53
1.49
3.06
2.88
14 13 12 11 10 9 8 7 6 5 4 3 2 1 0 −1
f1 (ppm)

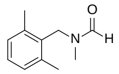
O
N
H
2f

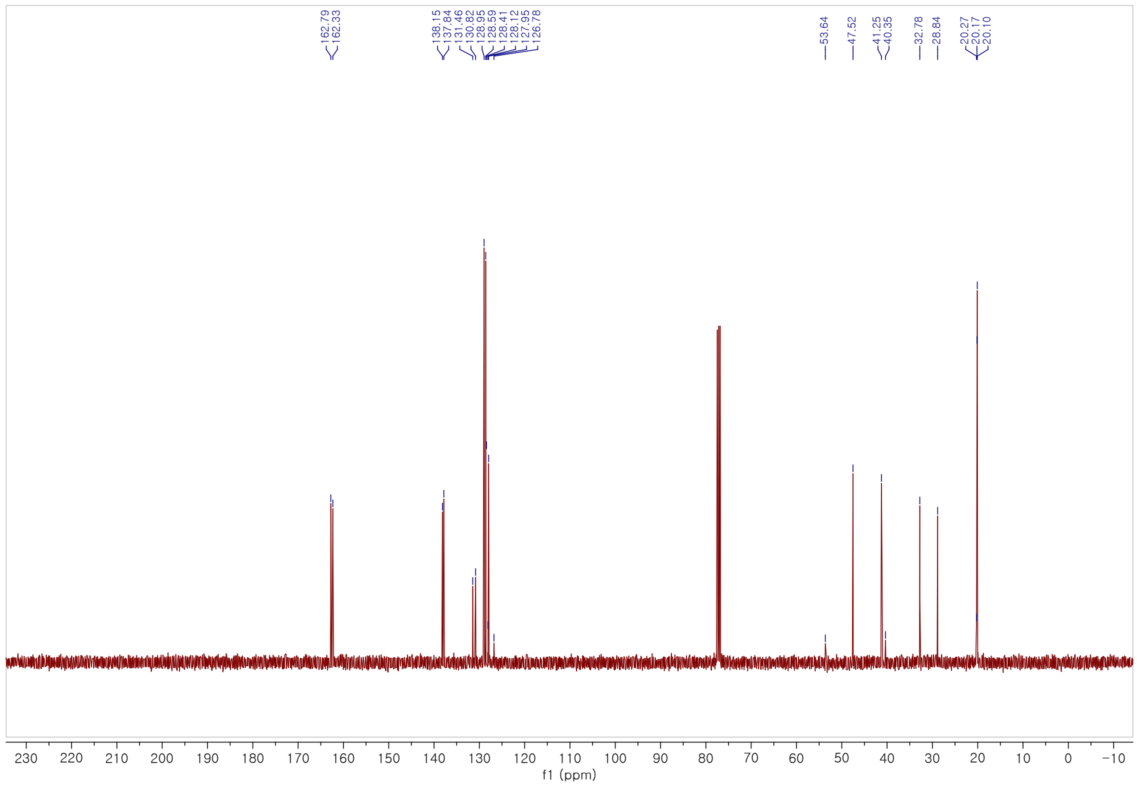
162.79
162.33
138.15
137.84
131.46
130.82
128.95
128.59
128.41
128.12
127.95
126.78
53.64
47.52
41.25
40.35
32.78
28.84
20.27
20.17
20.10
f1 (ppm)

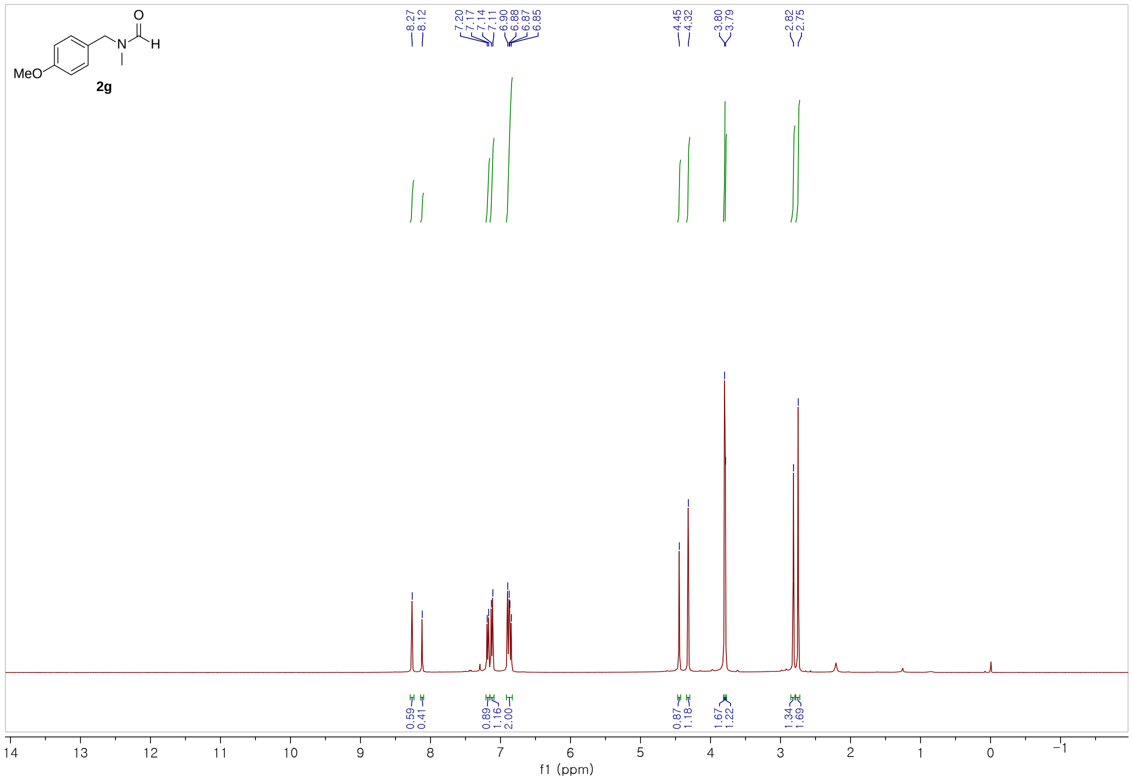
MeO
N
O
H
2g
8.27
8.12
7.20
7.17
7.14
7.11
6.90
6.88
6.87
6.85
4.45
4.32
3.80
3.79
2.82
2.75
0.59
0.41
0.89
1.16
2.00
0.87
1.18
1.67
1.22
1.34
1.69
f1 (ppm)

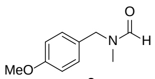
MeO
N
O
H

2g

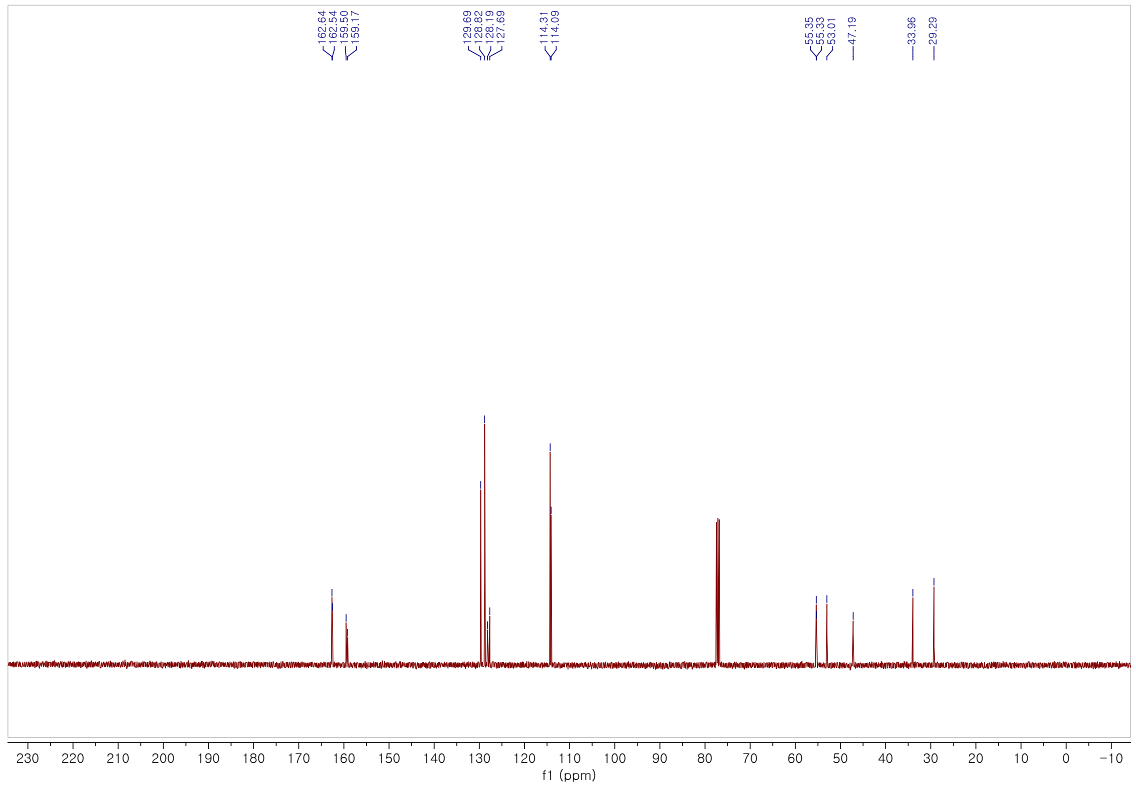
162.64
162.54
159.50
159.17
129.69
128.82
128.19
127.69
114.31
114.09
55.35
55.33
53.01
47.19
33.96
29.29
230 220 210 200 190 180 170 160 150 140 130 120 110 100 90 80 70 60 50 40 30 20 10 0 −10
f1 (ppm)

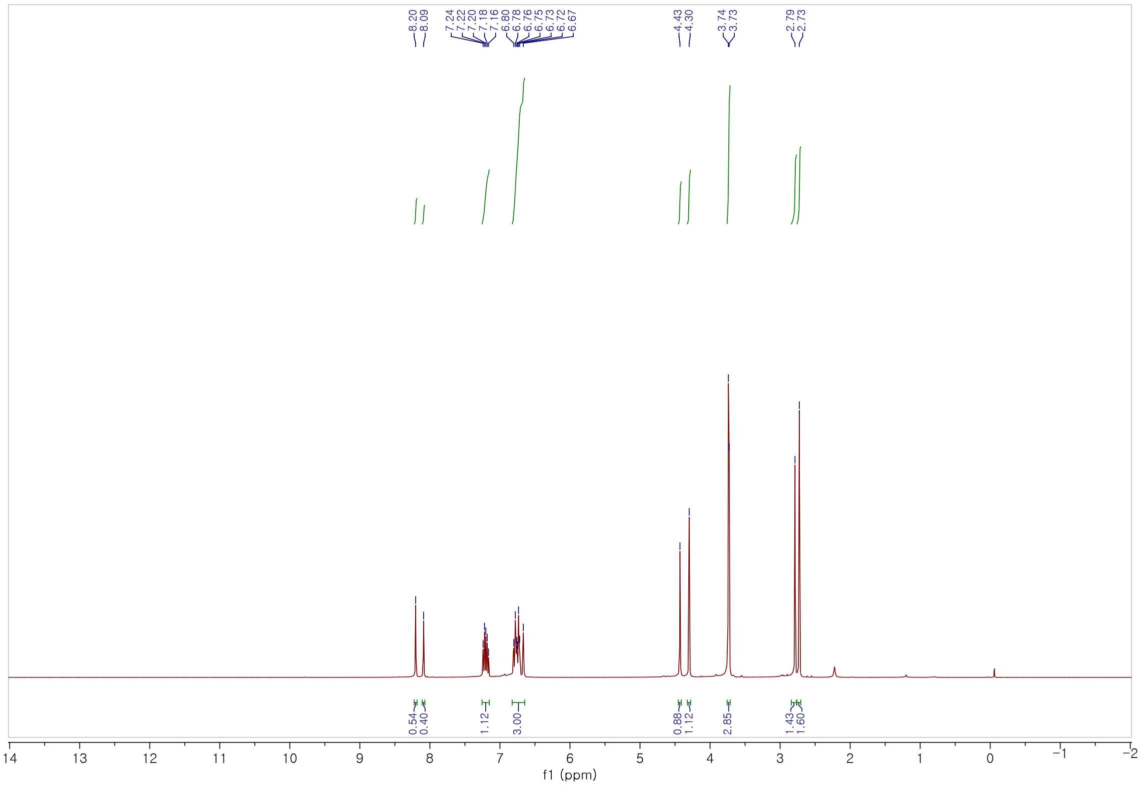
8.20
8.09
7.24
7.22
7.20
7.18
7.16
6.80
6.78
6.76
6.75
6.73
6.72
6.67
4.43
4.30
3.74
3.73
2.79
2.73
0.54
0.40
1.12
3.00
0.88
1.12
2.85
1.43
1.60
14 13 12 11 10 9 8 7 6 5 4 3 2 1 0 −1 −2
f1 (ppm)

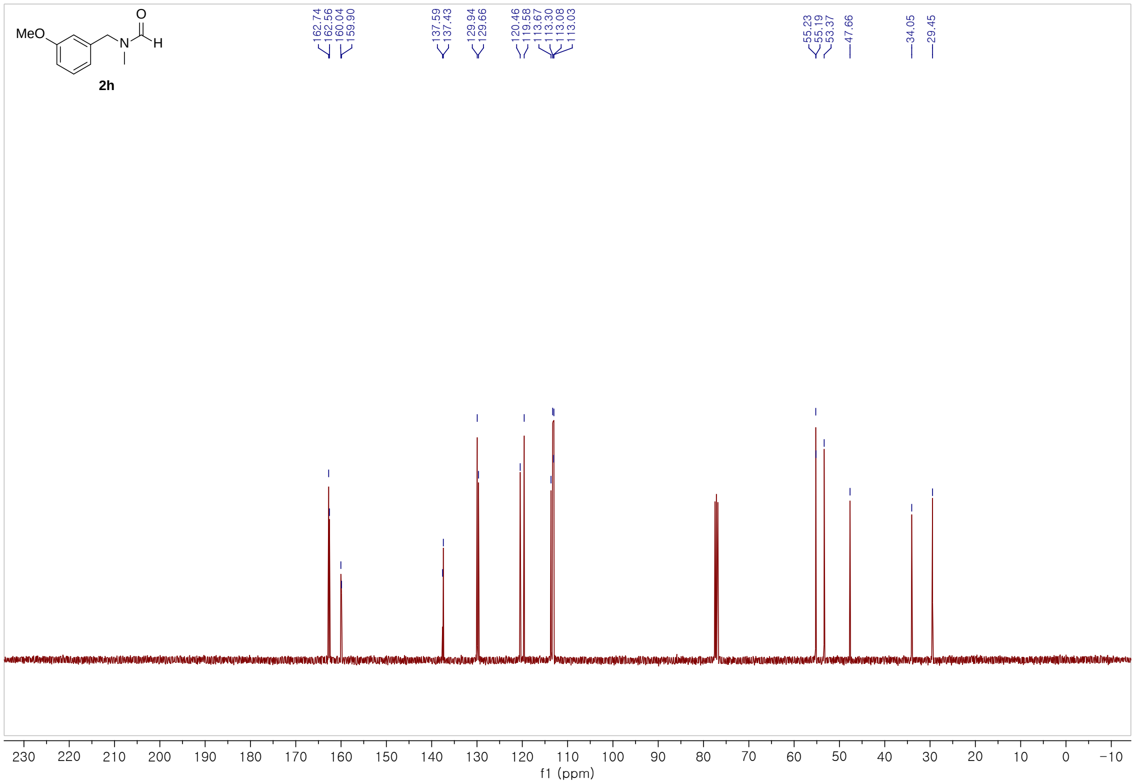
MeO
O
N
H
2h
162.74
162.56
160.04
159.90
137.59
137.43
129.94
129.66
120.46
119.58
113.67
113.30
113.08
113.03
55.23
55.19
53.37
47.66
34.05
29.45
230 220 210 200 190 180 170 160 150 140 130 120 110 100 90 80 70 60 50 40 30 20 10 0 −10
f1 (ppm)

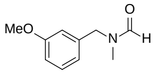
MeO
O
N
H

2h

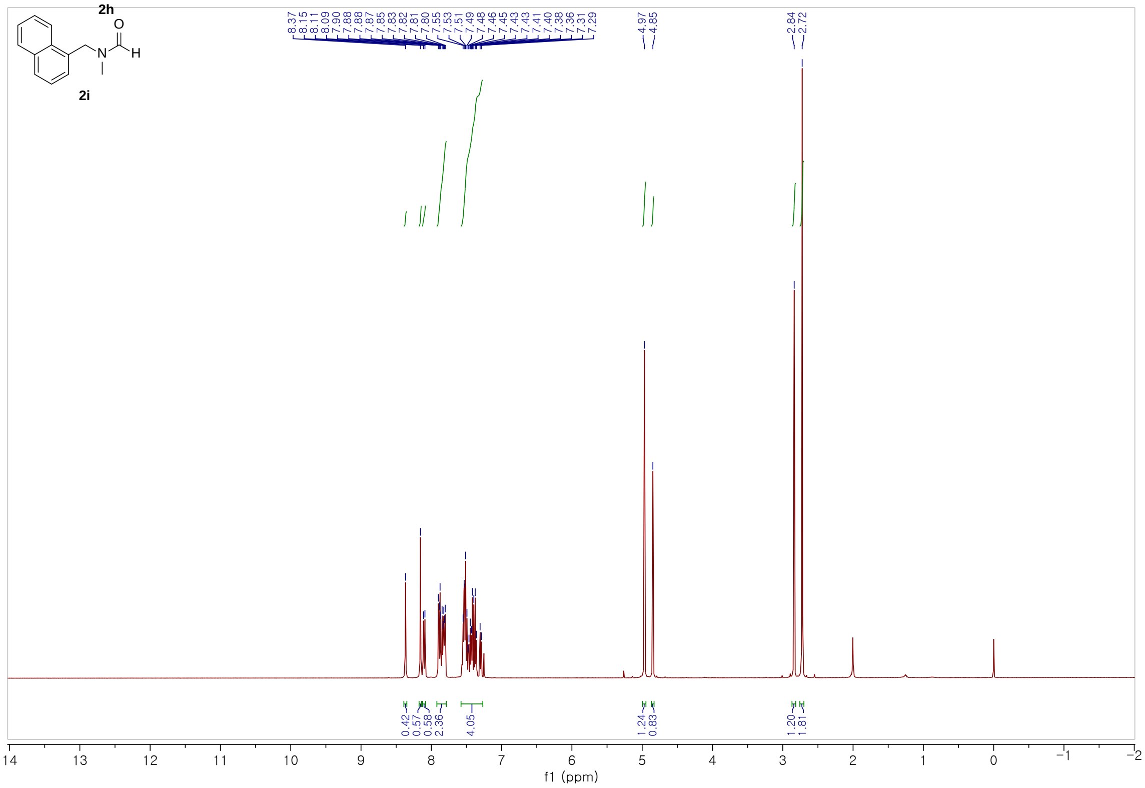
O
N
H
2i
8.37
8.15
8.11
8.09
7.90
7.88
7.88
7.87
7.85
7.83
7.82
7.81
7.80
7.55
7.53
7.51
7.49
7.48
7.46
7.45
7.43
7.43
7.41
7.40
7.38
7.36
7.31
7.29
4.97
4.85
2.84
2.72
0.42
0.57
0.58
2.36
4.05
1.24
0.83
1.20
1.81
14 13 12 11 10 9 8 7 6 5 4 3 2 1 0 −1 −2
f1 (ppm)

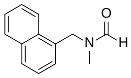
O
N
H

2i

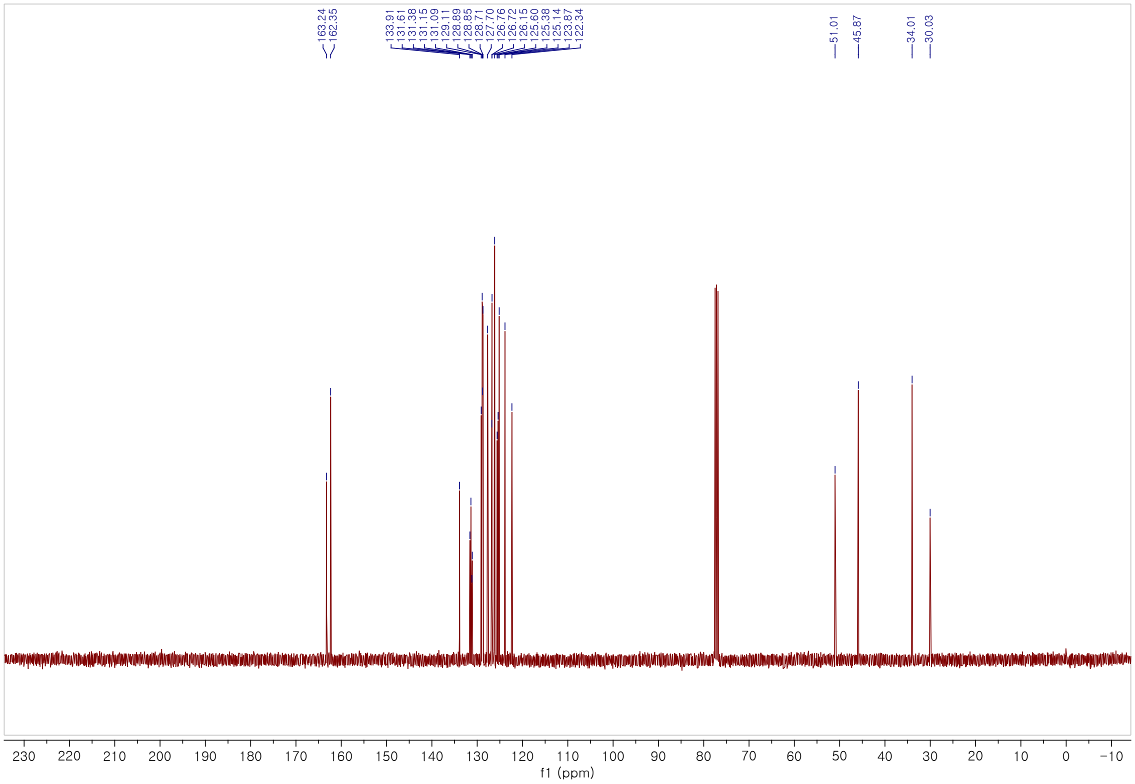
163.24
162.35
133.91
131.61
131.38
131.15
131.09
129.11
128.89
128.85
128.71
127.70
126.76
126.72
126.15
125.60
125.38
125.14
123.87
122.34
51.01
45.87
34.01
30.03
f1 (ppm)

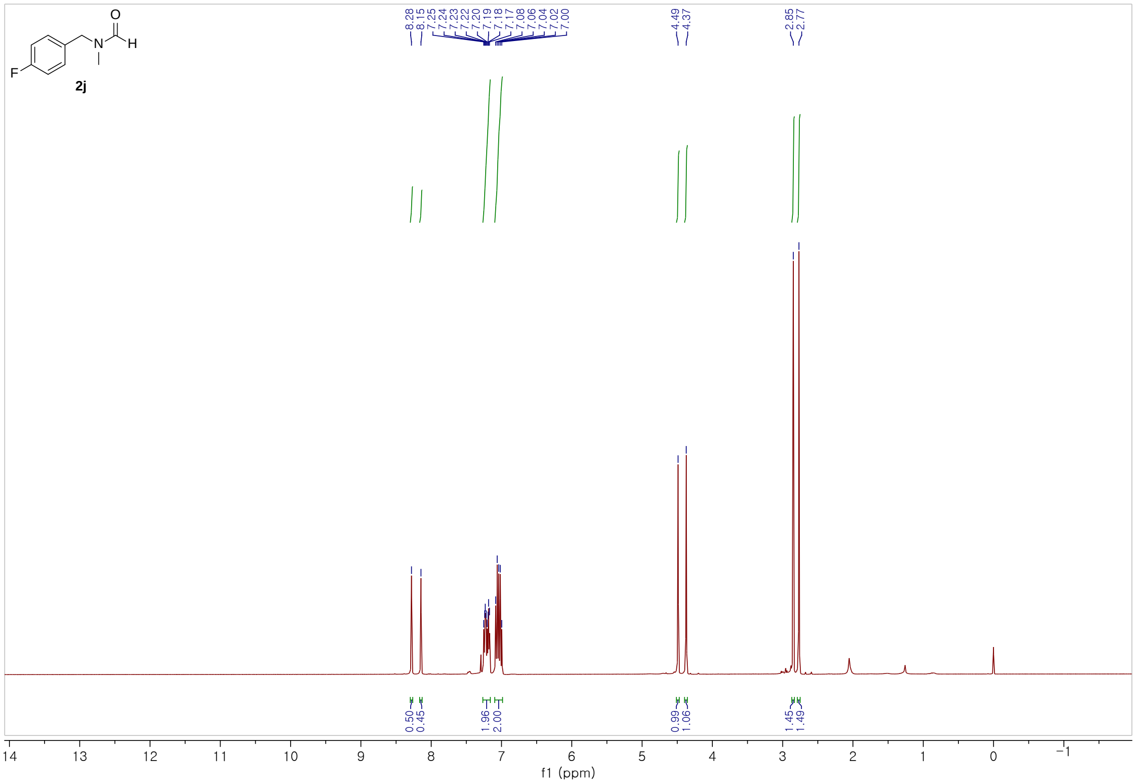
2j
8.28
8.15
7.25
7.24
7.23
7.22
7.20
7.19
7.18
7.17
7.08
7.06
7.04
7.02
7.00
4.49
4.37
2.85
2.77
0.50
0.45
1.96
2.00
0.99
1.06
1.45
1.49
f1 (ppm)

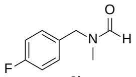

2j

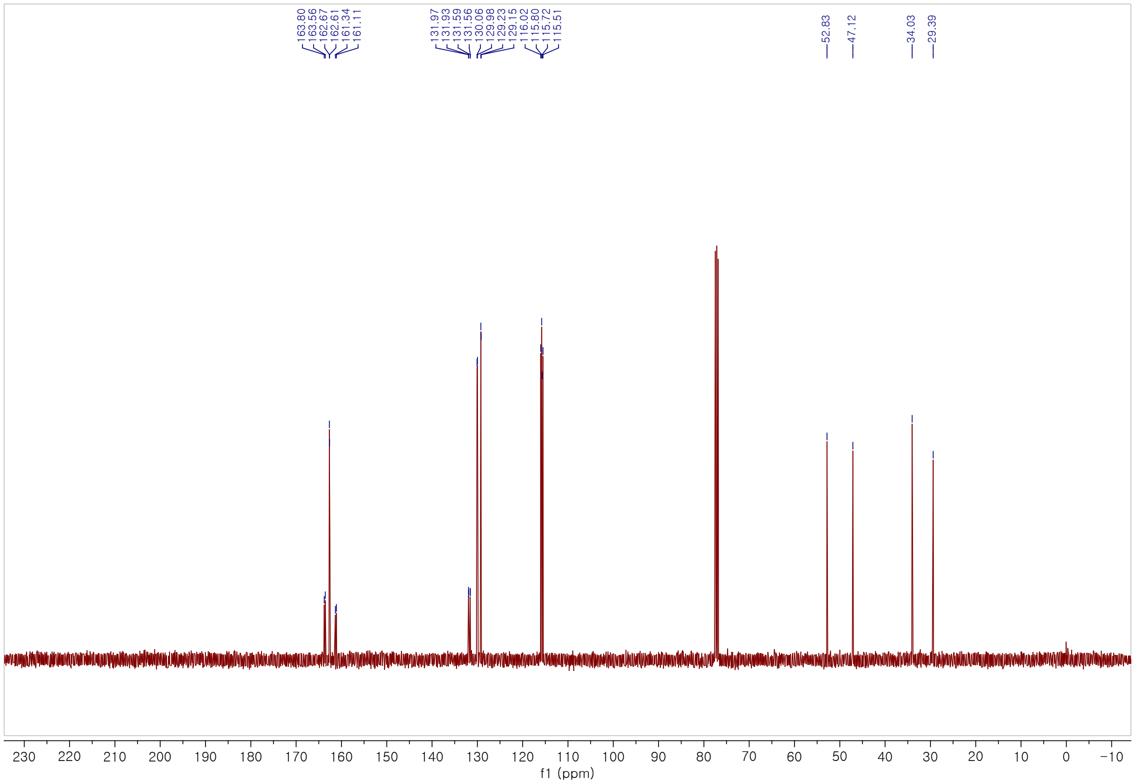

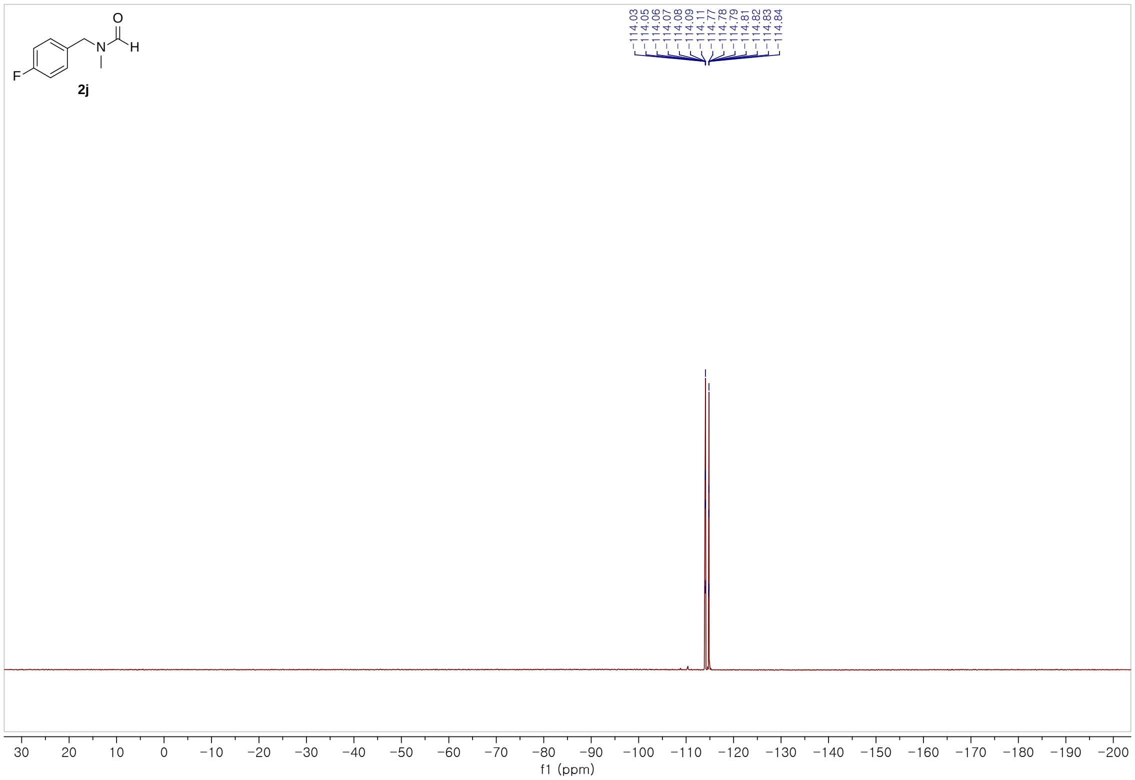

2j

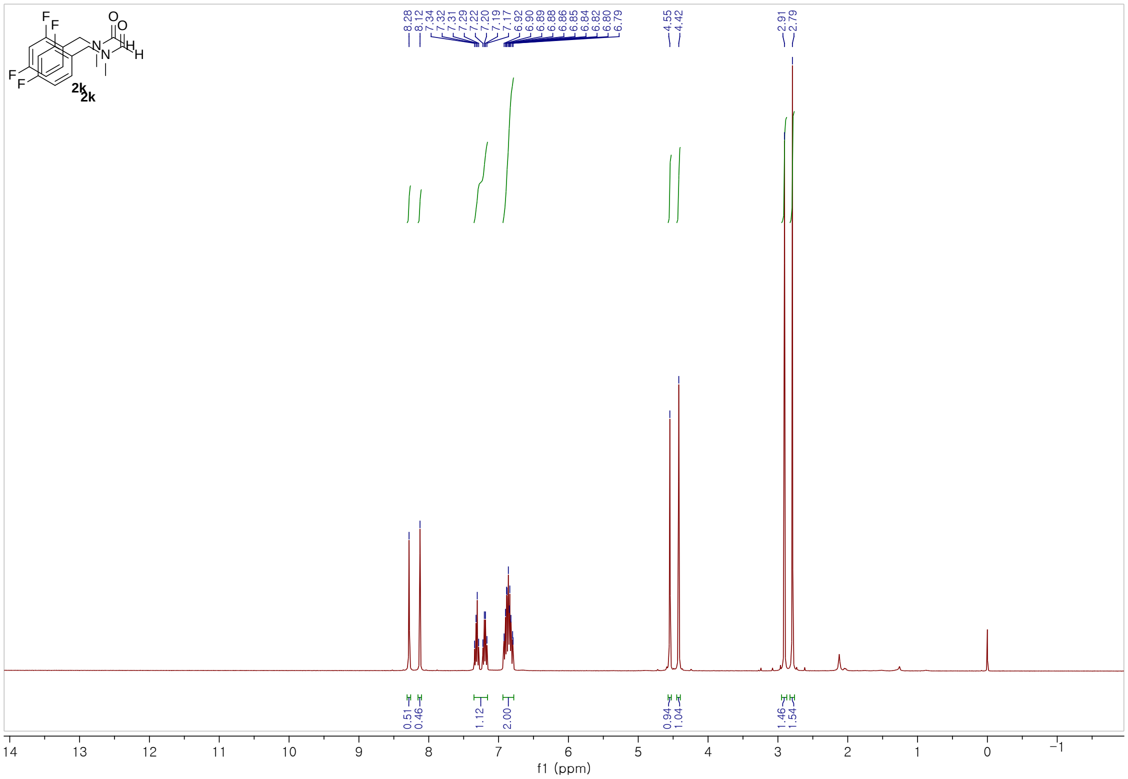

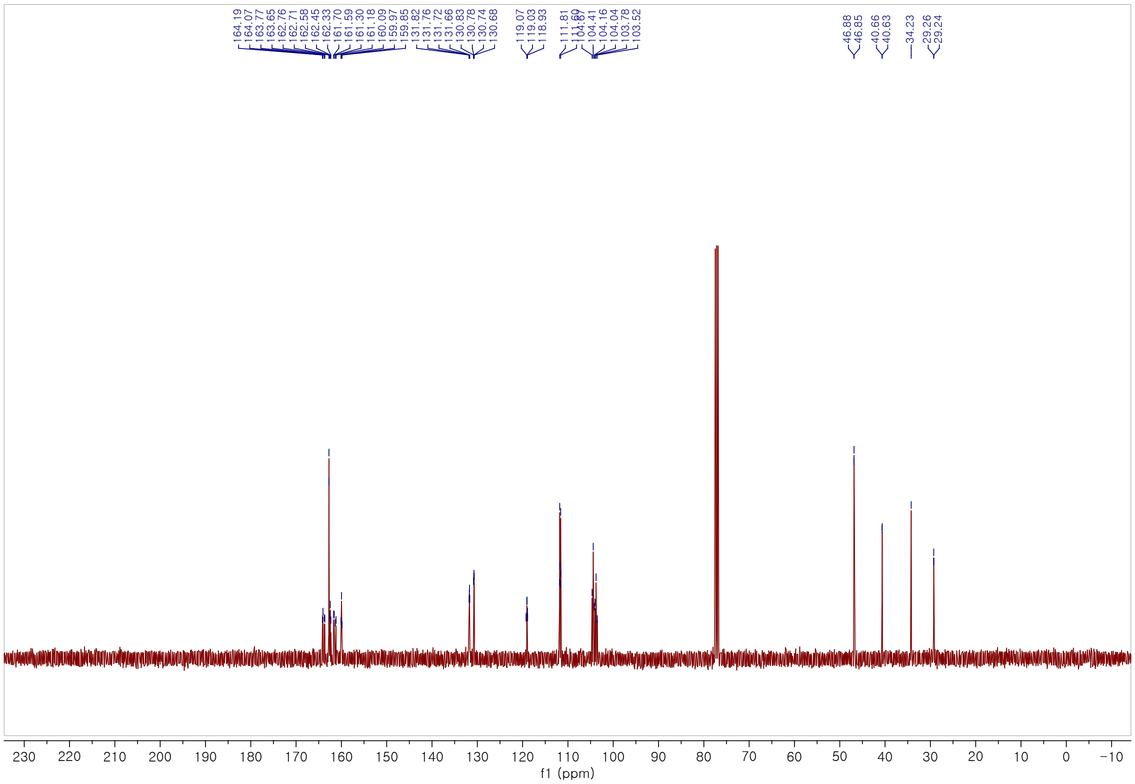

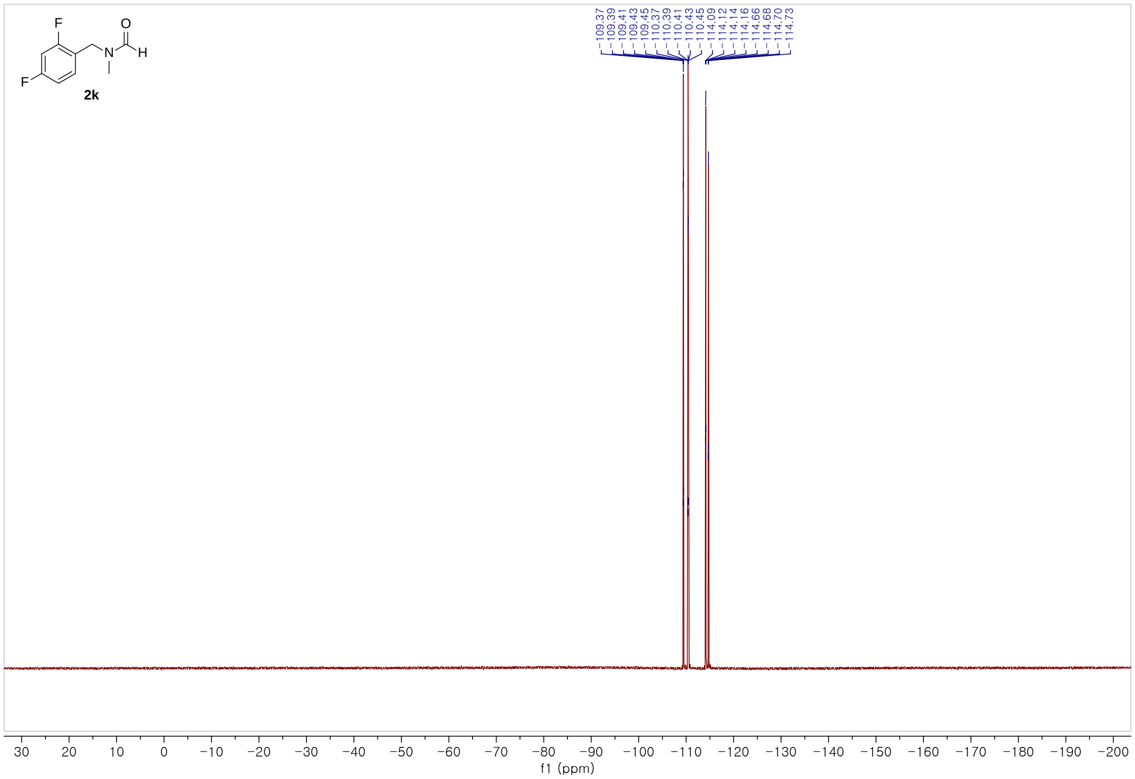
F
O
N
H
F
2k
−109.37
−109.39
−109.41
−109.43
−109.45
−110.37
−110.39
−110.41
−110.43
−110.45
−114.09
−114.12
−114.14
−114.16
−114.66
−114.68
−114.70
−114.73
30 20 10 0 −10 −20 −30 −40 −50 −60 −70 −80 −90 −100 −110 −120 −130 −140 −150 −160 −170 −180 −190 −200
f1 (ppm)

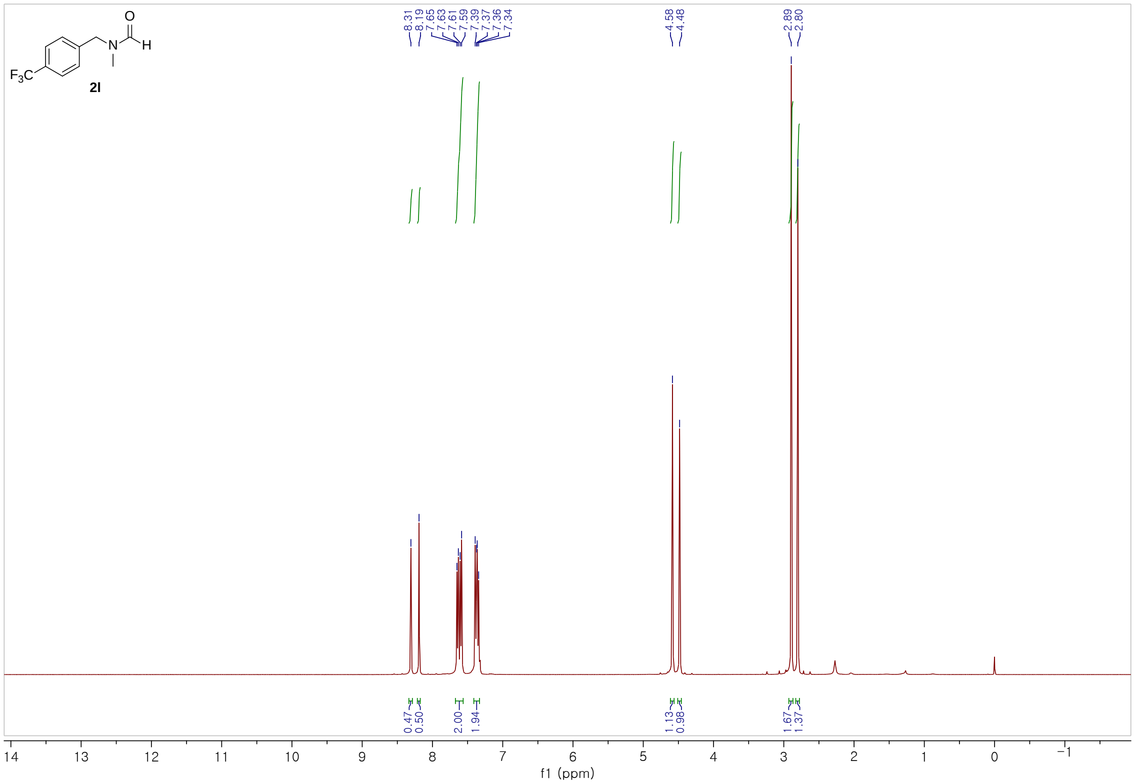
O
N
H
F_3C
2l
8.31
8.19
7.65
7.63
7.61
7.59
7.39
7.37
7.36
7.34
4.58
4.48
2.89
2.80
0.47
0.50
2.00
1.94
1.13
0.98
1.67
1.37
14 13 12 11 10 9 8 7 6 5 4 3 2 1 0 −1
f1 (ppm)

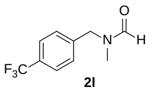
O
N
H
F_3C
2l

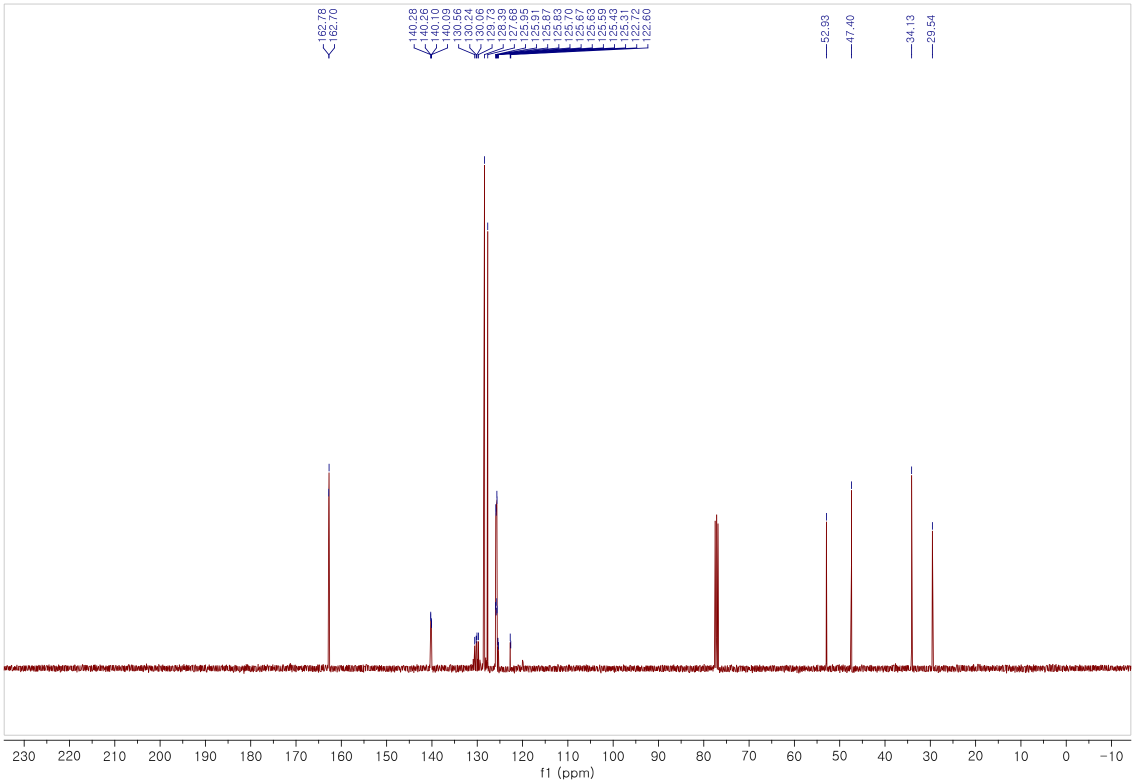
162.78
162.70
140.28
140.26
140.10
140.09
130.56
130.24
130.06
129.73
128.39
127.68
125.95
125.91
125.87
125.83
125.70
125.67
125.63
125.59
125.43
125.31
122.72
122.60
52.93
47.40
34.13
29.54
f1 (ppm)

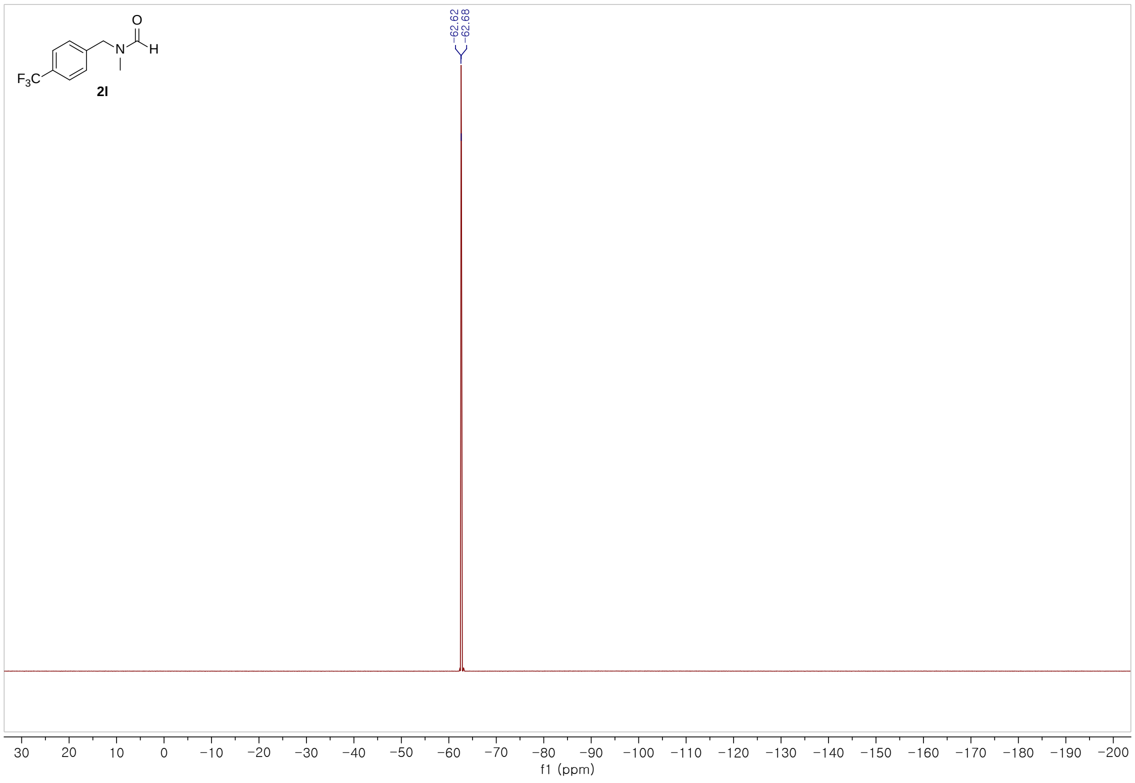
2l
-62.62
-62.68
f1 (ppm)

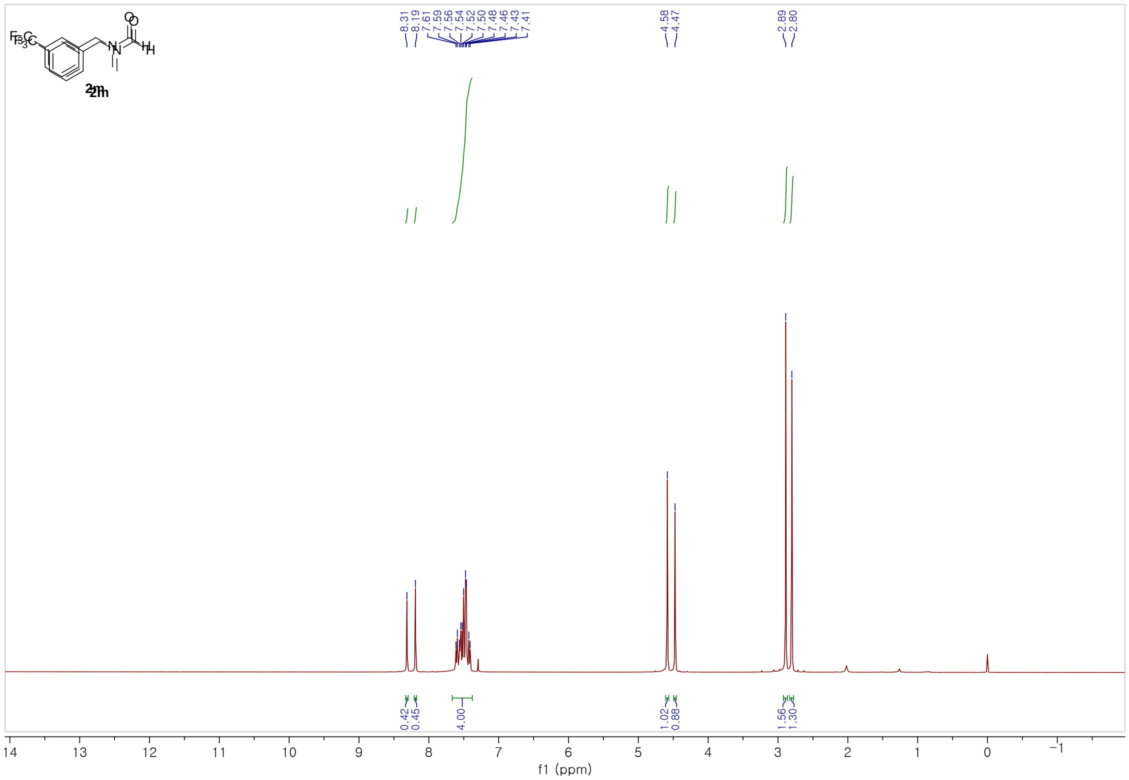
2m
8.31
8.19
7.61
7.59
7.56
7.54
7.52
7.50
7.48
7.46
7.43
7.41
4.58
4.47
2.89
2.80
0.42
0.45
4.00
1.02
0.88
1.56
1.30
f1 (ppm)

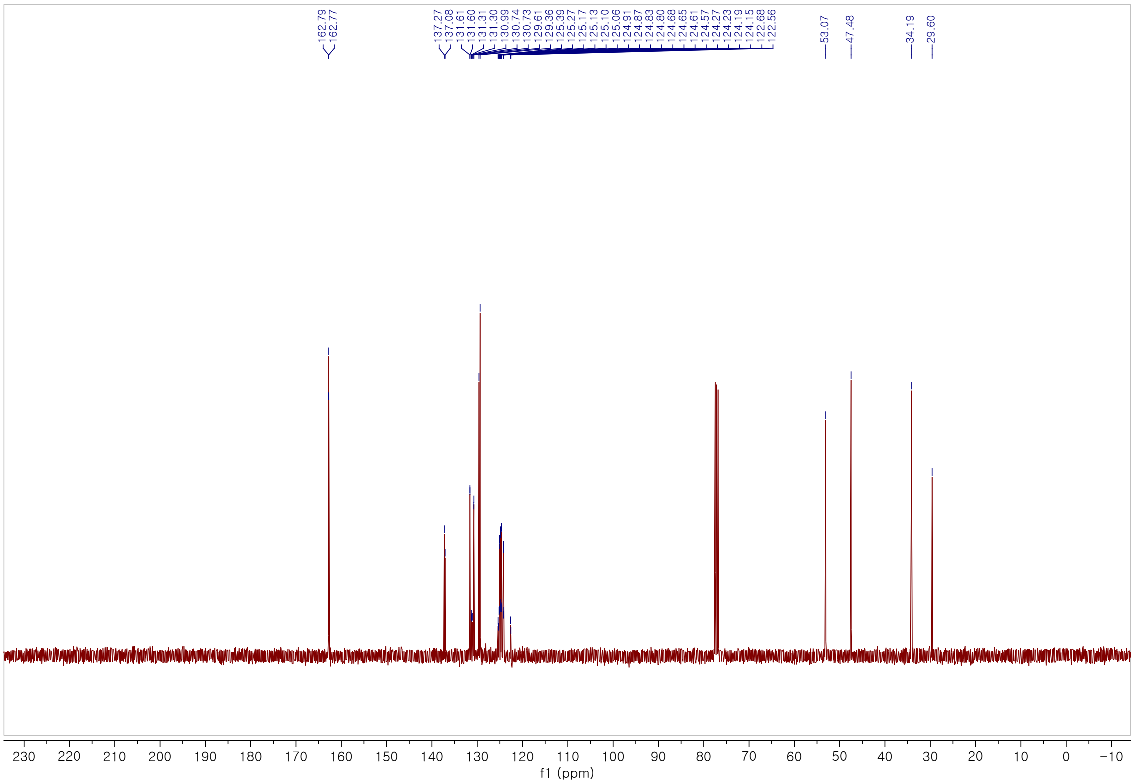
162.79
162.77
137.27
137.08
131.61
131.60
131.31
131.30
130.99
130.74
130.73
129.61
129.36
125.39
125.27
125.17
125.13
125.10
125.06
124.91
124.87
124.83
124.80
124.68
124.65
124.61
124.57
124.27
124.23
124.19
124.15
122.68
122.56
53.07
47.48
34.19
29.60
f1 (ppm)

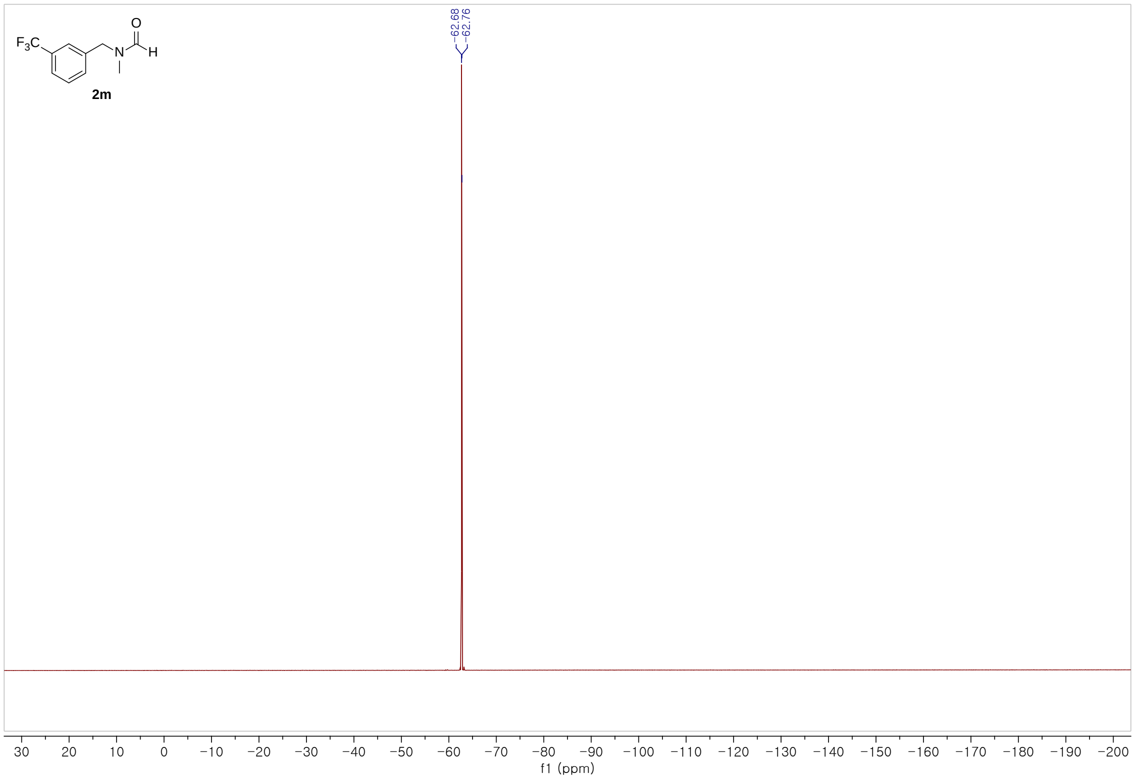

F3C
O
N
H
2m
-62.68
-62.76
30 20 10 0 −10 −20 −30 −40 −50 −60 −70 −80 −90 −100 −110 −120 −130 −140 −150 −160 −170 −180 −190 −200
f1 (ppm)

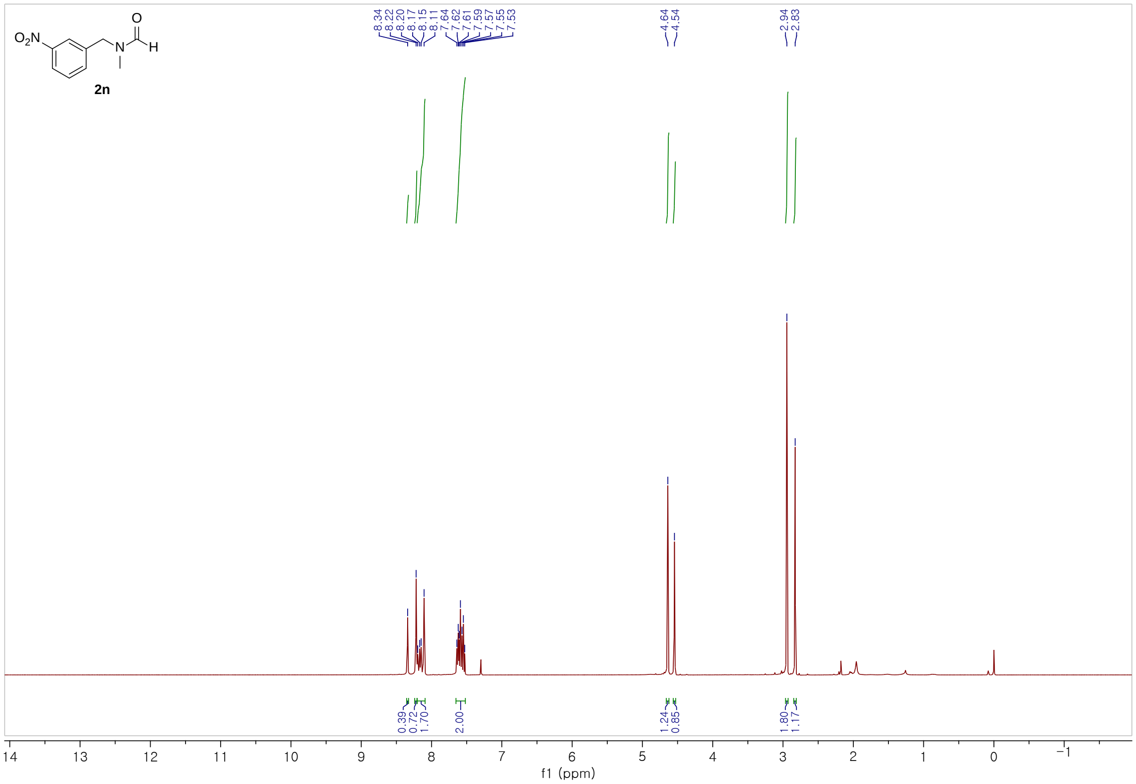

O2N
O
N
H
2n
8.34
8.22
8.20
8.17
8.15
8.11
7.64
7.62
7.61
7.59
7.57
7.55
7.53
4.64
4.54
2.94
2.83
0.39
0.72
1.70
2.00
1.24
0.85
1.80
1.17
14 13 12 11 10 9 8 7 6 5 4 3 2 1 0 −1
f1 (ppm)

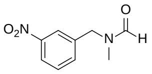

O2N
O
N
H

2n

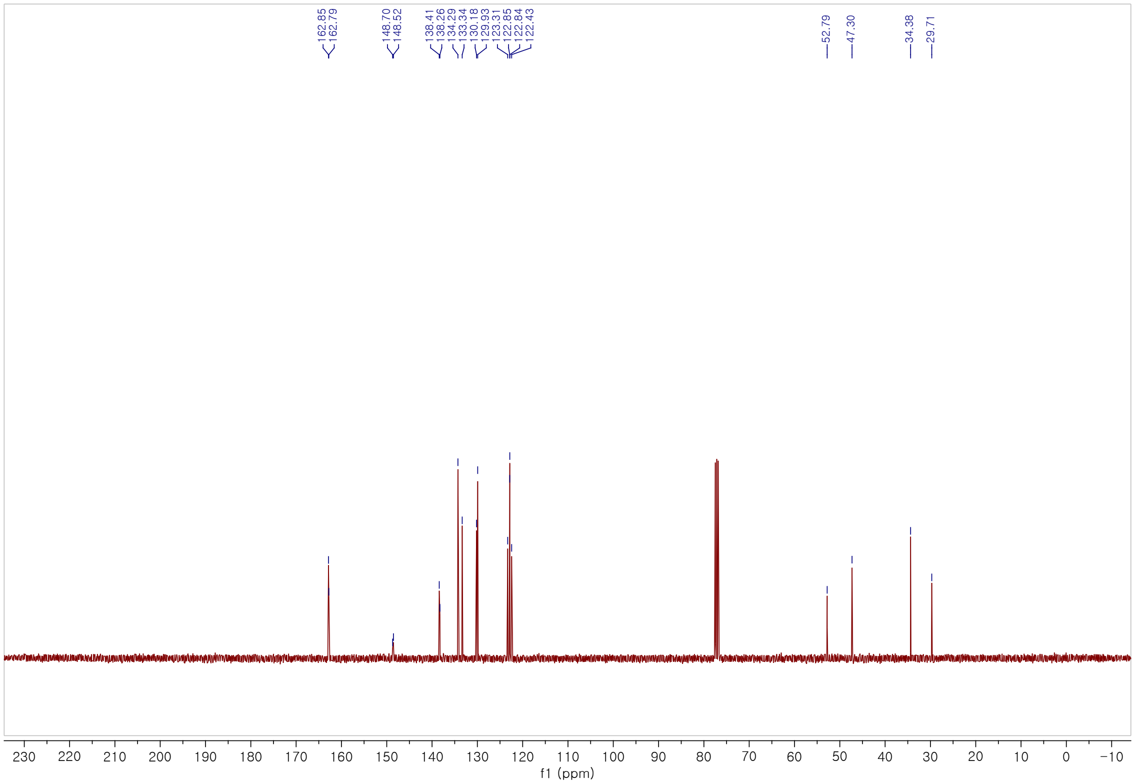
162.85
162.79
148.70
148.52
138.41
138.26
134.29
133.34
130.18
129.93
123.31
122.85
122.84
122.43
52.79
47.30
34.38
29.71
230 220 210 200 190 180 170 160 150 140 130 120 110 100 90 80 70 60 50 40 30 20 10 0 −10
f1 (ppm)

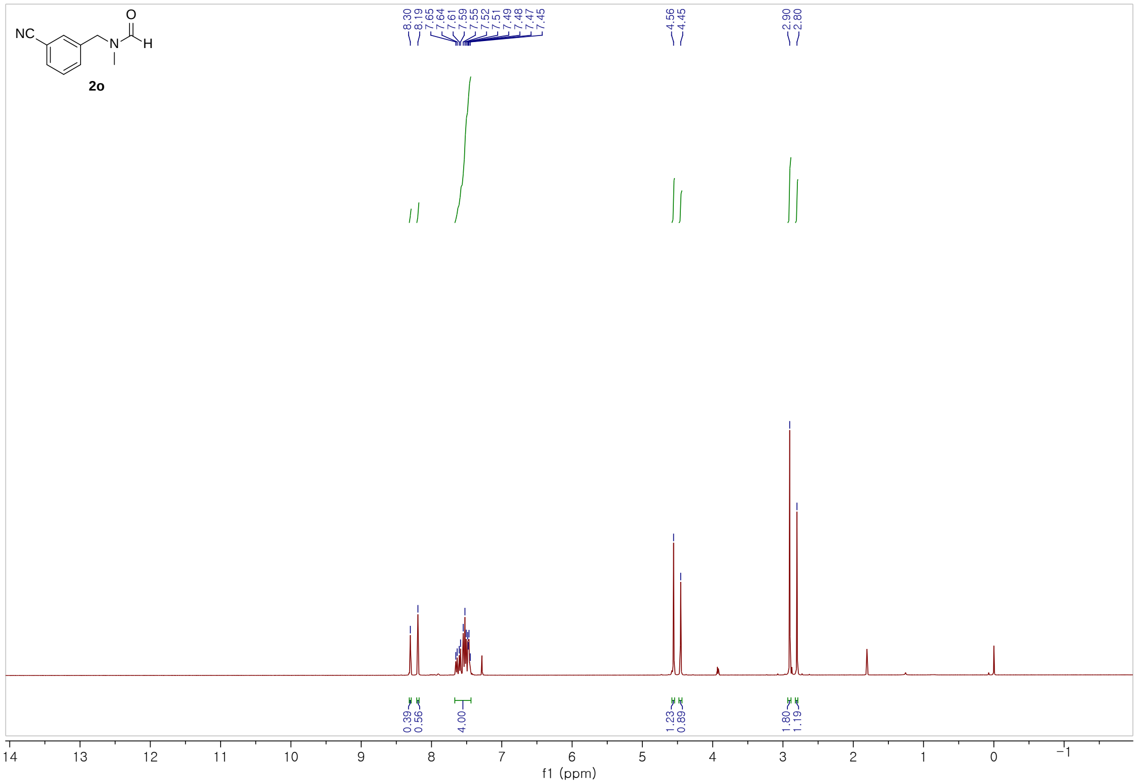
NC
O
N
H
2o
8.30
8.19
7.65
7.64
7.61
7.59
7.55
7.52
7.51
7.49
7.48
7.47
7.45
4.56
4.45
2.90
2.80
0.39
0.56
4.00
1.23
0.89
1.80
1.19
14 13 12 11 10 9 8 7 6 5 4 3 2 1 0 −1
f1 (ppm)

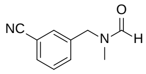
NC
O
N
H

2o

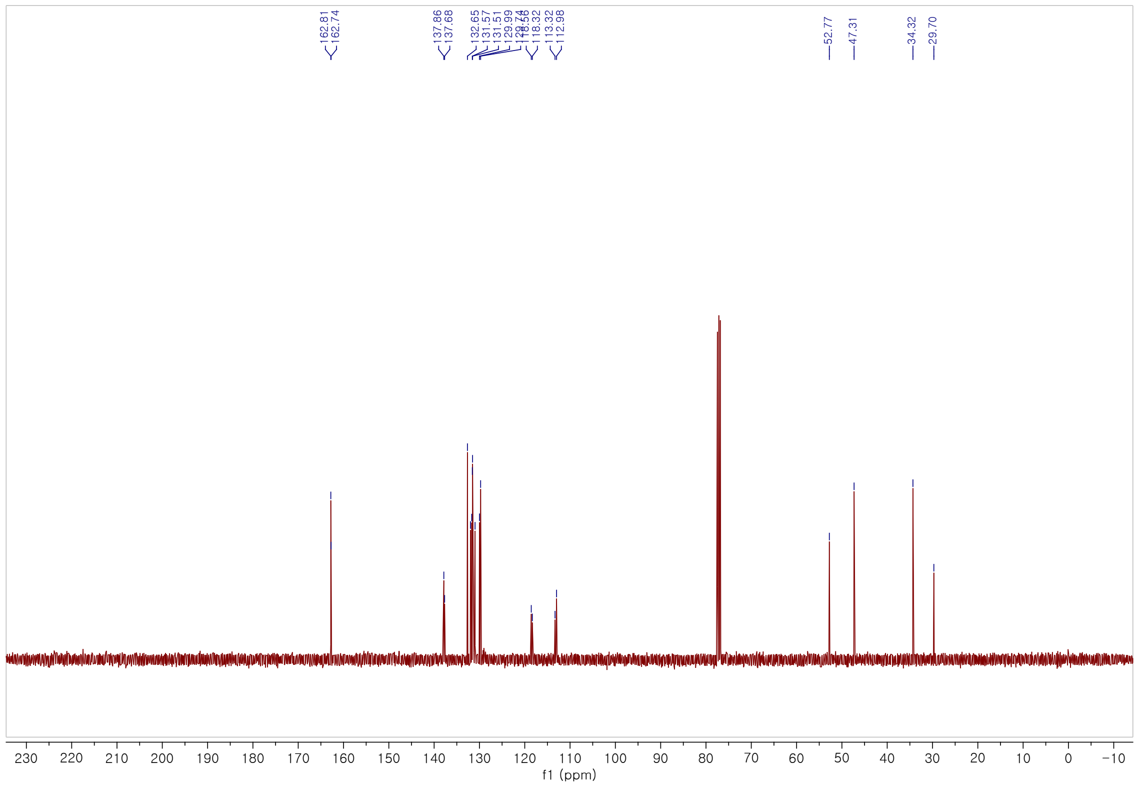
162.81
162.74
137.86
137.68
132.65
131.57
131.51
129.99
118.32
113.32
112.98
52.77
47.31
34.32
29.70
f1 (ppm)

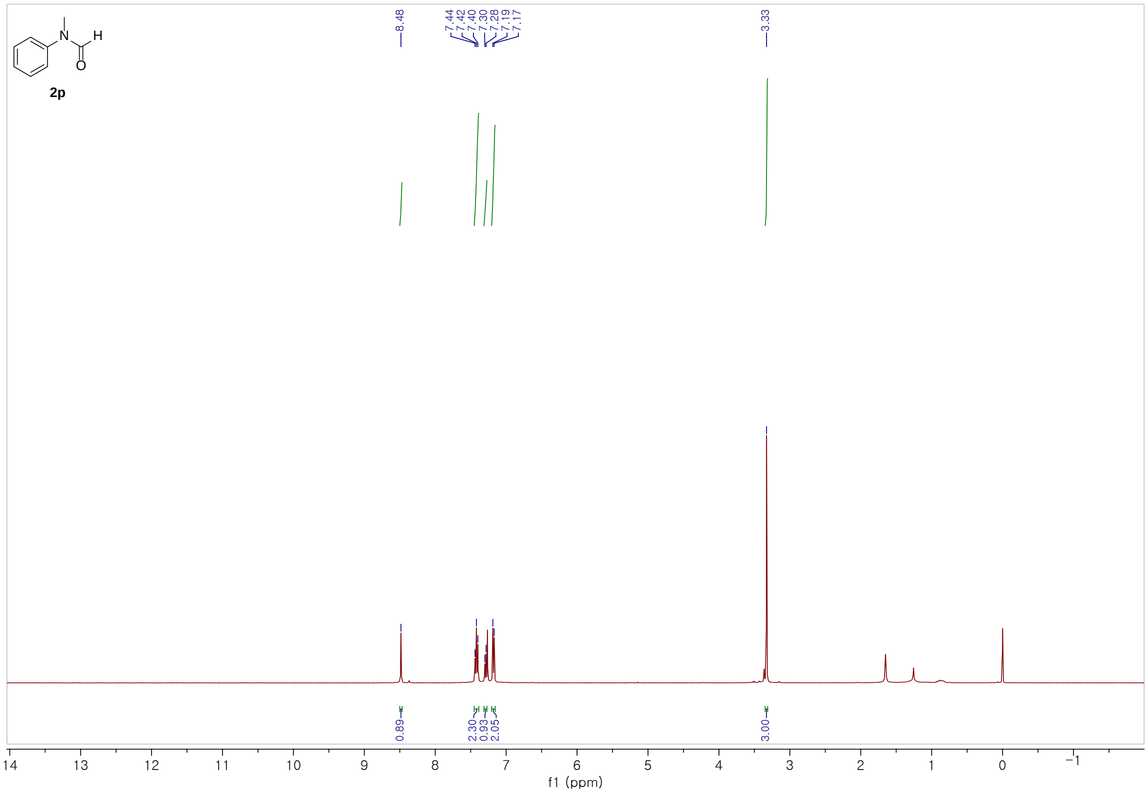
2p
8.48
7.44
7.42
7.40
7.30
7.28
7.19
7.17
3.33
0.89
2.30
0.93
2.05
3.00
f1 (ppm)

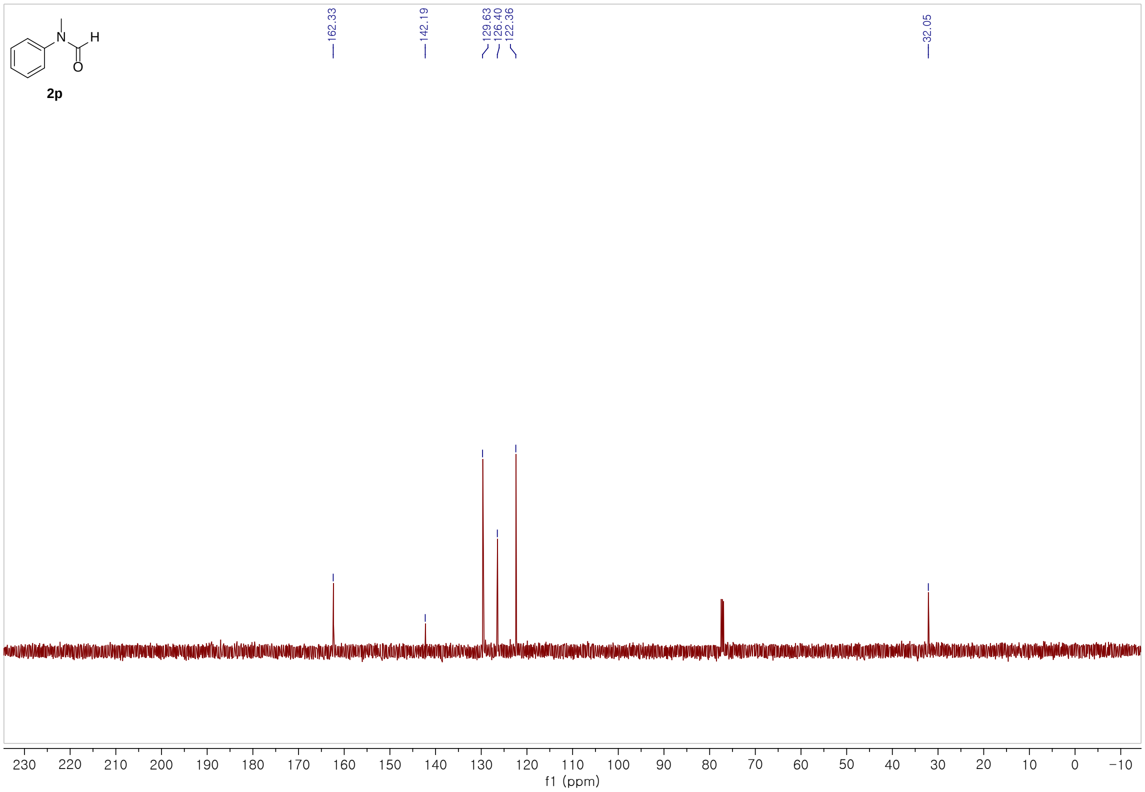

2p
162.33
142.19
129.63
126.40
122.36
32.05
f1 (ppm)

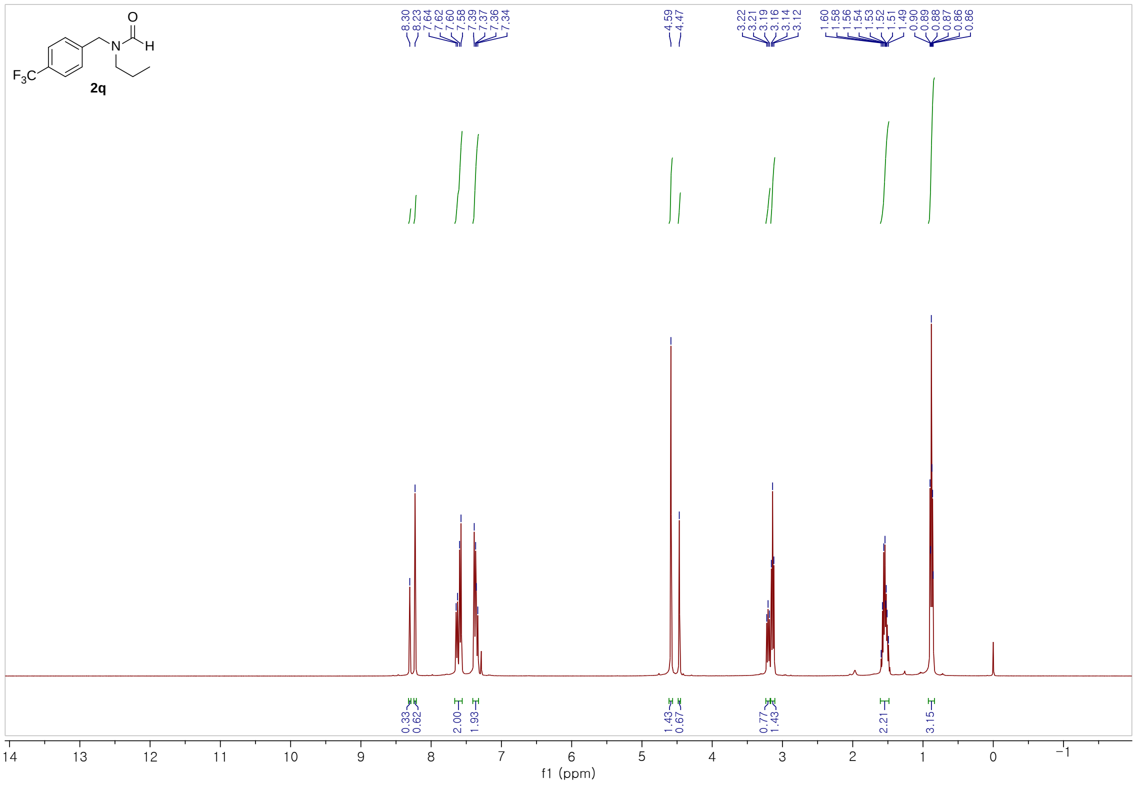

2q
F3C
f1 (ppm)

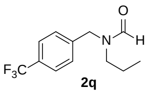

2q

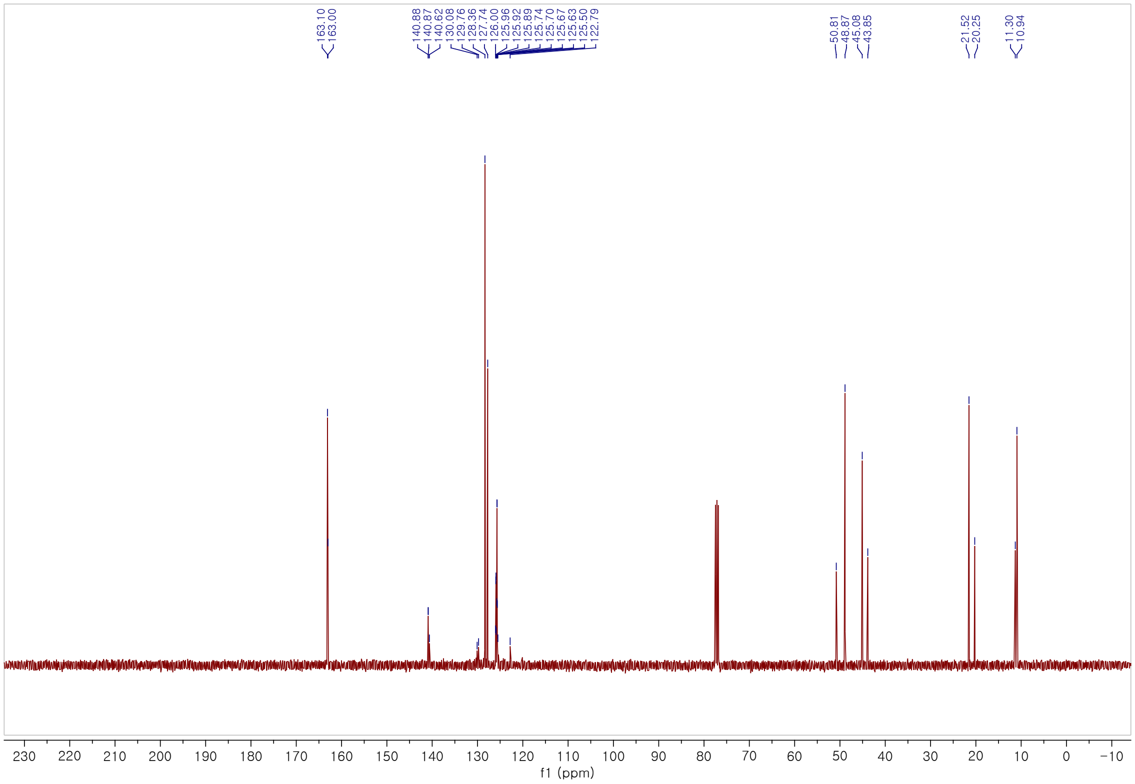
163.10
163.00
140.88
140.87
140.62
130.08
129.76
128.36
127.74
126.00
125.96
125.92
125.89
125.74
125.70
125.67
125.63
125.50
122.79
50.81
48.87
45.08
43.85
21.52
20.25
11.30
10.94
f1 (ppm)

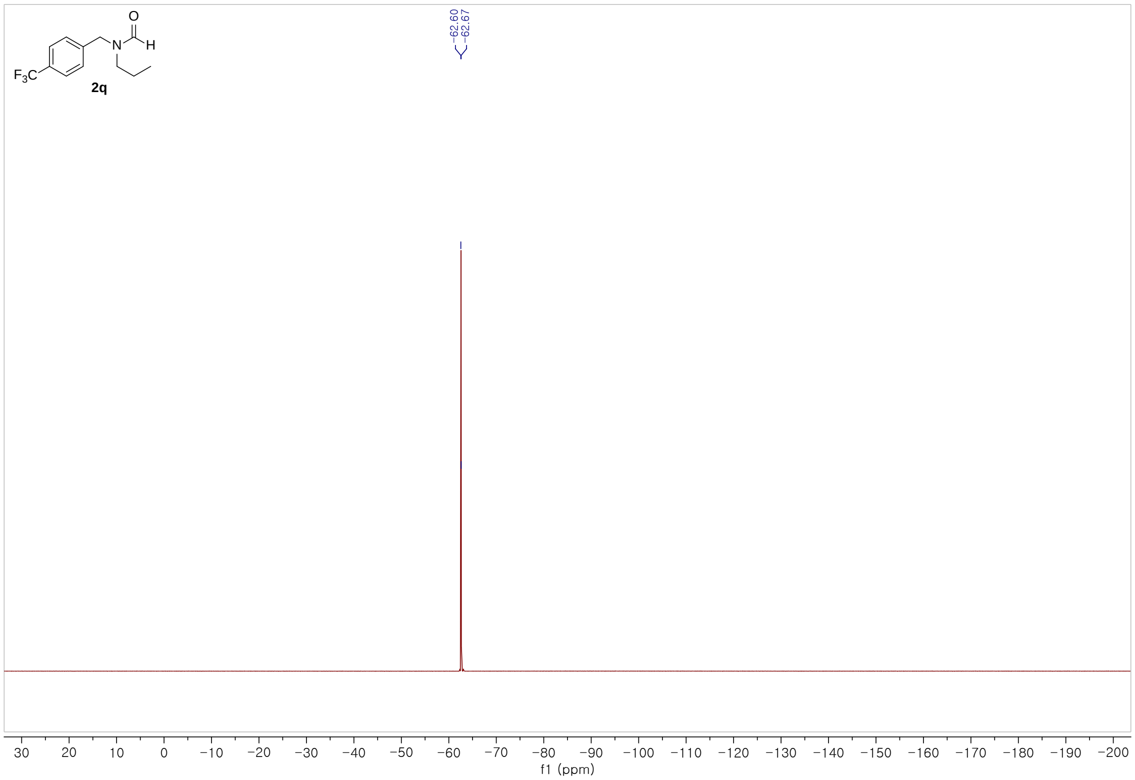
-62.60
-62.67
2q
f1 (ppm)

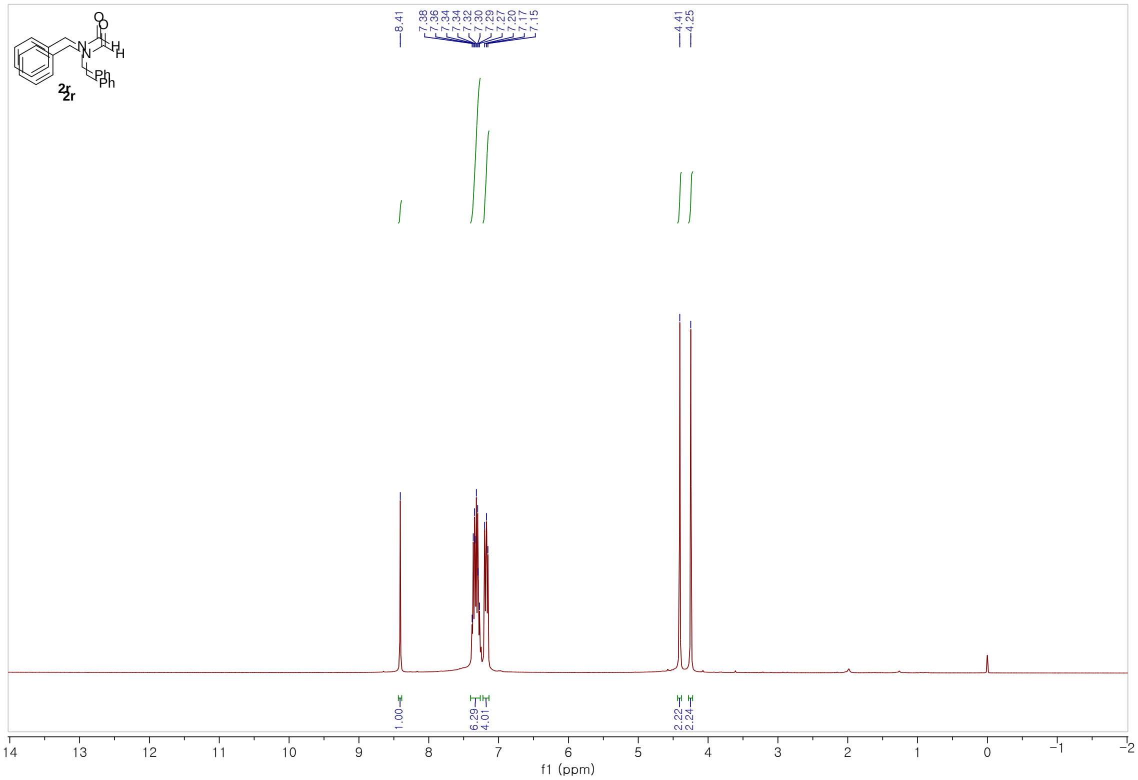

2r
8.41
7.38 7.36 7.34 7.32 7.30 7.29 7.27 7.20 7.17 7.15
4.41 4.25
1.00 6.29 4.01 2.22 2.24
f1 (ppm)

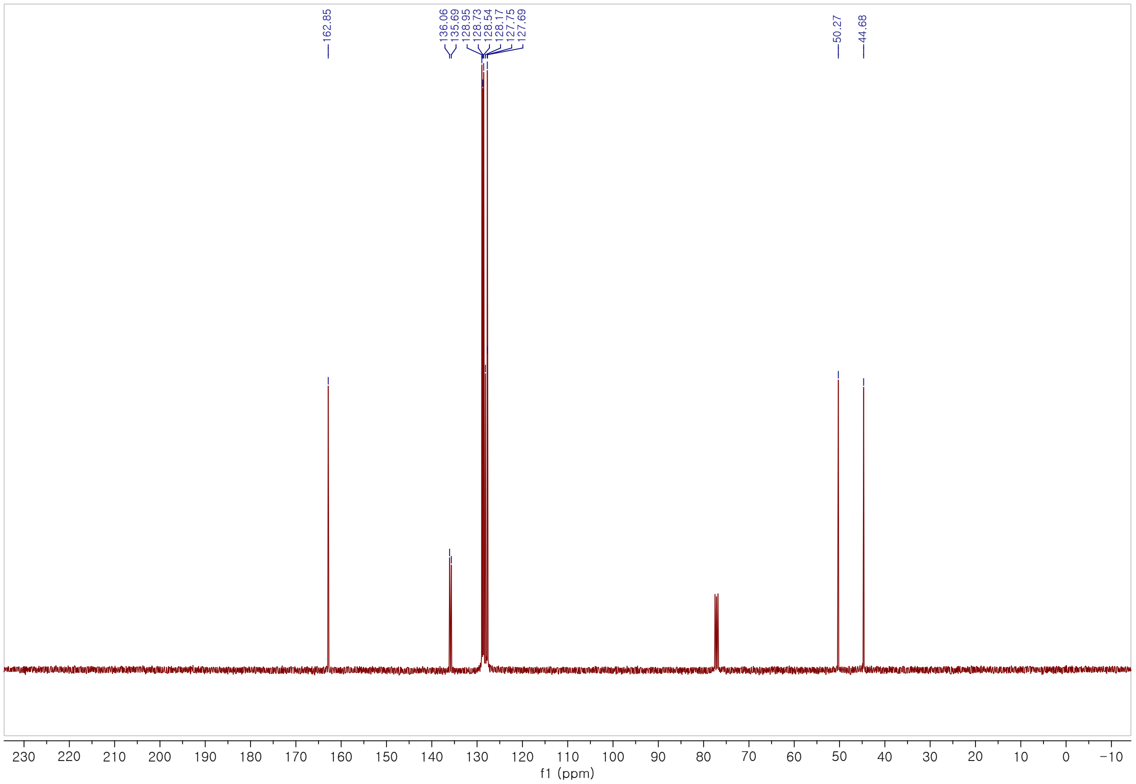

162.85
136.06 135.69 128.95 128.73 128.54 128.17 127.75 127.69
50.27 44.68
f1 (ppm)

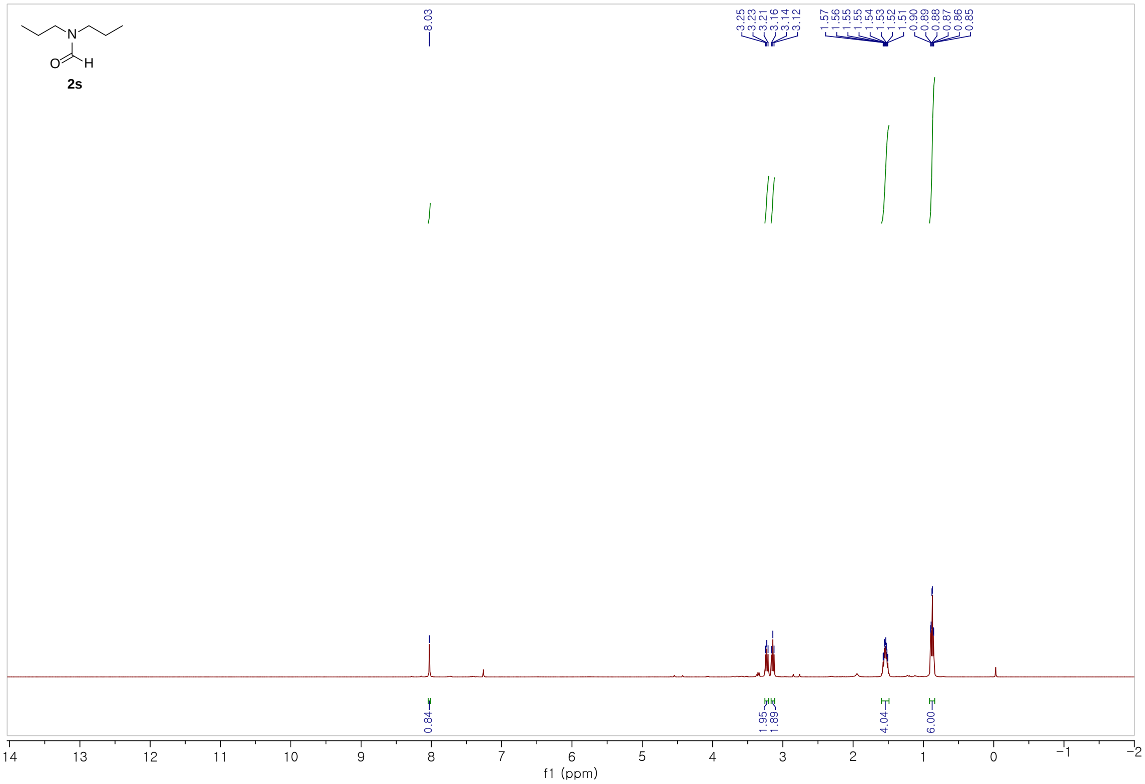
N
O
H
2s
8.03
3.25
3.23
3.21
3.16
3.14
3.12
1.57
1.56
1.55
1.55
1.54
1.53
1.52
1.51
0.90
0.89
0.88
0.87
0.86
0.85
0.84
1.95
1.89
4.04
6.00
14 13 12 11 10 9 8 7 6 5 4 3 2 1 0 −1 −2
f1 (ppm)

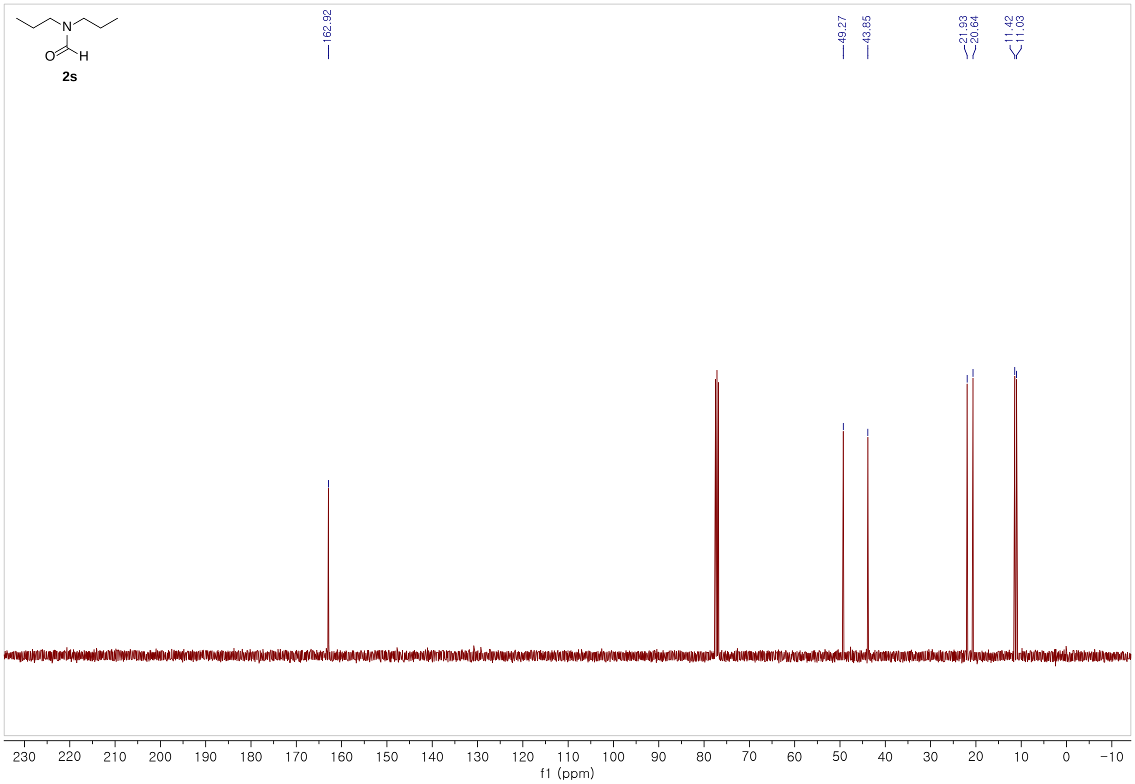
N
O
H
2s
162.92
49.27
43.85
21.93
20.64
11.42
11.03
230 220 210 200 190 180 170 160 150 140 130 120 110 100 90 80 70 60 50 40 30 20 10 0 −10
f1 (ppm)

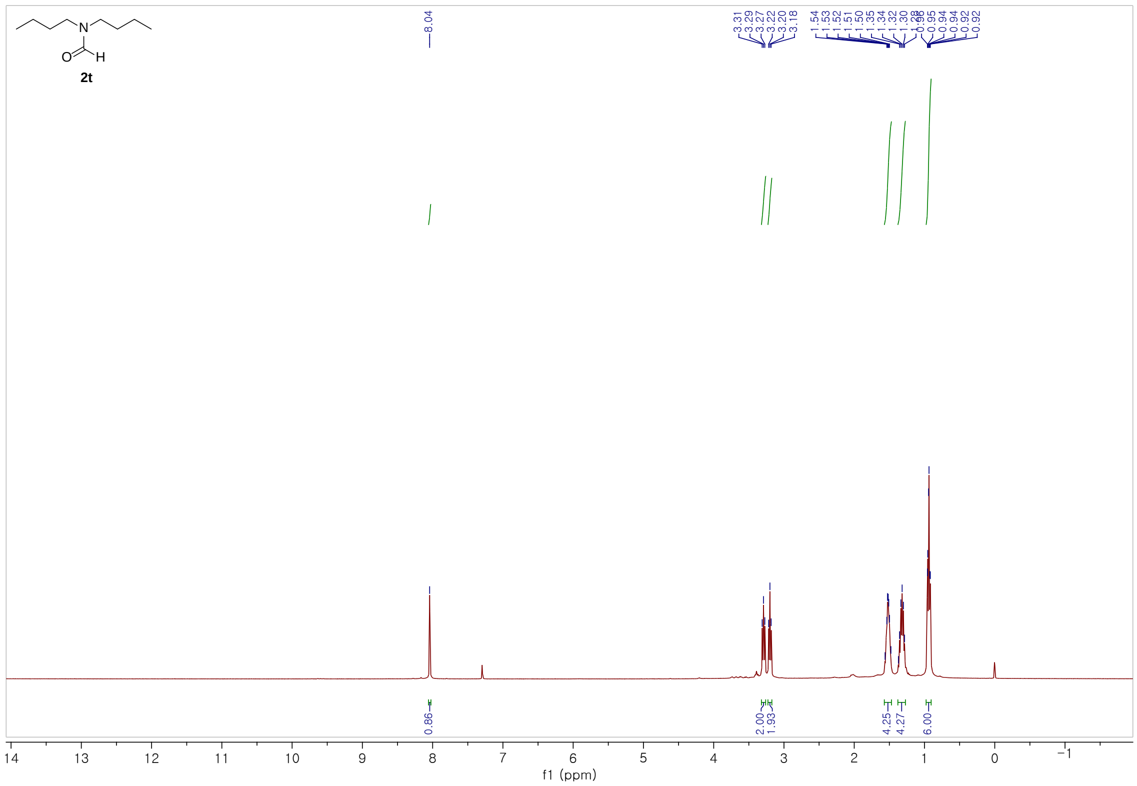
N
O
H
2t
8.04
3.31
3.29
3.27
3.22
3.20
3.18
1.54
1.53
1.52
1.51
1.50
1.35
1.34
1.32
1.30
0.96
0.95
0.94
0.94
0.92
0.92
0.86
2.00
1.93
4.25
4.27
6.00
f1 (ppm)

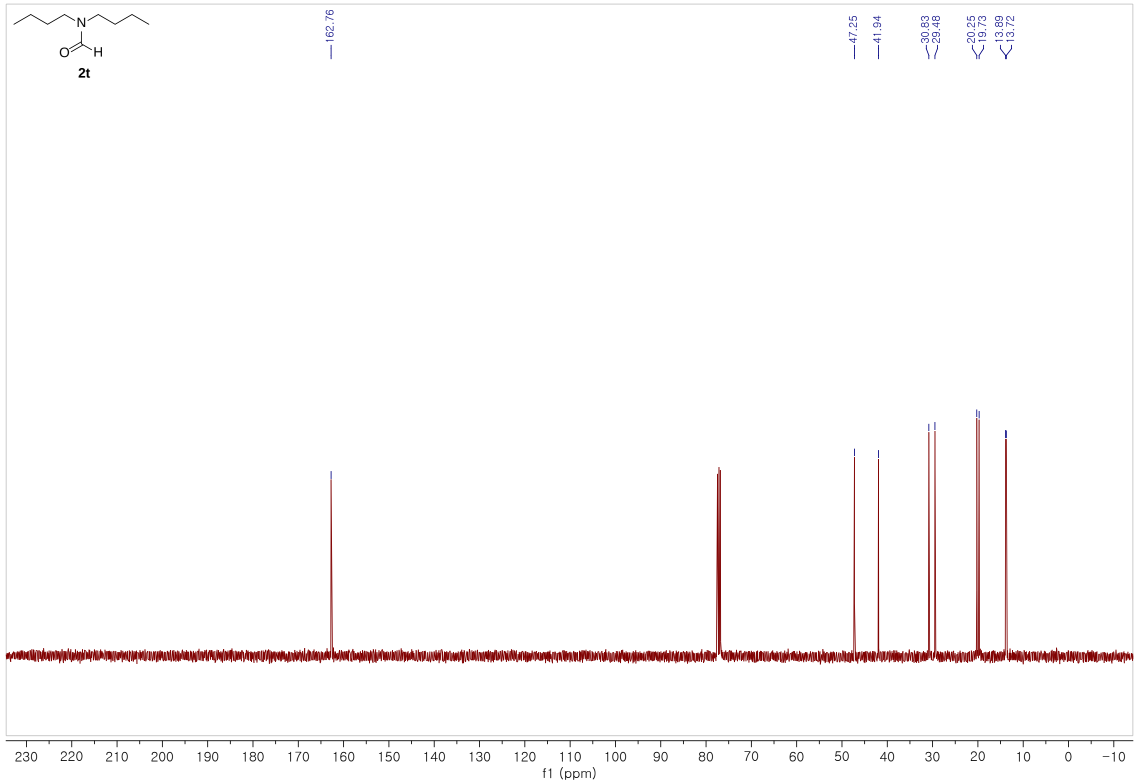
N
O
H
2t
162.76
47.25
41.94
30.83
29.48
20.25
19.73
13.89
13.72
f1 (ppm)

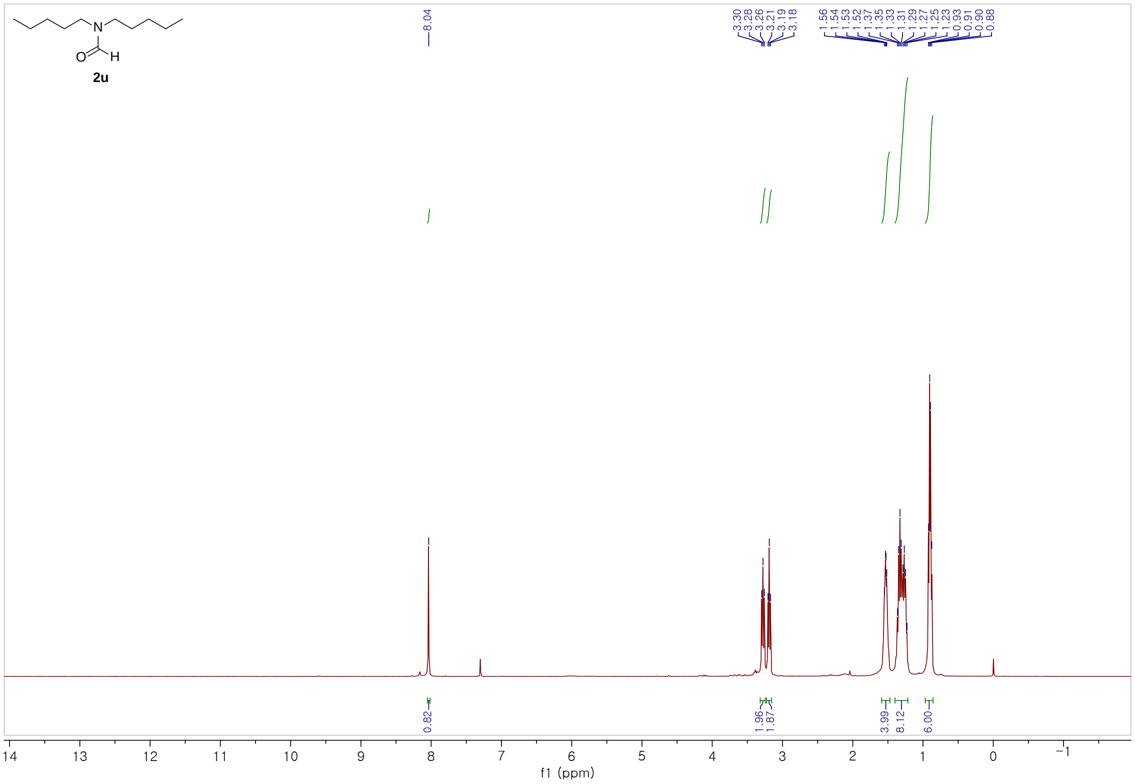
N
O
H
2u
8.04
3.30
3.28
3.26
3.21
3.19
3.18
1.56
1.54
1.53
1.52
1.37
1.35
1.33
1.31
1.29
1.27
1.25
1.23
0.93
0.91
0.90
0.88
0.82
1.96
1.87
3.99
8.12
6.00
14
13
12
11
10
9
8
7
6
5
4
3
2
1
0
−1
f1 (ppm)

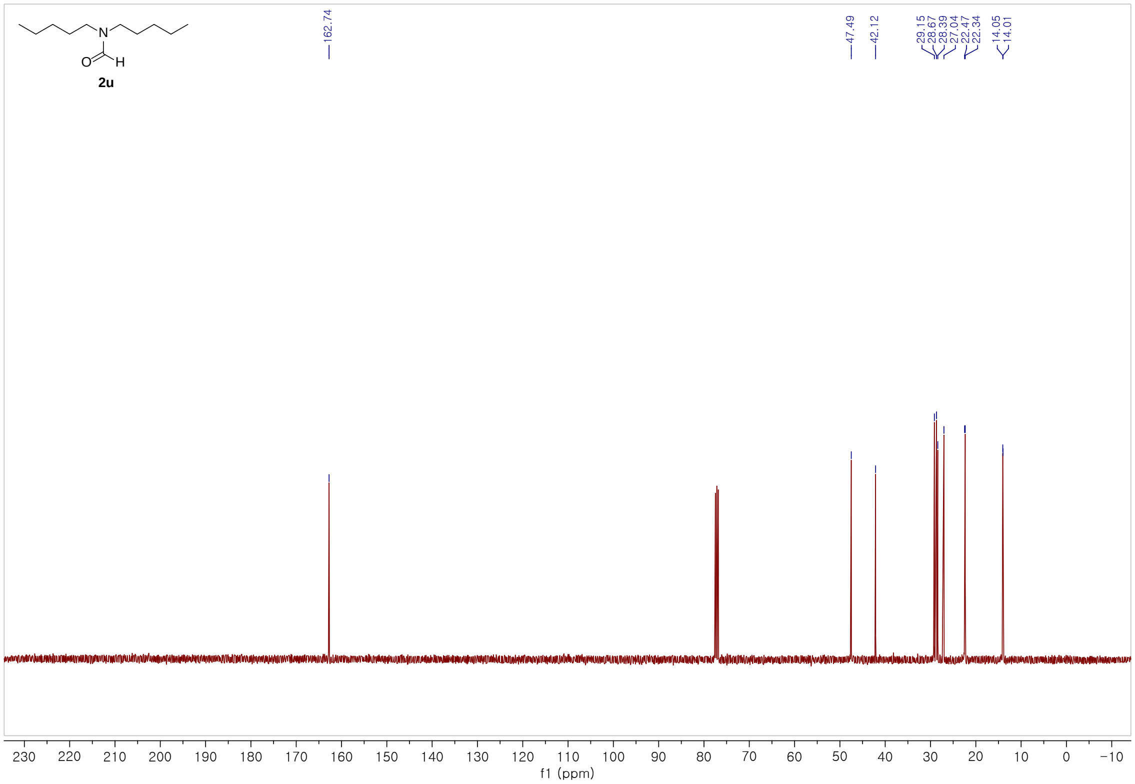
N
O
H
2u
162.74
47.49
42.12
29.15
28.67
28.39
27.04
22.47
22.34
14.05
14.01
230
220
210
200
190
180
170
160
150
140
130
120
110
100
90
80
70
60
50
40
30
20
10
0
−10
f1 (ppm)
